# Gene-diet interaction effects on BMI levels in the Singapore Chinese population

**DOI:** 10.1186/s12937-018-0340-3

**Published:** 2018-02-24

**Authors:** Xuling Chang, Rajkumar Dorajoo, Ye Sun, Yi Han, Ling Wang, Chiea-Chuen Khor, Xueling Sim, E-Shyong Tai, Jianjun Liu, Jian-Min Yuan, Woon-Puay Koh, Rob M. van Dam, Yechiel Friedlander, Chew-Kiat Heng

**Affiliations:** 1 Department of Paediatrics, Yong Loo Lin School of Medicine, National University of Singapore; and Khoo Teck Puat - National University Children’s Medical Institute, National University Health System, NUHS Tower Block, Level 12, 1E Kent Ridge Road, Singapore, 119228 Singapore; 2 Genome Institute of Singapore, Agency for Science, Technology and Research, Singapore, Singapore; 3 Department of Psychological Medicine, Yong Loo Lin School of Medicine, National University of Singapore, Singapore, Singapore; 4 Saw Swee Hock School of Public Health, National University of Singapore, Singapore, Singapore; 5 Department of Medicine, Yong Loo Lin School of Medicine, National University of Singapore, Singapore, Singapore; 6 Department of Epidemiology, Graduate School of Public Health; and University of Pittsburgh Cancer Institute, University of Pittsburgh, Pittsburgh, Pennsylvania USA; 7 Duke-NUS Medical School Singapore, Singapore, Singapore; 8 School of Public Health and Community Medicine, Hebrew University of Jerusalem, Jerusalem, Israel; 9 Unit of Epidemiology, Hebrew University-Hadassah Braun School of Public Health, POB 12272, 91120 Jerusalem, Israel

**Keywords:** Gene-diet interaction study, Body mass index, Diet, Obesity

## Abstract

**Background:**

Recent genome-wide association studies (GWAS) have identified 97 body-mass index (BMI) associated loci. We aimed to evaluate if dietary intake modifies BMI associations at these loci in the Singapore Chinese population.

**Methods:**

We utilized GWAS information from six data subsets from two adult Chinese population (*N* = 7817). Seventy-eight genotyped or imputed index BMI single nucleotide polymorphisms (SNPs) that passed quality control procedures were available in all datasets. Alternative Healthy Eating Index (AHEI)-2010 score and ten nutrient variables were evaluated. Linear regression analyses between z score transformed BMI (Z-BMI) and dietary factors were performed. Interaction analyses were performed by introducing the interaction term (diet x SNP) in the same regression model. Analysis was carried out in each cohort individually and subsequently meta-analyzed using the inverse-variance weighted method. Analyses were also evaluated with a weighted gene-risk score (wGRS) contructed by BMI index SNPs from recent large-scale GWAS studies.

**Results:**

Nominal associations between Z-BMI and AHEI-2010 and some dietary factors were identified (*P* = 0.047-0.010). The BMI wGRS was robustly associated with Z-BMI (*P* = 1.55 × 10^− 15^) but not with any dietary variables. Dietary variables did not significantly interact with the wGRS to modify BMI associations. When interaction analyses were repeated using individual SNPs, a significant association between cholesterol intake and rs4740619 (*CCDC171*) was identified (β = 0.077, adjP_interaction_ = 0.043).

**Conclusions:**

The *CCDC171* gene locus may interact with cholesterol intake to increase BMI in the Singaporean Chinese population, however most known obesity risk loci were not associated with dietary intake and did not interact with diet to modify BMI levels.

**Electronic supplementary material:**

The online version of this article (10.1186/s12937-018-0340-3) contains supplementary material, which is available to authorized users.

## Background

The explosion in worldwide obesity levels is believed to be due to the modern ‘obesogenic’ environment where there is easy access to highly appetizing and energy-dense food with a reduced need for physical activity and energy expenditure [1]. Nevertheless, substantial between-individual variability exists in the ease and extent of weight gain or weight loss, and not every individual exposed to this ‘obesogenic’ environment becomes overweight or obese. The overall effects of individual weight change is likely a composite of inherent genetic predispositions and their interaction with the environment [1–3].

Genome-wide association studies (GWAS) have successfully uncovered at least 97 independent loci associated with body mass index (BMI) levels [4] and the majority of these loci are known to be transferrable to the Asian populations [4, 5]. Studies have further suggested that dietary intake may interact at some of these loci to modify BMI levels [6–12]. However, these dietary intake interaction analyses have been predominantly performed in European ancestry populations, and since the dietary pattern of Asians is different (higher in carbohydrates) from Europeans [13], it is unclear if similar gene x diet interactions affect obesity levels in Asian populations. In this study, using Chinese subjects living in Singapore, we aimed to evaluate if dietary intake modifies obesity associations at index BMI loci, several of which have been very recently identified and not previously evaluated for in similar interaction analyses.

## Methods

### Study population

We studied 7817 participants from six data subsets from two adult Chinese populations, Singapore Chinese Health Study (SCHS), including the SCHS coronary artery disease (SCHS-CAD) cases (*N* = 594) and controls (*N* = 1070), SCHS-Type 2 diabetes (SCHS-T2D) cases (*N* = 2004) and controls (*N* = 2055), and Singapore Prospective Study Programme (SP2, *N* = 2094). Since the samples were genotyped on various SNP arrays and BMI is a known to associate with CAD and T2D [14, 15], we performed analysis individually in these six data subsets and combined individual results using meta-analysis procedures.

### Singapore Chinese Health Study

The SCHS is a population-based long term prospective study focused on the role of diet and nutrition on disease in Singapore [16, 17] A total of 63,257 Chinese individuals aged between 45 and 74 years (mean age at entry 56.5) and from two major dialect groups in Singapore, the Cantonese and the Hokkiens, were recruited into SCHS from April 1993 to December 1998. At recruitment, all the study subjects were interviewed in-person at home by a trained interviewer with a structured questionnaire. In April 1994, a 3% random sample of study subjects was re-contacted for donation of blood specimens and the effort was later extended to include all consenting cohort enrollees, which led to the collection of blood in 28,439 participants by 2001. The study was approved by the Institutional Review Boards (IRB) of the National University of Singapore (NUS) and the University of Minnesota (UMN), and all study subjects gave written informed consent.

In this study, two case-control studies conducted within the SCHS, the SCHS-CAD and the SCHS-T2D were included for analysis. SCHS-CAD participants provided blood and did not have a history of physician-diagnosed coronary heart disease (CHD) or stroke at the time of blood collection. Acute myocardial infarction (AMI) cases and coronary heart disease (CHD) deaths were identified and verified in SCHS from three databases [18, 19]. Two controls that were alive and free of the disease at the time of the diagnosis or death of the index case were matched to each CAD case on year of recruitment, date of birth, gender, father’s dialect group and the date of blood collection. In total, there were 761 incident cases and 1400 controls (*N* = 2161). For SCHS-T2D, individuals with prevalent diabetes, cardiovascular disease, or cancer at the baseline interview were excluded from analysis. Participants were classified as incident T2D cases if initial diagnosis of diabetes took place after the baseline interview and the disease states were validated as previously described [20, 21]. In total, there were 2615 incident diabetes cases and 2615 controls matched on age, gender, dialect group and date of blood collection. Detail information regarding these two sub-cohorts has been described elsewhere [22–26].

### Singapore Prospective Study Program

The SP2 is a population-based cross-sectional study of adult Singaporean Chinese, Malay and Asian-Indian subjects aged between 24 to 95 years, and it comprises four previous studies, Thyroid and Heart Study (1982–1984) [27], National Health Survey (1992) [28], National University of Singapore Heart Study (1993–1995) [29], and the National Health Survey (1998) [30] (*N* = 11,053). Individuals in these studies were sampled randomly from the Singapore population and a disproportionate sampling scheme was utilized to increase the sample sizes of Malays and Asian-Indians. In total, 7742 individuals completed the questionnaire and 5157 of them (66.6% of individuals with completed questionnaire) attended the subsequent clinical examination [31]. Only Chinese samples from the SP2 were used in the present study. The study was approved by the IRB of NUS and the Singapore General Hospital. All participants gave informed written consent before the study.

### Body composition and dietary data

In SCHS, weight and height were self-reported via in-person interviews [32, 33] and were shown to be reliable across populations [34], including Asians [35]. In SP2, a wall mounted measuring tape and a digital scale were used to measure height and weight respectively [36]. BMI was calculated as weight in kilograms (kg) divided by height in meter square (m^2^).

In SCHS, information on dietary components during the year prior to the interview was collected by using a semi-quantitative food-frequency questionnaire (FFQ) specifically developed for this population during the baseline interview. A total of 165 food items commonly consumed by Singapore Chinese subjects were assessed by the questionnaire, and the study participants provided the usual intake frequency (ranging from never or hardly ever to two or more times/d) and portion size for each of the food and beverage items. The FFQ was subsequently validated against a series of 24-h dietary recall interviews [16]. The corrected correlation coefficients for selected energy or nutrients ranged from 0.24 to 0.79 [16, 20].

In SP2, a similar semi-quantitative 169-item FFQ which was used in the Singapore National Nutrition Surveys was utilized to collect dietary intake information during the month prior to the interview [36, 37]. The estimation of the frequency for consuming each food based on a standard portion size specific for that food group was requested from the participants. The consumption frequency could be reported as per day, per week, per month, rarely or never. Nutrient intakes were computed by the Health Promotion Board of Singapore by use of an in-house database [38, 39].

The ten dietary variables examined in this study were: total calories (kcal/day), percentage of energy from protein (%protein), percentage of energy from fat (%fat), percentage of energy from saturated fatty acid (%SFA), percentage of energy from monounsaturated fatty acid (%MUFA), percentage of energy from polyunsaturated fatty acid (%PUFA), percentage of energy from carbohydrates (%carbohydrate), percentage of energy from starch (%starch), dietary fiber (g/day) and cholesterol (mg/day).

The dietary score included in this study is the Alternative Healthy Eating Index (AHEI)-2010, which is a measurement for diet quality that has been used in the Singapore Chinese population previously [40]. Detailed information about the calculation of this score has been described previously [41, 42].

### SNP selection, genotyping and imputation

Large-scale GWAS has identified 97 independent BMI-associated loci in European ancestry population [4]. Among them, 78 SNPs were either genotyped or imputed in all datasets. Detailed information about these SNPs is presented in Additional file 1: Table S1.

After standard GWAS quality control (QC) procedure, 719 SCHS CAD cases and 1284 SCHS controls genotyped on Illumina Omni-Zhonghua8 Array were utilized in the study [23–25]. For the SCHS-T2D samples, 2004 cases and 2055 controls genotyped on Affymetrix ASI (Asian) Axiom array were available for analysis [26]. A total of 4059 individuals was left for subsequent analysis after QC. After QC procedure, 1145 Chinese SP2 individuals genotyped using Human Hap 610Quad (SP2610) and 949 genotyped with Illumina 1Mduov3 (SP21m) were available for analysis [22]. Imputation in both SCHS and SP2 was performed with IMPUTE2 [43] and genotype calls were based on phase3 1000G cosmopolitan panels.

### Statistical analysis

A weighted genetic risk score (wGRS) was calculated based on the 78 BMI-associated variants, where the number of BMI increasing alleles were weighted by their reported effect estimates from recent large-scale GWAS [4]. Intakes of protein, fat, SFA, MUFA, PUFA, carbohydrate and starch were adjusted for total energy intake by converting to nutrient densities. Cholesterol and fiber were converted to calorie-adjusted nutrient values based on the method of residuals [44]. BMI and all the nutrient variables were normalized by rank-based inverse normalization (Z-scores). Linear regression analyses between Z-BMI and dietary factors were performed and adjusted for age, sex and calorie intake. Association between SNPs and BMI/dietary components were evaluated by linear regression with adjustment for age and gender. Interaction analyses were performed by introducing the interaction term (dietary factor x SNP) with the specific dietary factor and SNP included as covariates in the same regression model. Analysis was carried out in each cohort individually and subsequently meta-analyzed using the fixed-effects inverse-variance weighted method. Cochran’s Q test was used to measure between-study heterogeneity (*P* < 0.050) [45]. All analyses were performed using STATA (version 12.1, Statacorp, College Station, TX, USA). Bonferroni adjusted *P* value of < 0.05 (2 tailed) was considered statistically significant after adjusting for multiple comparison for 858 tests (78 BMI SNPs × 11 dietary variables).

## Result

The characteristics of variables used in the study are presented in Table 1. In total, 7817 individuals (5723 from SCHS and 2094 from SP2) had data available for analysis.

**Table 1:** Characteristics of the study participants

	SCHS CAD cases *N* = 594	SCHS CAD controls *N* = 1070	SP2610 *N* = 1145	SP21m *N* = 949	SCHS T2D cases *N* = 2004	SCHS T2D controls *N* = 2055
Ethnicity	Chinese	Chinese	Chinese	Chinese	Chinese	Chinese
BMI (kg/m^2^)	23.25 ± 3.48	22.83 ± 3.27	22.62 ± 3.88	22.89 ± 3.47	24.88 ± 3.57	22.62 ± 3.34
Age (years)	66.14 ± 7.83	65.98 ± 7.78	48.48 ± 11.42	46.76 ± 10.36	61.89 ± 7.09	61.74 ± 7.14
Gender (male %)	389 (65.49%)	699 (65.33%)	268 (23.41%)	605 (63.75%)	974 (48.60%)	964 (46.91%)
Calories (kcal/day)	1642.12 ± 600.70	1638.09 ± 561.71	1853.89 ± 761.45	2115.58 ± 839.03	1638.43 ± 591.61	1585.10 ± 547.15
%Protein^a^	15.02 ± 2.51	15.09 ± 2.50	15.08 ± 2.02	14.80 ± 1.95	15.33 ± 2.48	15.25 ± 2.48
%Fat^a^	24.86 ± 5.80	24.90 ± 5.56	28.30 ± 5.70	28.63 ± 5.63	25.56 ± 5.60	25.52 ± 5.51
%SFA^a^	8.75 ± 2.50	8.73 ± 2.55	10.58 ± 2.62	10.77 ± 2.67	9.00 ± 2.51	8.93 ± 2.53
%MFA^a^	8.49 ± 2.13	8.41 ± 2.07	9.87 ± 2.63	10.01 ± 2.57	8.61 ± 2.04	8.56 ± 2.04
%PFA^a^	4.98 ± 1.82	5.13 ± 1.85	6.04 ± 2.52	5.97 ± 2.44	5.24 ± 2.01	5.32 ± 1.94
%Carbohydrate^a^	59.38 ± 7.57	59.14 ± 7.23	56.40 ± 6.57	56.17 ± 6.59	58.55 ± 7.27	58.76 ± 7.18
%Starch^a^	42.90 ± 9.42	42.10 ± 9.25	35.90 ± 7.48	36.55 ± 7.92	41.25 ± 9.21	40.99 ± 9.22
Fiber (g/day)	12.86 ± 5.43	13.19 ± 5.75	19.77 ± 8.50	21.92 ± 9.29	13.31 ± 5.92	13.24 ± 5.66
Cholesterol (mg/day)	184.69 ± 114.18	184.35 ± 116.00	225.94 ± 139.26	260.61 ± 150.56	186.39 ± 110.02	174.26 ± 101.23
AHEI-2010	49.45 ± 7.45	50.67 ± 7.47	51.14 ± 8.16	51.46 ± 8.15	50.09 ± 7.36	51.03 ± 7.54
wGRS	66.68 ± 5.13	66.69 ± 5.26	66.57 ± 5.02	66.53 ± 5.12	67.68 ± 4.95	67.17 ± 5.08
Red meat						
Unprocessed (servings/day)	0.28 ± 0.22	0.26 ± 0.21	0.44 ± 0.45	0.54 ± 0.57	0.27 ± 0.21	0.25 ± 0.19
Processed (servings/day)	0.06 ± 0.10	0.06 ± 0.10	0.12 ± 0.25	0.14 ± 0.24	0.07 ± 0.12	0.06 ± 0.12
Total (servings/day)	0.35 ± 0.27	0.32 ± 0.25	0.56 ± 0.57	0.68 ± 0.66	0.34 ± 0.28	0.31 ± 0.26
Egg yolk (grams/day)	4.74 ± 5.28	4.68 ± 5.49	10.69 ± 8.08	12.47 ± 8.61	4.60 ± 4.75	4.21 ± 4.37

*T2D* Type 2 diabetes, *SFA* Saturated Fatty Acids, *PUFA* Polyunsaturated Fatty Acids, *MUFA* Monounsaturated Fatty Acids, *AHEI* Alternative Healthy Eating Index

Data was presented as Mean ± SD or N (%)

One serving is 4 oz. (113.4 g) of unprocessed meat and 1.5 oz. (42.5 g) of processed meat (1 oz. = 28.35 g)

^a^Represented as a *%* of Total Energy

### Association between dietary factors with BMI levels

The following dietary factors, AHEI-2010, total calories, %protein, %fat, %carbohydrate %starch, and cholesterol showed nominal significance with Z-BMI (P between 0.047 and 0.010) (Table 2). Higher calories, %protein, %fat and cholesterol were associated with increased BMI while higher AHEI, %starch and %carbohydrate were associated with lower BMI. However, none of these remained significant after corrections for multiple tests (adj *p*-value > 0.110, Table 2).

**Table 2:** Association between dietary factors and BMI level

	SCHS CAD cases N = 594			SCHS CAD controls *N* = 1070			SP2610 *N* = 1145			SP21m *N* = 949			SCHS T2D cases *N* = 2004			SCHS T2D controls *N* = 2055			Meta-analysis of all datasets *N* = 7817				
	Beta	SE	P	Beta	SE	P	Beta	SE	P	Beta	SE	P	Beta	SE	P	Beta	SE	P	Beta	SE	P	P_adjust_	Q_*p*-value_
AHEI-2010	−0.002	0.005	0.650	0.002	0.004	0.666	−0.008	0.004	0.032	0.001	0.004	0.730	−0.007	0.003	0.021	−0.003	0.003	0.318	−0.003	0.001	0.019	0.209	0.315
Calories	0.142	0.045	0.002	0.083	0.033	0.012	−0.002	0.030	0.952	0.026	0.033	0.437	−0.015	0.024	0.542	0.017	0.024	0.489	0.025	0.012	0.037	0.407	0.019
%oProtein^a^	0.049	0.041	0.234	0.031	0.031	0.325	0.033	0.029	0.264	0.048	0.033	0.147	0.009	0.023	0.693	0.019	0.023	0.402	0.026	0.012	0.023	0.253	0.918
%Fat^a^	− 0.008	0.042	0.848	0.084	0.032	0.008	0.009	0.033	0.773	0.08	0.036	0.028	−0.009	0.024	0.711	0.021	0.023	0.363	0.025	0.012	0.039	0.429	0.123
%SFA^a^	− 0.001	0.041	0.985	0.075	0.031	0.017	−0.004	0.032	0.904	0.042	0.037	0.248	0.001	0.023	0.971	0.018	0.023	0.426	0.020	0.012	0.096	1.000	0.424
%MFA^a^	0.002	0.042	0.967	0.044	0.032	0.162	0.035	0.031	0.258	0.086	0.035	0.013	−0.002	0.023	0.928	0.005	0.023	0.811	0.022	0.012	0.058	0.638	0.310
%PFA^a^	− 0.049	0.041	0.232	0.054	0.031	0.080	−0.011	0.030	0.721	0.079	0.033	0.017	−0.017	0.023	0.468	0.016	0.023	0.475	0.012	0.012	0.313	1.000	0.065
%Carbohydrate^a^	0.005	0.042	0.904	− 0.065	0.032	0.039	−0.010	0.032	0.762	−0.063	0.035	0.071	−0.003	0.023	0.905	−0.020	0.023	0.377	−0.023	0.012	0.047	0.517	0.480
%Starch^a^	− 0.012	0.041	0.777	−0.077	0.031	0.015	−0.012	0.030	0.565	−0.100	0.033	0.003	−0.012	0.023	0.612	−0.003	0.023	0.888	−0.030	0.011	0.010	0.110	0.108
Fiber	0.029	0.041	0.484	0.023	0.031	0.445	−0.011	0.030	0.716	0.018	0.033	0.593	−0.039	0.023	0.089	−0.022	0.023	0.347	−0.009	0.012	0.421	1.000	0.463
Cholesterol	0.054	0.041	0.192	0.020	0.031	0.528	0.062	0.030	0.035	0.049	0.033	0.136	0.010	0.023	0.675	−0.005	0.022	0.823	0.023	0.011	0.042	0.462	0.433

*Q*_*p-value*_ Cochran’s Q heterogeneity measure, *SFA* Saturated Fatty Acids, *PUFA* Polyunsaturated Fatty Acids, *MUFA* Monounsaturated Fatty Acids, *AHEI* Alternative Healthy Eating Index, *SCHS* Singapore Chinese Health Study, *SP2* Singapore Prospective Study Program, *T2D* Type II diabetes

Results are from meta-analysis of SCHS MI cases (*N* = 594) and control datasets (*N* = 1070), SP2 610 (*N* = 1145) and 1 M datasets (*N* = 949), SCHS Type 2 diabetes cases (*N* = 2004) and control datasets (*N* = 2055)

Age gender and total calories intake were included in the linear regression model as covariates, except for BMI, AHEI-2010 and calories

^a^Represented as a % of Total Energy

### Association between BMI index SNPs with BMI and dietary factors

Linear regression analyses were used to test the association between BMI index SNPs and Z-BMI. Among the 78 overlapping SNPs, 9 loci (*TMEM18, GNPDA2, RALYL, NT5C2, OLFM4, FTO, MC4R, QPCTL* and *ZC3H4*) were significantly associated with the outcome (*P* < 0.05, Additional file 1: Table S1).

A wGRS was constructed using all 78 BMI index SNPs. Each unit increase in the wGRS was robustly associated with increased Z-BMI in our datasets (β = 0.018, SE = 0.002, *P* = 1.55 × 10^− 15^, Table 3). The aggregate BMI wGRS however did not show significant associations with any dietary factors (*p* > 0.190, Table 3).

**Table 3:** Association between wGRS and dietary factors or BMI

	SCHS CAD cases *N* = 594			SCHS CAD controls *N* = 1070			SP2610 N = 1145			SP21m N = 949			SCHS T2D cases *N* = 2004			SCHS T2D controls *N* = 2055			Meta-analysis of all datasets *N* = 7817				
	Beta	SE	P	Beta	SE	P	Beta	SE	P	Beta	SE	P	Beta	SE	P	Beta	SE	P	Beta	SE	P	P_adjust_	Q_*p*-value_
BMI	0.018	0.008	0.023	0.025	0.006	< 0.001	0.019	0.006	0.001	0.019	0.006	0.002	0.010	0.004	0.023	0.018	0.004	< 0.001	0.018	0.002	1.55x10^15^		0.488
AHEI-2010	0.037	0.060	0.535	−0.060	0.043	0.163	− 0.016	0.048	0.736	0.114	0.051	0.025	−0.031	0.033	0.353	−0.025	0.033	0.449	−0.010	0.017	0.540	1.000	0.130
Calories	−0.001	0.007	0.892	− 0.011	0.005	0.043	0.005	0.006	0.369	−0.005	0.006	0.407	−0.003	0.004	0.445	−0.001	0.004	0.763	− 0.003	0.002	0.190	1.000	0.468
%Protein^a^	− 0.010	0.008	0.222	−0.005	0.006	0.409	0.004	0.006	0.507	0.005	0.006	0.443	0.005	0.004	0.212	0.002	0.004	0.691	0.002	0.002	0.490	1.000	0.488
%Fat^a^	−0.001	0.008	0.933	3.96 × 10^04^	0.006	0.945	0.010	0.006	0.080	−0.004	0.006	0.522	0.003	0.004	0.430	2.93 × 10^−04^	0.004	0.944	0.002	0.002	0.383	1.000	0.646
%SFA^a^	− 0.001	0.008	0.899	0.0028	0.006	0.717	0.002	0.006	0.697	− 0.003	0.006	0.566	0.001	0.004	0.863	0.002	0.004	0.620	0.001	0.002	0.700	1.000	0.980
%MFA^a^	0.002	0.008	0.826	−0.001	0.006	0.810	0.010	0.006	0.083	−0.007	0.006	0.235	4.78 × 10^−04^	0.004	0.913	−0.001	0.004	0.727	1.95E-04	0.002	0.929	1.000	0.460
%PFA^a^	− 0.002	0.008	0.823	−0.004	0.006	0.524	0.005	0.006	0.384	0.004	0.006	0.523	0.006	0.004	0.171	−0.001	0.004	0.812	0.002	0.002	0.420	1.000	0.715
%Carbohydrate^a^	0.004	0.008	0.605	0.002	0.006	0.757	−0.010	0.006	0.085	0.002	0.006	0.764	−0.002	0.004	0.622	−0.002	0.004	0.704	−0.002	0.002	0.466	1.000	0.656
%Starch^a^	0.001	0.008	0.862	0.008	0.006	0.182	−0.003	0.006	0.640	−3.90 × 10^− 04^	0.006	0.951	−0.001	0.004	0.768	−0.004	0.004	0.402	−4.70 × 10^− 04^	0.002	0.829	1.000	0.734
Fiber	0.004	0.008	0.592	−0.006	0.006	0.292	−0.001	0.006	0.848	0.006	0.006	0.362	−0.004	0.004	0.349	0.001	0.004	0.892	−0.001	0.002	0.692	1.000	0.698
Cholesterol	−0.013	0.008	0.092	0.003	0.006	0.624	0.006	0.006	0.334	−0.005	0.006	0.443	0.01	0.004	0.030	0.001	0.004	0.733	0.002	0.002	0.287	1.000	0.141

*Q*_*p-value*_ Cochran’s Q heterogeneity measure, *SFA* Saturated Fatty Acids, *PUFA* Polyunsaturated Fatty Acids, *MUFA* Monounsaturated Fatty Acids, *AHEI* Alternative Healthy Eating Index, *SCHS* Singapore Chinese Health Study, *SP2* Singapore Prospective Study Program, *T2D* Type II diabetes

Results are from meta-analysis of SCHS MI cases (N = 594) and control datasets (*N* = 1070), SP2 610 (*N* = 1145) and 1 M datasets (*N* = 949), SCHS Type 2 diabetes cases (*N* = 2004) and control datasets (*N* = 2055)

Age gender and total calories intake were included in the linear regression model as covariates, except for BMI, AHEI-2010 and calories

^a^Represented as a % of Total Energy

### Gene-diet interaction

Inclusion of the dietary factor x wGRS in the regression models did not significantly modify their associations with BMI in our dataset (P_interaction_ > 0.112, Table 4). However, when analyzing at single SNP level (Table 5, Additional file 1: Tables S2-S12), we observed one significant interaction between rs4740619 (*CCDC171)* and cholesterol on Z-BMI even after adjusting for multiple comparisons (β = 0.077, SE = 0.019, P_interaction_ = 5.01 × 10^− 5^, adjusted P_interaction_ = 0.043, Table 5, Additional file 1: Table S12). As red meat and egg yolk are substantial sources of cholesterol [46, 47], we further analyzed whether rs4740619 could interact with red meat intake or yolk on BMI and found significant interaction between rs4740619 and processed red meat intake as well as egg yolk consumption (Additional file 1: Table S13). Conditioning the cholesterol intake x rs4740619 effects on processed red meat intake or egg yolk intake (and vice versa) did not affect the associations detected, indicating that these interactions may be independent.

**Table 4:** Interaction between GRS and dietary factors on BMI in individual datasets used in the study

	SCHS CAD cases *N* = 594			SCHS CAD controls *N* = 1070			SP2610 *N* = 1145			SP21m *N* = 949			SCHS T2D cases *N* = 2004			SCHS T2D controls *N* = 2055			Meta-analysis of all datasets *N* = 7817				
	Beta	SE	P	Beta	SE	P	Beta	SE	P	Beta	SE	P	Beta	SE	P	Beta	SE	P	Beta	SE	P	P_adjust_	Q_*p*-value_
AHEI-2010	−0.002	0.001	0.109	−5.10E-04	0.001	0.468	−4.20E-04	6.88E-04	0.537	−0.001	0.001	0.177	3.58E-04	0.001	0.561	−4.40E-04	5.72E-04	0.446	−4.50E-04	2.82E-04	0.112	1.000	0.594
Calories	0.007	0.008	0.373	−0.008	0.006	0.151	−0.003	0.006	0.620	0.001	0.006	0.929	−0.008	0.005	0.090	−0.001	0.004	0.775	−0.003	0.002	0.158	1.000	0.539
%oProtein^a^	0.001	0.008	0.885	−0.005	0.006	0.389	0.007	0.006	0.228	−0.017	0.007	0.008	−0.001	0.004	0.789	0.004	0.004	0.364	−0.001	0.002	0.689	1.000	0.077
%Fat^a^	−0.010	0.008	0.230	0.004	0.006	0.498	−0.002	0.006	0.751	−0.014	0.006	0.024	0.003	0.004	0.496	0.005	0.004	0.298	−2.80E-04	0.002	0.900	1.000	0.125
%SFA^a^	−0.011	0.008	0.185	0.006	0.006	0.334	4.74E-04	0.006	0.937	−0.007	0.006	0.246	0.004	0.005	0.415	0.006	0.004	0.166	0.002	0.002	0.450	1.000	0.301
%MFA^a^	−0.003	0.008	0.685	0.004	0.006	0.480	0.001	0.006	0.830	−0.014	0.006	0.032	−0.002	0.005	0.651	0.006	0.004	0.210	−2.00E-04	0.002	0.927	1.000	0.213
%PFA^a^	-0.007	0.008	0.413	0.005	0.006	0.337	−0.005	0.006	0.403	−0.012	0.006	0.073	0.006	0.005	0.187	−0.001	0.004	0.765	−0.001	0.002	0.771	1.000	0.201
%Carbohydrate^a^	0.007	0.008	0.412	−0.002	0.006	0.744	−0.002	0.006	0.668	0.016	0.006	0.012	−0.001	0.004	0.745	−0.005	0.004	0.288	2.61E-04	0.002	0.906	1.000	0.128
%Starch^a^	0.013	0.008	0.115	−0.001	0.006	0.883	0.001	0.006	0.850	0.015	0.007	0.026	0.001	0.005	0.758	0.002	0.004	0.721	0.003	0.002	0.125	1.000	0.372
Fiber	−0.011	0.008	0.193	−0.005	0.006	0.398	−0.002	0.006	0.708	0.003	0.006	0.639	0.003	0.005	0.557	−0.009	0.004	0.057	−0.003	0.002	0.182	1.000	0.422
Cholesterol	0.011	0.007	0.139	0.002	0.006	0.665	−0.005	0.006	0.371	−0.012	0.007	0.080	−0.003	0.005	0.463	0.008	0.004	0.082	4.72E-04	0.002	0.833	1.000	0.082

*Q*_*p-value*_ Cochran’s Q heterogeneity measure, *SFA* Saturated Fatty Acids, *PUFA* Polyunsaturated Fatty Acids, *MUFA* Monounsaturated Fatty Acids, *AHEI* Alternative Healthy Eating Index, *SCHS* Singapore Chinese Health Study, *SP2* Singapore Prospective Study Program, *T2D* Type II diabetes

Results are from meta-analysis of SCHS MI cases (N = 594) and control datasets (N = 1070), SP2 610 (N = 1145) and 1 M datasets (N = 949), SCHS Type 2 diabetes cases (N = 2004) and control datasets (N = 2055)

Age gender and total calories intake were included in the linear regression model as covariates, except for BMI, AHEI-2010 and calories

^a^Represented as a % of Total Energy

**Table 5:** Meta-analysis of the interaction between rs4740619 and dietary factor on BMI

	SCHS CAD cases *N* = 594			SCHS CAD controls *N* = 1070			SP2610 *N* = 1145			SP21m *N* = 949			SCHS T2D cases *N* = 2004			SCHS T2D controls *N* = 2055			Meta-analysis of all datasets *N* = 7817				
	Beta	SE	P	Beta	SE	P	Beta	SE	P	Beta	SE	P	Beta	SE	P	Beta	SE	P	Beta	SE	P	P adjust	Q_*p*-value_
rs4740619 x cholesterol	0.087	0.066	0.186	0.109	0.053	0.039	0.077	0.049	0.119	0.103	0.056	0.064	0.033	0.037	0.381	0.092	0.038	0.015	0.077	0.019	5.01E-05	0.043	0.828

*Qp-value* Cochran’s Q heterogeneity measure

Interaction results are from meta-analysis of SCHS MI cases (N = 594) and control datasets (N = 1070), SP2 610 (N = 1145) and 1 M datasets (N = 949), SCHS Type 2 diabetes cases (N = 2004) and control datasets (N = 2055)

## Discussion

Ethnic differences in dietary intakes may influence the impact of inherent genetic predispositions to obesity. In this study, we evaluated the role of dietary intake, both as individual nutrient components and as a composite score (i.e AHEI-2010), on BMI levels using East-Asian subjects. To the best of our knowledge, our study represents the first systematic investigation on gene-diet interactions in East-Asians at established BMI-susceptibility loci, several of which have been only recently identified [4].

The BMI wGRS showed robust association with BMI levels in our Singapore Chinese samples and individually, most of the BMI susceptibility SNPs, were directionally consistent with their previously reported effects, indicating that genetic predisposition to obesity is largely transferrable to the Singapore Chinese population [4]. A total of nine loci (*TMEM18, GNPDA2, RALYL, NT5C2, OLFM4, FTO, MC4R, QPCTL* and *ZC3H4*) were associated with the outcome and the most strongly associated locus was rs11191560 on *NT5C2*. Nominal associations observed between the AHEI-2010 score and individual dietary components with BMI levels in our study suggest that a healthier diet may reduce obesity levels. However, using an aggregate wGRS score for all known BMI genetic loci, we find little evidence that a healthier diet may modify genetic predisposition to BMI levels in the East-Asian samples evaluated. This is similar to previous larger-scale studies in European ancestry subjects [48]. While the wGRS approach allows for the evaluation of overall genetic predisposition to BMI, it might incorporate multiple heterogeneous pathways that may not associate or interact with lifestyle factors in a similar manner. Investigating individual BMI risk SNPs of the aggregate wGRS score could therefore provide better biological insights on the complex interactions between genetic risks and dietary intake [49, 50].

Previous study in adults of European ancestry showed two BMI loci, *LRRN6C* and *MTIF3,* could modify the association between dietary score and BMI levels [8]. However, in our study, none of the reported risk loci significantly interacted with AHEI-2010. Differences in sample sizes, risk allele frequencies and/or dietary consumptions may explain these discrepancies. When evaluated at the single-SNP and individual dietary components level, our interaction analyses revealed a novel significant association between cholesterol intake and rs4740619 that increased BMI levels. This interaction was independent of red meat and egg yolk intake. Rs4740619 is an intronic variant on coiled-coil domain containing 171 (*CCDC171*), a newly identified gene on chromosome 9. HaploReg analysis indicated that rs4740619 may affect binding affinity of peroxisome proliferator-activated receptors (PPARs), which are nuclear receptors involved in regulating multiple metabolic pathways [51]. However, precisely how rs4746019 affects PPAR function and whether these are modulated by cholesterol levels will require further replication efforts and subsequent functional assessments.

Our study is not without limitations. Firstly, proxy measures of dietary intake through food-frequency questionnaire data is likely a source of random error [52] and as previously highlighted, may be amplified in the context of obesity due to the awareness between diet and corpulence [8]. Moreover, due to the relatively modest sample set evaluated in this study and likely limited statistical power, further evaluations in larger-scale and better powered East-Asian studies or specific dietary intervention studies would be necessary to confirm and better characterize the associations reported here. The AHEI-2010 score was an alternative to the HEI score, which measures the adherence to dietary recommendations among Americans. Higher AHEI was strongly associated with lower risk for a variety of chronic diseases, such as cardiovascular disease and diabetes [41, 42]. Although this score served to capture several dietary components in aggregate, it should be noted that it is not specific to the East-Asian population and may not fully capture dietary differences that exists between ethnic groups. In addition, AHEI-2010 is constructed by a simple summation of several components scored on a scale ranging between 0 and 10 and therefore had assumed that each component affects health equally, which is not the case. Thus a score calculated in a more sophisticated manner might be needed for a comprehensive assessment of dietary effects on health outcomes [53]. Lastly, recent studies have indicated that there may be an association between diet and body composition and have highlighted specific interactions between central obesity associated genetic loci and healthy diet scores (for eg. at *GRB4* and *LYPLAL1* loci) [8]. As most of the study subjects in our datasets do not have central obesity measures (i.e waist and hip circumferences), we were however unable to perform similar analyses to interrogate the interactions between diet and central obesity. Moreover, BMI is a surrogate measure of body composition. In certain situations, it might not be a valid reflection for body fat percentage, the excess of which is considered to be cause of the comorbid conditions, such as for people with well-developed musculature [54]. Nevertheless, in the general population, there is a significant positive correlation between BMI and body fat percentage, as well as with clinical outcome such as AMI, CAD and CHD mortality, including the Chinese subjects. [55–62].

## Conclusion

In conclusion, similar to studies performed in large-scale European ancestry samples, our data indicates that, in aggregate, most known BMI risk loci do not interact with dietary intake to modify BMI levels in East-Asian subjects. However, when evaluated as individual SNPs, a specific interaction at rs4740619 (*CCDC171*) with cholesterol and processed red meat intake that increases BMI levels was identified in our study subjects.

## Additional file

Additional file 1: **Table S1.** SNP information and Meta-analysis between 78 SNPs and BMI level. **Table S2.** Interaction between SNPs and AHEI-2010 dietary score on BMI. **Table S3.** Interaction between SNPs and total calories on BMI. **Table S4.** Interaction between SNPs and %protein on BMI. **Table S5.** Interaction between SNPs and %fat on BMI. **Table S6.** Interaction between SNPs and %SFA on BMI. **Table S7.** Interaction between SNPs and %MUFA on BMI. **Table S8.** Interaction between SNPs and %PUFA on BMI. **Table S9.** Interaction between SNPs and %carbohydrate on BMI. **Table S10.** Interaction between SNPs and %starch on BMI. **Table S11.** Interaction between SNPs and fiber on BMI. **Table S12.** Interaction between SNPs and cholesterol on BMI. **Table S13.** Interaction between GRS and dietary factors on BMI in individual datasets used in the study. (PDF 1521 kb)

## Abbreviations

AHEI: Alternative Healthy Eating Index
BMI: Body mass index
GWAS: Genome-wide association studies
MUFA: Monounsaturated Fatty Acids
PPAR: Peroxisome proliferator-activated receptor
PUFA: Polyunsaturated Fatty Acids
SCHS: Singapore Chinese Health Study
SFA: Saturated Fatty Acids
SNP: Single nucleotide polymorphism
SP2: Singapore Prospective Study Programme
T2D: Type 2 diabetes
wGRS: Weighted gene-risk score
Z-BMI: Z-score transformed BMI
