# Supplementary material for: Gene-diet interaction effects on BMI levels in the Singapore Chinese population
Source: Nutr J. 2018 Feb 24;17:31. doi: 10.1186/s12937-018-0340-3 (PMC6389173; doi:10.1186/s12937-018-0340-3)
Supplement: Supplementary file 1 — Table S1. SNP information and Meta-analysis between 78 SNPs and BMI level. Table S2. Interaction between SNPs and AHEI-2010 dietary score on BMI. Table S3. Interaction between SNPs and total calories on BMI. Table S4. Interaction between SNPs and %protein on BMI. Table S5. Interaction between SNPs and %fat on BMI. Table S6. Interaction between SNPs and %SFA on BMI. Table S7. Interaction between SNPs and %MUFA on BMI. Table S8. Interaction between SNPs and %PUFA on BMI. Table S9. Interaction between SNPs and %carbohydrate on BMI. Table S10. Interaction between SNPs and %starch on BMI. Table S11. Interaction between SNPs and fiber on BMI. Table S12. Interaction between SNPs and cholesterol on BMI. Table S13. Interaction between GRS and dietary factors on BMI in individual datasets used in the study. (PDF 1521 kb) [file 12937_2018_340_MOESM1_ESM.pdf]

S1 Table: SNP information and Meta-analysis between 78 SNPs and BMI level.

				Meta-analysis of all datasets N = 7817				
SNP	Chromosome	Position	Implicated gene(s)	risk/non-risk allele	Beta	SE	P	$Q_{p\text{-value}}$
rs977747	1	47684677	*TAL1*	T/G	0.036	0.041	0.376	0.501
rs11583200	1	50559820	*ELAVL4*	C/T	-0.013	0.029	0.663	0.081
rs3101336	1	72751185	*MNEGR1*	C/T	0.040	0.029	0.175	0.165
rs12566985	1	75002193	*FPGT*	G/A	0.040	0.021	0.057	0.608
rs11165643	1	96924097	*PTBP2*	T/C	0.003	0.020	0.879	0.070
rs543874	1	1.78E+08	*SEC16B*	G/A	-0.008	0.023	0.732	0.782
rs2820292	1	2.02E+08	*NAV1*	C/A	0.008	0.020	0.697	0.321
rs13021737	2	632348	*TMEM18*	G/A	0.070	0.031	**0.024**	0.915
rs10182181	2	25150296	*NCOA1*	G/A	0.015	0.016	0.345	0.603
rs11126666	2	26928811	*KCNK3*	A/G	0.008	0.017	0.657	**0.044**
rs1016287	2	59305625	*LINC01122*	T/C	-0.015	0.018	0.416	0.598
rs11688816	2	63053048	*EHBP1*	G/A	-0.003	0.018	0.875	0.627
rs1460676	2	1.65E+08	*FIGN*	C/T	0.021	0.016	0.196	0.251
rs1528435	2	1.82E+08	*UBE2E3*	T/C	0.012	0.017	0.491	0.798
rs17203016	2	2.08E+08	*CREB1; KLF7*	G/A	0.037	0.023	0.105	0.242
rs7599312	2	2.13E+08	*ERBB4*	G/A	0.016	0.053	0.761	0.169
rs492400	2	2.19E+08	*PLCD4; CYP27A1; USP37; TTLL4; STK36; ZNF142; RQCD1*	C/T	0.022	0.019	0.255	0.404
rs2176040	2	2.27E+08	*LOC646736; IRS1*	A/G	-0.033	0.031	0.280	0.901
rs6804842	3	25106437	*RARB*	G/A	0.030	0.017	0.071	0.593
rs2365389	3	61236462	*FHIT*	C/T	0.017	0.023	0.460	0.094
rs3849570	3	81792112	*GBE1*	A/C	0.015	0.016	0.342	0.779
rs16851483	3	1.41E+08	*RASA2*	T/G	0.014	0.019	0.467	0.636
rs1516725	3	1.86E+08	*ETV5*	C/T	0.033	0.029	0.257	0.104
rs10938397	4	45182527	*GNPDA2*	G/A	0.049	0.018	**0.006**	0.134
rs17001654	4	77129568	*NUP54; SCARB2*	G/C	-0.023	0.059	0.692	0.179
rs2112347	5	75015242	*POC5*	T/G	0.017	0.016	0.289	0.130
rs7715256	5	1.54E+08	*GALNT10*	G/T	0.070	0.042	0.095	0.339
rs205262	6	34563164	*C6orf106*	G/A	0.016	0.023	0.495	0.950
rs2033529	6	40348653	*TDRG1; LRFN2*	G/A	0.023	0.021	0.276	0.386
rs2207139	6	50845490	*TFAP2B*	G/A	0.017	0.023	0.469	0.317
rs9400239	6	1.09E+08	*FOXO3; HSS00296402*	C/T	0.019	0.017	0.270	0.924
rs9374842	6	1.2E+08	*LOC285762;*	T/C	0.043	0.028	0.122	0.982
rs13201877	6	1.38E+08	*IFNGR1; OLIG3*	G/A	-0.006	0.042	0.883	0.347
rs1167827	7	75163169	*HIP1; PMS2L3; PMS2P5; WBSCR16*	G/A	0.062	0.033	0.055	0.992
rs9641123	7	93197732	*CALCR; hsa-miR-653*	C/G	0.016	0.017	0.337	0.220
rs6465468	7	95169514	*ASB4*	T/G	-0.001	0.069	0.991	0.578
rs17405819	8	76806584	*HNF4G*	T/C	-0.001	0.016	0.972	**0.022**
rs2033732	8	85079709	*RALYL*	C/T	0.036	0.016	**0.024**	0.727
rs4740619	9	15634326	*C9orf93*	T/C	0.027	0.019	0.144	0.119

rs10968576	9	28414339	*LINGO2*	G/A	0.022	0.021	0.297	0.601
rs6477694	9	1.12E+08	*EPB41L4B; C9orf4*	C/T	0.015	0.016	0.341	0.167
rs1928295	9	1.2E+08	*TLR4*	T/C	0.025	0.016	0.118	0.848
rs10733682	9	1.29E+08	*LMX1B*	A/G	0.022	0.019	0.248	0.858
rs17094222	10	1.02E+08	*HIF1AN*	C/T	0.026	0.018	0.145	0.250
rs11191560	10	1.05E+08	*NT5C2; CYP17A1; SFXN2*	C/T	0.060	0.018	**0.001**	0.155
rs7903146	10	1.15E+08	*TCF7L2*	C/T	0.021	0.053	0.695	0.918
rs4256980	11	8673939	*TRIM66*	G/C	-0.011	0.016	0.498	0.364
rs11030104	11	27684517	*BDNF*	A/G	0.001	0.016	0.960	**0.031**
rs2176598	11	43864278	*HSD17B12*	T/C	0.009	0.023	0.706	0.382
rs3817334	11	47650993	*MTCH2*	T/C	0.033	0.017	0.059	0.755
rs12286929	11	1.15E+08	*CADM1*	G/A	-0.009	0.018	0.636	**0.031**
rs7138803	12	50247468	*BCDIN3D*	A/G	-0.010	0.018	0.587	0.088
rs9581854	13	28017782	*MTIF3*	T/C	0.019	0.023	0.396	0.647
rs12429545	13	54102206	*OLFM4*	A/G	0.047	0.018	**0.011**	0.340
rs1441264	13	79580919	*MIR548A2*	A/G	0.017	0.016	0.289	0.274
rs10132280	14	25928179	*STXBP6*	C/A	0.040	0.029	0.172	0.630
rs12885454	14	29736838	*PRKD1*	C/A	-0.004	0.016	0.827	0.646
rs7141420	14	79899454	*NRXN3*	T/C	0.002	0.016	0.890	0.397
rs16951275	15	68077168	*MAP2K5*	T/C	0.031	0.016	0.055	0.962
rs7164727	15	73093991	*LOC100287559; BBS4*	T/C	0.014	0.018	0.430	0.392
rs2650492	16	28333411	*SBK1; APOBR*	A/G	0.027	0.047	0.566	**0.044**
rs3888190	16	28889486	*ATXN2L*	A/C	-0.019	0.029	0.514	0.163
rs4787491	16	30015337	*MAPK3; KCTD13; INO80E; TAOK2; YPEL3; DOC2A; FAM57B*	G/A	0.005	0.016	0.758	0.200
rs9925964	16	31129895	*KAT8; ZNF646; VKORC1; ZNF668; STX1B; FBXL19*	A/G	0.026	0.025	0.306	**0.010**
rs2080454	16	49062590	*CBLN1*	C/A	0.011	0.016	0.488	0.359
rs1558902	16	53803574	*FTO*	A/T	0.053	0.024	**0.028**	0.070
rs9914578	17	2005136	*SMG6; N29617*	G/C	0.012	0.020	0.551	0.870
rs1000940	17	5283252	*RABEP1*	G/A	-0.012	0.016	0.459	0.067
rs12940622	17	78615571	*RPTOR*	G/A	0.028	0.017	0.100	0.846
rs7239883	18	40147671	*LOC284260; RIT2*	G/A	0.005	0.017	0.756	0.694
rs7243357	18	56883319	*GRP*	T/G	0.032	0.020	0.105	0.254
rs6567160	18	57829135	*MC4R*	C/T	0.064	0.021	**0.003**	0.717
rs29941	19	34309532	*KCTD15*	G/A	0.027	0.019	0.156	0.432
rs2075650	19	45395619	*TOMM40*	A/G	0.022	0.038	0.562	0.061
rs2287019	19	46202172	*QPCTL*	C/T	0.072	0.026	**0.005**	0.970
rs3810291	19	47569003	*ZC3H4*	A/G	0.043	0.018	**0.014**	0.855
rs6091540	20	51087862	*ZFP64*	C/T	-0.031	0.017	0.079	0.197
rs2836754	21	40291740	*ETS2*	C/T	0.003	0.016	0.877	0.569

Significant (p value < 0.05) associations indicated in bold. Age and gender were included in the linear regression model as covariates.

Association results are from meta-analysis of SCHS MI cases (N = 594) and control datasets (N = 1070), SP2 610 (N = 1145) and 1M datasets (N = 949), SCHS Type 2 diabetes cases (N = 2004) and control datasets (N = 2055). Qp-value: Cochran's Q.

SCHS: Singapore Chinese Health Study; SP2: Singapore Prospective Study Program; T2D: Type II diabetes.

S2 Table: Interaction between SNPs and AHEI-2010 dietary score on BMI.

	SCHS CAD cases N = 594			SCHS CAD controls N = 1070			SP2610 N = 1145			SP21m N = 949			SCHS T2D cases N = 2004			SCHS T2D controls N = 2055			SCHS + SP2610 + SP21m N = 7817				
	Beta	SE	P	Beta	SE	P	Beta	SE	P	Beta	SE	P	Beta	SE	P	Beta	SE	P	Beta	SE	P	P_{adjust}	$Q_{p\text{-}value}$
rs977747	-0.020	0.026	0.437	0.007	0.014	0.617	-0.010	0.014	0.477	0.004	0.012	0.772	-0.002	0.012	0.876	-0.005	0.011	0.621	-0.002	0.005	0.649	1.000	0.912
rs11583200	0.005	0.013	0.713	-0.004	0.011	0.744	0.002	0.009	0.861	-0.012	0.010	0.240	0.009	0.008	0.280	0.008	0.008	0.318	0.002	0.004	0.536	1.000	0.634
rs3101336	-0.017	0.016	0.269	-0.015	0.012	0.195	0.007	0.009	0.410	-0.003	0.011	0.786	0.008	0.008	0.304	0.002	0.007	0.824	0.001	0.004	0.874	1.000	0.448
rs12566985	0.005	0.010	0.617	0.002	0.008	0.794	-0.002	0.007	0.765	2.26E-04	0.008	0.977	-0.002	0.006	0.750	-0.007	0.006	0.239	-0.002	0.003	0.529	1.000	0.913
rs11165643	0.003	0.011	0.774	0.007	0.007	0.327	-0.003	0.006	0.592	-0.006	0.007	0.381	-0.004	0.005	0.414	-0.003	0.005	0.583	-0.002	0.003	0.421	1.000	0.788
rs543874	0.005	0.011	0.645	0.002	0.009	0.841	0.001	0.007	0.865	-0.006	0.008	0.434	0.007	0.006	0.218	-3.30E-05	0.006	0.995	0.002	0.003	0.572	1.000	0.835
rs2820292	-0.015	0.009	0.109	-0.003	0.008	0.735	-0.003	0.006	0.588	0.003	0.007	0.679	4.60E-04	0.005	0.932	-0.001	0.005	0.772	-0.002	0.003	0.462	1.000	0.745
rs13021737	-0.022	0.017	0.190	-0.008	0.011	0.471	0.002	0.010	0.859	0.010	0.011	0.353	0.004	0.008	0.665	0.004	0.008	0.613	0.001	0.004	0.767	1.000	0.625
rs10182181	-2.50E-04	0.008	0.975	-0.002	0.006	0.715	-0.002	0.005	0.666	0.001	0.005	0.828	-0.003	0.004	0.546	-0.006	0.004	0.171	-0.003	0.002	0.225	1.000	0.951
rs11126666	-0.013	0.008	0.106	0.001	0.006	0.903	0.003	0.006	0.543	0.001	0.006	0.842	0.005	0.004	0.302	-0.006	0.005	0.198	-4.20E-04	0.002	0.850	1.000	0.333
rs1016287	0.002	0.009	0.859	-0.005	0.006	0.429	-1.30E-04	0.006	0.984	0.003	0.006	0.592	-0.002	0.005	0.699	0.002	0.005	0.723	-0.0002	0.002	0.935	1.000	0.944
rs11688816	-0.008	0.008	0.341	-0.007	0.006	0.289	0.003	0.005	0.518	0.001	0.006	0.811	-0.006	0.005	0.236	0.004	0.005	0.365	-0.001	0.002	0.697	1.000	0.469
rs1460676	0.004	0.008	0.646	0.002	0.006	0.687	-0.002	0.005	0.695	0.005	0.006	0.416	0.002	0.004	0.640	-0.003	0.004	0.470	0.001	0.002	0.786	1.000	0.869
rs1528435	-8.84E-07	0.009	1.000	3.86E-04	0.006	0.949	-3.10E-04	0.006	0.955	0.005	0.006	0.432	0.006	0.005	0.205	-0.007	0.004	0.091	2.53E-05	0.002	0.991	1.000	0.405
rs17203016	0.001	0.012	0.959	-0.009	0.008	0.305	0.002	0.007	0.784	-0.005	0.008	0.527	-0.005	0.006	0.377	0.002	0.006	0.718	-0.002	0.003	0.433	1.000	0.872
rs7599312	0.033	0.022	0.142	0.009	0.022	0.673	-0.002	0.015	0.904	0.003	0.021	0.892	0.014	0.015	0.375	0.014	0.012	0.259	0.011	0.007	0.114	1.000	0.857
rs492400	-0.009	0.010	0.371	-0.009	0.007	0.212	-0.001	0.006	0.905	0.017	0.006	0.008	-0.003	0.005	0.626	0.005	0.005	0.387	0.001	0.003	0.576	1.000	0.070
rs2176040	-0.007	0.014	0.634	-0.006	0.011	0.574	-0.002	0.009	0.812	-0.020	0.009	0.038	0.006	0.008	0.463	0.004	0.008	0.586	-0.002	0.004	0.516	1.000	0.378
rs6804842	-0.004	0.008	0.628	0.007	0.006	0.240	-0.004	0.005	0.512	0.005	0.006	0.422	0.006	0.005	0.179	0.007	0.004	0.103	0.004	0.002	0.073	1.000	0.560
rs2365389	-0.003	0.011	0.776	0.002	0.008	0.837	-0.008	0.008	0.331	0.002	0.008	0.845	-0.005	0.006	0.452	-0.006	0.006	0.299	-0.004	0.003	0.229	1.000	0.934
rs3849570	-0.001	0.007	0.877	-0.001	0.006	0.886	-0.005	0.005	0.324	-0.005	0.006	0.329	-0.009	0.004	0.038	0.009	0.004	0.038	-0.002	0.002	0.371	1.000	0.081
rs16851483	-0.001	0.009	0.936	0.001	0.007	0.840	0.003	0.006	0.626	0.002	0.006	0.787	-0.002	0.005	0.703	-0.006	0.005	0.237	-0.001	0.002	0.692	1.000	0.884
rs1516725	0.010	0.013	0.447	-0.007	0.011	0.519	-0.009	0.009	0.285	-0.011	0.010	0.285	-0.001	0.008	0.898	-0.004	0.007	0.578	-0.005	0.004	0.227	1.000	0.828
rs10938397	-0.010	0.009	0.263	-0.001	0.007	0.929	-0.003	0.006	0.606	0.007	0.006	0.266	-0.013	0.005	0.008	0.002	0.005	0.659	-0.003	0.002	0.248	1.000	0.127
rs17001654	0.020	0.056	0.725	-0.001	0.026	0.981	0.004	0.015	0.806	-0.038	0.023	0.099	-0.002	0.014	0.896	0.005	0.016	0.746	-0.002	0.008	0.754	1.000	0.709
rs2112347	-0.001	0.008	0.892	0.013	0.006	0.029	-0.002	0.005	0.754	-0.016	0.006	0.006	-0.007	0.004	0.122	0.006	0.004	0.175	-0.001	0.002	0.642	1.000	**0.005**
rs7715256	-0.022	0.020	0.272	-0.003	0.018	0.874	-0.011	0.015	0.463	0.010	0.017	0.572	0.019	0.012	0.121	-0.009	0.010	0.392	-0.001	0.006	0.844	1.000	0.393
rs205262	2.81E-04	0.010	0.978	0.005	0.008	0.529	-0.004	0.008	0.639	-0.002	0.009	0.840	0.002	0.006	0.766	0.004	0.006	0.522	0.001	0.003	0.642	1.000	0.967
rs2033529	-0.005	0.012	0.639	0.001	0.008	0.910	0.002	0.006	0.760	0.006	0.007	0.412	0.006	0.005	0.245	-0.001	0.005	0.870	0.002	0.003	0.384	1.000	0.898
rs2207139	-0.003	0.010	0.785	-3.80E-04	0.008	0.961	-0.001	0.008	0.899	0.008	0.008	0.332	-0.007	0.006	0.239	-0.005	0.006	0.439	-0.002	0.003	0.446	1.000	0.785
rs9400239	-0.007	0.009	0.419	0.002	0.006	0.753	0.001	0.005	0.781	-0.002	0.006	0.750	0.002	0.005	0.683	0.007	0.004	0.104	0.002	0.002	0.352	1.000	0.718
rs9374842	0.013	0.015	0.415	-0.014	0.010	0.141	-0.016	0.009	0.075	0.004	0.011	0.680	0.009	0.008	0.238	-0.007	0.007	0.335	-0.003	0.004	0.365	1.000	0.175
rs13201877	0.019	0.027	0.492	-0.030	0.015	0.045	-0.020	0.014	0.141	1.81E-04	0.015	0.990	-0.003	0.012	0.828	0.002	0.011	0.882	-0.007	0.006	0.216	1.000	0.391
rs1167827	0.004	0.017	0.837	0.017	0.014	0.226	0.002	0.010	0.847	0.014	0.011	0.230	-0.017	0.009	0.052	0.002	0.009	0.799	0.001	0.004	0.870	1.000	0.235
rs9641123	-0.005	0.008	0.558	0.001	0.006	0.907	-0.008	0.006	0.163	-2.90E-04	0.006	0.962	0.007	0.005	0.152	-0.003	0.004	0.504	-0.001	0.002	0.741	1.000	0.455
rs6465468	0.038	0.060	0.527	-0.025	0.021	0.231	0.012	0.021	0.579	-0.036	0.026	0.166	9.18E-05	0.018	0.996	-0.024	0.02	0.21	-0.011	0.009	0.21	1.000	0.540
rs17405819	0.018	0.008	0.031	0.001	0.006	0.911	-0.009	0.005	0.076	0.001	0.006	0.872	0.002	0.004	0.582	0.002	0.004	0.59	0.001	0.002	0.648	1.000	0.142
rs2033732	-0.012	0.008	0.128	-0.005	0.006	0.395	-0.001	0.005	0.779	-0.002	0.006	0.745	-0.005	0.005	0.307	-0.010	0.004	0.023	-0.006	0.002	0.009	1.000	0.749
rs4740619	-0.007	0.009	0.472	-0.007	0.007	0.29	0.002	0.006	0.724	0.004	0.007	0.570	-0.001	0.005	0.824	0.008	0.005	0.128	0.001	0.002	0.664	1.000	0.513
rs10968576	-0.015	0.011	0.155	0.008	0.008	0.346	1.45E-04	0.007	0.983	-0.007	0.007	0.308	0.004	0.006	0.483	0.005	0.006	0.404	0.001	0.003	0.763	1.000	0.410
rs6477694	-0.009	0.008	0.225	-0.008	0.006	0.164	0.002	0.005	0.723	0.001	0.006	0.914	0.008	0.004	0.078	-0.005	0.004	0.199	-0.001	0.002	0.646	1.000	0.151
rs1928295	0.003	0.008	0.712	0.005	0.006	0.454	0.006	0.005	0.228	0.004	0.006	0.517	0.001	0.004	0.851	-0.008	0.004	0.055	0.001	0.002	0.795	1.000	0.285
rs10733682	-0.009	0.009	0.307	0.002	0.007	0.785	4.42E-04	0.006	0.940	-0.003	0.007	0.633	0.002	0.005	0.745	0.002	0.005	0.712	1.87E-05	0.003	0.994	1.000	0.902
rs17094222	-0.013	0.009	0.119	0.004	0.006	0.558	0.005	0.006	0.401	-0.001	0.006	0.837	0.009	0.005	0.058	-0.001	0.005	0.87	0.002	0.002	0.361	1.000	0.277
rs11191560	0.015	0.009	0.110	0.009	0.007	0.192	0.002	0.005	0.680	0.003	0.006	0.589	0.008	0.005	0.079	-0.001	0.005	0.789	0.005	0.002	0.051	1.000	0.541
rs7903146	-0.007	0.027	0.803	0.008	0.017	0.649	0.001	0.018	0.968	-0.017	0.022	0.438	0.001	0.015	0.953	0.006	0.015	0.684	0.001	0.007	0.909	1.000	0.960
rs4256980	0.006	0.008	0.439	0.003	0.006	0.586	-0.004	0.005	0.446	0.003	0.006	0.576	0.006	0.004	0.144	-0.005	0.004	0.286	0.001	0.002	0.615	1.000	0.439
rs11030104	-0.013	0.007	0.081	0.002	0.006	0.764	0.003	0.005	0.503	-0.003	0.005	0.542	0.011	0.004	0.009	-0.002	0.004	0.67	0.001	0.002	0.476	1.000	0.061
rs2176598	-0.017	0.012	0.137	-0.008	0.008	0.314	0.008	0.007	0.251	0.007	0.009	0.427	-0.004	0.007	0.495	-0.006	0.006	0.289	-0.003	0.003	0.392	1.000	0.303
rs3817334	-0.007	0.009	0.385	0.005	0.006	0.427	-0.008	0.005	0.146	-0.008	0.006	0.217	-1.20E-04	0.005	0.979	-0.002	0.005	0.613	-0.003	0.002	0.194	1.000	0.608
rs12286929	0.001	0.009	0.935	0.002	0.006	0.736	-0.012	0.006	0.044	0.012	0.006	0.039	-0.003	0.005	0.496	0.003	0.005	0.496	2.91E-04	0.002	0.901	1.000	0.095
rs7138803	-0.002	0.010	0.843	0.004	0.007	0.544	-0.001	0.006	0.866	-0.007	0.006	0.263	-0.001	0.005	0.851	-4.60E-04	0.005	0.923	-0.001	0.002	0.644	1.000	0.910
rs9581854	-0.004	0.012	0.731	-0.013	0.009	0.154	4.10E-04	0.006	0.948	0.001	0.008	0.864	-1.20E-04	0.006	0.984	0.002	0.006	0.704	-0.001	0.003	0.780	1.000	0.813
rs12429545	0.011	0.009	0.241	0.005	0.007	0.434	0.001	0.006	0.930	-0.006	0.007	0.361	-0.002	0.005	0.682	0.004	0.005	0.462	0.001	0.002	0.628	1.000	0.653
rs1441264	0.002	0.008	0.782	-0.001	0.006	0.909	0.005	0.005	0.300	-0.003	0.006	0.607	0.002	0.005	0.628	0.002	0.004	0.671	0.002	0.002	0.462	1.000	0.935
rs10132280	-0.008	0.018	0.638	-0.017	0.010	0.098	0.014	0.010	0.141	-0.014	0.010	0.139	-0.012	0.008	0.119	-0.003	0.007	0.666	-0.007	0.004	0.090	1.000	0.215
rs12885454	0.001	0.008	0.855	0.004	0.006	0.530	-0.001	0.005	0.816	0.006	0.006	0.298	-0.003	0.004	0.517	-0.004	0.004	0.404	-0.0004	0.002	0.848	1.000	0.755
rs7141420	-0.002	0.007	0.838	-0.002	0.006	0.750	-0.001	0.005	0.816	-0.005	0.005	0.351	-0.003	0.004	0.516	0.001	0.004	0.749	-0.002	0.002	0.442	1.000	0.962

rs16951275	-0.006	0.008	0.407	0.001	0.006	0.878	-0.001	0.005	0.796	-0.011	0.006	0.059	0.011	0.004	0.010	0.001	0.004	0.896	0.001	0.002	0.731	1.000	0.054
rs7164727	0.007	0.009	0.484	-0.006	0.006	0.324	3.25E-04	0.006	0.958	0.013	0.006	0.044	-0.009	0.005	0.057	0.003	0.004	0.488	7.49E-05	0.002	0.975	1.000	0.085
rs2650492	-0.015	0.026	0.560	-0.017	0.014	0.213	-0.007	0.012	0.570	-0.008	0.013	0.528	0.021	0.015	0.169	0.031	0.016	0.045	3.84E-04	0.006	0.950	1.000	0.129
rs3888190	0.004	0.014	0.745	-0.006	0.011	0.590	0.002	0.009	0.805	-0.010	0.010	0.348	0.007	0.008	0.416	0.005	0.007	0.521	0.001	0.004	0.717	1.000	0.809
rs4787491	-0.009	0.008	0.268	-0.003	0.006	0.550	0.007	0.005	0.186	-0.009	0.006	0.117	-0.005	0.004	0.292	-0.003	0.004	0.476	-0.003	0.002	0.164	1.000	0.360
rs9925964	-0.032	0.011	0.004	0.001	0.009	0.889	-0.007	0.008	0.405	-0.003	0.010	0.788	0.004	0.007	0.576	0.004	0.007	0.541	-0.002	0.003	0.523	1.000	0.100
rs2080454	0.015	0.008	0.062	0.001	0.006	0.916	0.008	0.005	0.109	-0.007	0.006	0.236	-0.005	0.004	0.289	0.003	0.004	0.406	0.001	0.002	0.520	1.000	0.114
rs1558902	0.011	0.011	0.349	-0.005	0.009	0.623	-0.013	0.008	0.090	-0.010	0.009	0.289	0.003	0.006	0.683	0.004	0.006	0.586	-0.002	0.003	0.621	1.000	0.374
rs9914578	0.004	0.009	0.670	0.003	0.007	0.672	-0.002	0.006	0.754	-0.001	0.007	0.897	-0.002	0.005	0.670	0.006	0.005	0.226	0.001	0.003	0.634	1.000	0.863
rs1000940	-0.004	0.008	0.601	0.003	0.006	0.606	0.003	0.005	0.588	-0.008	0.006	0.146	0.004	0.004	0.364	0.004	0.004	0.344	0.001	0.002	0.512	1.000	0.515
rs12940622	-0.004	0.008	0.639	-0.002	0.006	0.722	-0.002	0.005	0.711	-0.007	0.006	0.255	4.37E-05	0.005	0.993	-0.002	0.005	0.623	-0.002	0.002	0.276	1.000	0.975
rs7239883	-0.007	0.008	0.354	-0.003	0.006	0.679	0.007	0.005	0.171	-0.003	0.006	0.582	0.005	0.004	0.261	-0.003	0.004	0.454	3.09E-04	0.002	0.888	1.000	0.414
rs7243357	-0.008	0.009	0.396	-0.011	0.007	0.148	-0.008	0.007	0.217	-0.011	0.007	0.093	-4.60E-04	0.005	0.933	0.001	0.005	0.87	-0.005	0.003	0.065	1.000	0.579
rs6567160	-0.005	0.011	0.640	0.002	0.007	0.793	0.015	0.007	0.028	0.025	0.008	0.001	-0.006	0.006	0.270	-0.004	0.006	0.549	0.004	0.003	0.200	1.000	**0.007**
rs29941	0.019	0.009	0.044	0.003	0.007	0.714	0.001	0.006	0.886	-0.008	0.007	0.235	0.005	0.005	0.335	0.003	0.005	0.484	0.003	0.002	0.262	1.000	0.324
rs2075650	-0.016	0.013	0.241	0.009	0.010	0.383	0.011	0.009	0.221	-0.014	0.010	0.158	-0.032	0.021	0.116	0.008	0.018	0.676	-0.001	0.005	0.774	1.000	0.146
rs2287019	0.020	0.011	0.060	-0.009	0.007	0.171	-0.004	0.006	0.552	-0.012	0.007	0.098	0.002	0.011	0.848	3.39E-04	0.011	0.976	-0.004	0.003	0.283	1.000	0.192
rs3810291	0.005	0.009	0.566	-0.009	0.006	0.132	-0.002	0.005	0.677	0.001	0.006	0.823	-0.001	0.005	0.797	-0.010	0.005	0.037	-0.004	0.002	0.104	1.000	0.464
rs6091540	-0.004	0.009	0.678	-0.004	0.007	0.562	0.008	0.005	0.128	0.008	0.006	0.217	-0.007	0.005	0.120	-0.010	0.005	0.025	-0.003	0.002	0.278	1.000	0.059
rs2836754	3.73E-05	0.008	0.996	0.006	0.006	0.292	0.006	0.005	0.183	-0.005	0.006	0.406	-0.006	0.004	0.195	-0.001	0.004	0.798	-2.20E-04	0.002	0.918	1.000	0.379

SCHS: Singapore Chinese Health Study; SP2: Singapore Prospective Study Program; T2D: Type II diabetes.
Age and gender were included in the linear regression model as covariates,

S3 Table: Interaction between SNPs and total calories on BMI

	SCHS CAD cases N = 594			SCHS CAD controls N = 1070			SP2610 N = 1145			SP21m N = 949			SCHS T2D cases N = 2004			SCHS T2D controls N = 2055			SCHS + SP2610 + SP21m N = 7817				
	Beta	SE	P	Beta	SE	P	Beta	SE	P	Beta	SE	P	Beta	SE	P	Beta	SE	P	Beta	SE	P	P_{adjust}	$Q_{p\text{-}value}$
rs977747	-0.099	0.141	0.483	0.097	0.111	0.381	0.140	0.108	0.193	-0.033	0.097	0.735	-0.059	0.087	0.502	0.027	0.080	0.734	0.013	0.040	0.744	1.000	0.619
rs11583200	-0.012	0.097	0.905	-0.093	0.080	0.244	0.031	0.075	0.686	0.066	0.089	0.460	-0.074	0.058	0.203	-0.036	0.060	0.551	-0.030	0.030	0.315	1.000	0.692
rs3101336	0.036	0.104	0.728	-0.030	0.083	0.717	0.053	0.076	0.485	0.073	0.092	0.426	-0.123	0.060	0.041	0.051	0.058	0.379	-0.002	0.030	0.949	1.000	0.274
rs12566985	-0.052	0.081	0.524	0.055	0.056	0.326	-0.095	0.055	0.084	0.084	0.060	0.160	-0.012	0.042	0.784	-0.022	0.042	0.603	-0.008	0.021	0.715	1.000	0.256
rs11165643	0.166	0.075	0.027	-0.037	0.057	0.517	-0.035	0.051	0.496	-0.052	0.057	0.362	0.047	0.042	0.272	-0.081	0.040	0.044	-0.015	0.021	0.464	1.000	**0.045**
rs543874	0.066	0.084	0.431	-0.001	0.066	0.984	0.015	0.061	0.811	0.007	0.065	0.918	-0.008	0.045	0.857	0.021	0.047	0.656	0.011	0.023	0.637	1.000	0.983
rs2820292	-0.028	0.071	0.699	-0.100	0.059	0.092	0.063	0.054	0.244	0.135	0.057	0.018	0.058	0.041	0.159	0.023	0.039	0.549	0.033	0.020	0.106	1.000	0.084
rs13021737	-0.085	0.093	0.359	0.027	0.087	0.760	0.009	0.075	0.907	0.016	0.088	0.859	0.059	0.069	0.391	0.034	0.063	0.592	0.018	0.031	0.568	1.000	0.892
rs10182181	4.03E-04	0.056	0.994	0.006	0.045	0.885	0.005	0.044	0.902	-0.045	0.045	0.323	0.026	0.033	0.431	-0.010	0.032	0.767	-2.40E-04	0.016	0.988	1.000	0.886
rs11126666	0.073	0.062	0.236	0.005	0.047	0.922	-0.049	0.045	0.282	-0.045	0.050	0.361	0.009	0.035	0.786	-0.012	0.035	0.723	-0.007	0.018	0.684	1.000	0.632
rs1016287	0.131	0.061	0.033	-0.050	0.049	0.303	-0.027	0.052	0.604	0.030	0.052	0.570	0.016	0.036	0.652	0.010	0.037	0.785	0.012	0.019	0.520	1.000	0.297
rs11688816	-0.055	0.060	0.359	-0.025	0.047	0.605	-0.013	0.046	0.773	0.009	0.050	0.850	0.030	0.036	0.406	0.015	0.035	0.677	0.002	0.018	0.913	1.000	0.838
rs1460676	-0.030	0.062	0.630	0.020	0.047	0.674	0.013	0.042	0.765	0.075	0.047	0.111	-0.059	0.034	0.079	-0.043	0.033	0.198	-0.014	0.017	0.413	1.000	0.212
rs1528435	-0.030	0.062	0.631	0.023	0.046	0.623	0.036	0.044	0.412	0.003	0.047	0.946	-0.036	0.035	0.302	-0.017	0.034	0.625	-0.006	0.017	0.719	1.000	0.803
rs17203016	0.173	0.088	0.049	0.009	0.062	0.888	0.061	0.061	0.318	-0.028	0.062	0.650	0.029	0.044	0.514	0.029	0.050	0.558	0.033	0.023	0.158	1.000	0.566
rs7599312	-0.133	0.230	0.561	0.147	0.160	0.357	-0.008	0.125	0.951	-0.025	0.181	0.889	0.060	0.106	0.573	-0.141	0.095	0.139	-0.023	0.053	0.663	1.000	0.620
rs492400	0.071	0.071	0.317	0.034	0.054	0.527	-0.009	0.048	0.849	-0.009	0.053	0.862	-0.057	0.041	0.164	0.051	0.038	0.183	0.007	0.02	0.703	1.000	0.411
rs2176040	0.047	0.117	0.690	0.029	0.080	0.717	0.050	0.080	0.531	-0.111	0.087	0.201	-0.028	0.062	0.651	0.126	0.059	0.032	0.029	0.031	0.350	1.000	0.280
rs6804842	-0.002	0.060	0.973	0.009	0.047	0.855	-0.067	0.045	0.137	0.036	0.046	0.429	-0.043	0.033	0.202	-0.074	0.034	0.029	-0.033	0.017	0.051	1.000	0.362
rs2365389	-0.020	0.085	0.810	0.015	0.066	0.820	0.071	0.066	0.283	-0.013	0.068	0.846	-0.038	0.046	0.411	0.042	0.045	0.355	0.009	0.024	0.691	1.000	0.750
rs3849570	0.090	0.055	0.105	-0.007	0.046	0.878	0.013	0.043	0.764	-0.067	0.047	0.153	-0.066	0.031	0.033	-0.012	0.032	0.708	-0.020	0.016	0.208	1.000	0.161
rs16851483	-0.027	0.060	0.659	-0.068	0.050	0.169	0.035	0.048	0.468	0.005	0.051	0.922	-0.069	0.038	0.068	0.004	0.037	0.905	-0.022	0.019	0.244	1.000	0.465
rs1516725	0.045	0.099	0.652	0.033	0.087	0.700	0.137	0.079	0.084	0.085	0.087	0.327	0.043	0.059	0.464	-0.012	0.056	0.835	0.045	0.030	0.131	1.000	0.760
rs10938397	0.004	0.066	0.957	-0.109	0.048	0.022	-0.037	0.049	0.451	-0.053	0.054	0.323	-0.009	0.036	0.793	0.011	0.036	0.770	-0.027	0.018	0.142	1.000	0.444
rs17001654	-0.009	0.271	0.974	-0.286	0.212	0.178	0.051	0.116	0.661	-0.026	0.173	0.882	0.043	0.111	0.699	0.049	0.109	0.653	0.013	0.057	0.818	1.000	0.801
rs2112347	-0.092	0.056	0.100	-0.048	0.045	0.283	0.072	0.041	0.083	-0.013	0.045	0.767	0.010	0.033	0.766	-0.001	0.032	0.982	-0.003	0.016	0.875	1.000	0.218
rs7715256	0.169	0.160	0.292	0.066	0.121	0.585	-0.081	0.123	0.509	-0.019	0.115	0.872	-0.213	0.087	0.014	-0.025	0.082	0.758	-0.053	0.043	0.226	1.000	0.253
rs205262	-0.177	0.079	0.025	-0.043	0.060	0.474	0.106	0.060	0.077	0.095	0.066	0.151	-0.103	0.048	0.033	0.064	0.045	0.156	-0.001	0.023	0.961	1.000	**0.004**
rs2033529	0.118	0.086	0.169	0.007	0.061	0.905	-0.072	0.055	0.192	0.029	0.060	0.630	0.080	0.042	0.056	-0.075	0.046	0.106	0.007	0.022	0.766	1.000	0.074
rs2207139	0.065	0.084	0.438	0.010	0.065	0.874	0.007	0.066	0.911	-0.071	0.064	0.270	-0.063	0.044	0.157	0.043	0.046	0.353	-0.009	0.023	0.715	1.000	0.467
rs9400239	-0.023	0.062	0.706	-0.058	0.048	0.222	0.096	0.046	0.037	-0.012	0.052	0.818	-0.009	0.035	0.794	-0.031	0.034	0.364	-0.008	0.018	0.657	1.000	0.241
rs9374842	-0.043	0.115	0.712	-0.054	0.072	0.453	-0.101	0.075	0.182	0.121	0.080	0.131	-0.072	0.056	0.197	-0.030	0.052	0.568	-0.036	0.028	0.194	1.000	0.409
rs13201877	-0.287	0.198	0.148	-0.139	0.106	0.193	-0.081	0.104	0.436	0.366	0.121	0.003	-0.028	0.088	0.747	0.188	0.084	0.025	0.039	0.043	0.363	1.000	**0.003**
rs1167827	0.059	0.141	0.675	0.010	0.083	0.900	0.052	0.087	0.547	0.030	0.090	0.740	0.002	0.064	0.976	-0.066	0.067	0.325	0.001	0.033	0.974	1.000	0.897

rs9641123	0.033	0.061	0.595	-0.060	0.048	0.210	-0.031	0.046	0.498	0.030	0.048	0.527	-0.028	0.035	0.431	-0.063	0.034	0.062	-0.029	0.017	0.093	1.000	0.549
rs6465468	-0.041	0.323	0.899	-0.170	0.208	0.413	0.046	0.183	0.803	-0.050	0.225	0.824	-0.071	0.126	0.570	-0.167	0.159	0.294	-0.081	0.073	0.268	1.000	0.963
rs17405819	0.033	0.062	0.593	-0.045	0.045	0.314	-0.096	0.042	0.024	0.015	0.049	0.764	-0.032	0.032	0.312	-0.012	0.031	0.695	-0.028	0.016	0.084	1.000	0.450
rs2033732	0.066	0.057	0.248	0.026	0.045	0.565	-0.022	0.043	0.619	-0.001	0.047	0.982	-0.027	0.033	0.405	0.022	0.032	0.492	0.005	0.016	0.776	1.000	0.700
rs4740619	-0.088	0.064	0.169	-0.013	0.053	0.799	0.011	0.047	0.823	0.032	0.054	0.551	0.037	0.037	0.322	-0.122	0.039	0.002	-0.023	0.019	0.227	1.000	**0.040**
rs10968576	0.011	0.075	0.882	-0.068	0.055	0.216	0.053	0.055	0.336	-0.136	0.062	0.028	-0.089	0.043	0.039	-0.011	0.043	0.791	-0.043	0.021	0.047	1.000	0.174
rs6477694	-0.044	0.056	0.436	-0.013	0.044	0.765	0.027	0.042	0.518	0.007	0.045	0.869	-0.040	0.032	0.216	-0.054	0.031	0.085	-0.025	0.016	0.126	1.000	0.654
rs1928295	0.039	0.059	0.515	-0.097	0.045	0.033	0.020	0.041	0.630	-0.008	0.048	0.870	-0.019	0.033	0.560	0.017	0.033	0.614	-0.008	0.017	0.616	1.000	0.348
rs10733682	0.085	0.068	0.213	-0.081	0.053	0.125	0.003	0.047	0.942	-0.039	0.056	0.484	-0.042	0.039	0.277	-0.019	0.039	0.629	-0.023	0.019	0.228	1.000	0.497
rs17094222	0.023	0.063	0.721	-0.005	0.047	0.915	-0.016	0.046	0.728	0.055	0.053	0.299	-0.024	0.038	0.521	-0.007	0.036	0.842	-0.002	0.018	0.896	1.000	0.879
rs11191560	0.056	0.067	0.402	-0.047	0.052	0.370	-0.018	0.046	0.693	-0.092	0.054	0.085	0.022	0.036	0.544	0.005	0.036	0.879	-0.008	0.018	0.659	1.000	0.437
rs7903146	-0.177	0.207	0.394	-0.151	0.139	0.279	0.055	0.146	0.707	-0.323	0.189	0.088	0.255	0.103	0.013	0.110	0.103	0.287	0.048	0.054	0.380	1.000	**0.043**
rs4256980	0.009	0.060	0.882	0.039	0.044	0.373	0.035	0.042	0.404	0.001	0.047	0.979	-0.020	0.032	0.528	0.054	0.033	0.102	0.020	0.016	0.231	1.000	0.676
rs11030104	0.033	0.057	0.561	0.025	0.044	0.566	0.015	0.041	0.720	0.032	0.045	0.479	0.003	0.032	0.915	0.039	0.032	0.213	0.023	0.016	0.146	1.000	0.980
rs2176598	-0.096	0.080	0.230	-0.018	0.062	0.768	0.001	0.064	0.988	0.031	0.067	0.643	0.001	0.047	0.988	-0.086	0.046	0.065	-0.029	0.024	0.218	1.000	0.603
rs3817334	-0.008	0.061	0.901	-0.016	0.049	0.739	-0.003	0.047	0.944	-0.060	0.050	0.226	0.012	0.035	0.727	0.025	0.034	0.476	-0.001	0.018	0.942	1.000	0.817
rs12286929	0.042	0.064	0.511	-0.042	0.048	0.384	-0.124	0.049	0.011	-0.034	0.052	0.516	0.029	0.036	0.425	0.024	0.037	0.520	-0.011	0.018	0.554	1.000	0.120
rs7138803	0.047	0.072	0.512	0.010	0.050	0.847	-0.040	0.047	0.395	0.091	0.055	0.103	-0.011	0.035	0.749	-0.002	0.035	0.964	0.005	0.018	0.799	1.000	0.564
rs9581854	-0.103	0.078	0.187	0.023	0.061	0.703	-0.102	0.060	0.089	0.005	0.062	0.936	0.058	0.047	0.218	0.032	0.045	0.468	0.002	0.023	0.915	1.000	0.233
rs12429545	-0.009	0.071	0.904	0.034	0.048	0.480	-0.039	0.046	0.404	0.046	0.055	0.402	-0.007	0.038	0.848	-0.025	0.037	0.513	-0.004	0.019	0.819	1.000	0.802
rs1441264	-0.036	0.058	0.531	0.021	0.045	0.641	0.057	0.042	0.176	-0.020	0.047	0.665	0.040	0.034	0.235	0.029	0.033	0.380	0.023	0.017	0.162	1.000	0.721
rs10132280	-0.072	0.108	0.503	-0.041	0.077	0.599	0.061	0.085	0.476	-0.081	0.088	0.361	0.022	0.061	0.725	-0.026	0.058	0.648	-0.016	0.030	0.603	1.000	0.831
rs12885454	0.011	0.058	0.852	-0.015	0.045	0.740	0.045	0.041	0.268	-0.017	0.049	0.726	0.021	0.033	0.528	-0.010	0.032	0.754	0.007	0.016	0.674	1.000	0.874
rs7141420	-0.076	0.056	0.179	0.004	0.045	0.927	-0.104	0.042	0.014	-0.054	0.047	0.254	-0.044	0.033	0.179	0.017	0.032	0.601	-0.035	0.016	0.034	1.000	0.235
rs16951275	-0.005	0.062	0.941	-0.007	0.043	0.876	-0.029	0.043	0.495	0.011	0.047	0.810	-0.008	0.033	0.804	0.025	0.033	0.446	-5.12E-06	0.017	1.000	1.000	0.945
rs7164727	-0.041	0.068	0.550	-0.040	0.050	0.428	0.023	0.049	0.641	0.039	0.054	0.463	-0.051	0.037	0.172	-0.003	0.035	0.929	-0.014	0.018	0.452	1.000	0.693
rs2650492	0.175	0.174	0.315	-0.059	0.092	0.517	-0.086	0.106	0.417	0.046	0.100	0.642	0.267	0.137	0.052	0.111	0.130	0.392	0.036	0.047	0.442	1.000	0.285
rs3888190	0.124	0.114	0.276	0.029	0.081	0.716	0.067	0.076	0.379	-0.046	0.079	0.556	0.089	0.062	0.152	0.054	0.058	0.352	0.051	0.030	0.087	1.000	0.787
rs4787491	0.009	0.060	0.879	-0.052	0.043	0.228	0.044	0.041	0.276	-0.042	0.045	0.348	0.029	0.033	0.378	-0.077	0.032	0.016	-0.018	0.016	0.271	1.000	0.114
rs9925964	-0.073	0.088	0.405	0.019	0.071	0.787	-0.006	0.069	0.934	-0.011	0.075	0.880	-8.07E-07	0.052	1.000	0.069	0.049	0.157	0.013	0.026	0.605	1.000	0.771
rs2080454	0.024	0.060	0.683	0.033	0.044	0.455	-0.001	0.041	0.981	-0.040	0.044	0.359	0.065	0.031	0.037	0.040	0.031	0.201	0.028	0.016	0.078	1.000	0.483
rs1558902	-0.051	0.089	0.569	1.45E-04	0.070	0.998	-0.050	0.067	0.453	0.110	0.077	0.154	-0.028	0.048	0.561	-0.033	0.050	0.507	-0.016	0.025	0.533	1.000	0.652
rs9914578	0.064	0.066	0.329	-0.013	0.054	0.805	-0.096	0.053	0.073	-0.030	0.060	0.614	-0.012	0.038	0.752	0.017	0.040	0.682	-0.012	0.020	0.549	1.000	0.493
rs1000940	0.091	0.064	0.153	0.044	0.045	0.329	0.040	0.042	0.342	-0.013	0.049	0.786	0.031	0.032	0.333	-0.001	0.033	0.981	0.025	0.017	0.131	1.000	0.755
rs12940622	0.058	0.060	0.338	-0.016	0.047	0.728	0.015	0.045	0.743	-0.075	0.051	0.142	-0.043	0.035	0.220	0.027	0.034	0.436	-0.008	0.017	0.661	1.000	0.389
rs7239883	-0.008	0.061	0.895	0.032	0.047	0.503	-0.021	0.045	0.633	0.029	0.049	0.555	0.008	0.034	0.818	-0.010	0.033	0.758	0.003	0.017	0.851	1.000	0.949
rs7243357	-0.008	0.070	0.910	0.050	0.052	0.331	-0.037	0.057	0.515	0.056	0.056	0.318	-0.042	0.042	0.309	0.037	0.038	0.330	0.010	0.020	0.616	1.000	0.533
rs6567160	-0.024	0.071	0.740	0.080	0.059	0.171	-0.010	0.055	0.854	0.077	0.063	0.224	-0.002	0.042	0.961	-0.032	0.044	0.459	0.008	0.022	0.721	1.000	0.560
rs29941	-0.008	0.072	0.912	0.065	0.054	0.230	0.080	0.051	0.117	-0.152	0.060	0.011	-0.026	0.038	0.495	-0.009	0.037	0.818	-0.006	0.020	0.757	1.000	0.054
rs2075650	0.155	0.106	0.146	0.020	0.075	0.790	-0.049	0.073	0.507	-0.061	0.078	0.431	0.120	0.154	0.437	-0.034	0.151	0.823	0.002	0.038	0.949	1.000	0.564
rs2287019	0.138	0.067	0.040	0.024	0.056	0.670	0.052	0.056	0.349	-0.060	0.059	0.308	-0.017	0.089	0.847	0.030	0.085	0.722	0.028	0.027	0.291	1.000	0.369
rs3810291	-0.022	0.063	0.722	-0.045	0.048	0.352	-0.070	0.045	0.124	0.031	0.052	0.548	0.016	0.035	0.660	-0.016	0.036	0.668	-0.015	0.018	0.400	1.000	0.639
rs6091540	0.012	0.063	0.854	-0.004	0.047	0.924	-0.014	0.046	0.761	0.080	0.051	0.118	0.010	0.034	0.773	-0.033	0.035	0.338	0.002	0.018	0.919	1.000	0.612
rs2836754	0.017	0.061	0.778	-0.007	0.043	0.871	0.040	0.041	0.337	-0.036	0.049	0.466	-0.026	0.033	0.417	-0.016	0.033	0.618	-0.008	0.017	0.609	1.000	0.820

SCHS: Singapore Chinese Health Study; SP2: Singapore Prospective Study Program; T2D: Type II diabetes.

Age and gender were included in the linear regression model as covariates.

S4 Table: Interaction between SNPs and %protein on BMI

	SCHS CAD cases N = 594			SCHS CAD controls N = 1070			SP2610 N = 1145			SP21m N = 949			SCHS T2D cases N = 2004			SCHS T2D controls N = 2055			SCHS + SP2610 + SP21m N = 7817				
	Beta	SE	P	Beta	SE	P	Beta	SE	P	Beta	SE	P	Beta	SE	P	Beta	SE	P	Beta	SE	P	P_{adjust}	$Q_{p\text{-}value}$
rs977747	-0.030	0.177	0.866	0.276	0.111	0.014	-0.195	0.122	0.111	-0.124	0.104	0.233	0.046	0.088	0.600	-0.142	0.075	0.056	-0.039	0.041	0.343	1.000	**0.022**
rs11583200	0.078	0.084	0.353	-0.028	0.073	0.706	0.096	0.076	0.208	0.028	0.096	0.770	0.079	0.059	0.180	0.082	0.059	0.164	0.061	0.029	0.037	1.000	0.842
rs3101336	-0.079	0.113	0.484	-0.190	0.080	0.017	-0.079	0.069	0.251	-0.027	0.082	0.739	-0.007	0.062	0.915	-0.101	0.054	0.061	-0.078	0.029	0.007	1.000	0.569
rs12566985	0.008	0.078	0.917	0.089	0.056	0.111	-0.021	0.052	0.686	-0.022	0.061	0.720	-0.004	0.041	0.916	-0.030	0.043	0.494	-0.001	0.021	0.962	1.000	0.651
rs11165643	0.137	0.076	0.074	-0.001	0.058	0.983	0.069	0.054	0.202	-0.010	0.055	0.859	-0.022	0.042	0.597	-0.068	0.040	0.087	-0.005	0.021	0.801	1.000	0.155
rs543874	0.114	0.085	0.181	0.078	0.062	0.210	-0.021	0.061	0.734	0.039	0.066	0.556	-0.006	0.046	0.896	0.014	0.043	0.737	0.023	0.023	0.312	1.000	0.710
rs2820292	0.060	0.066	0.363	-0.092	0.055	0.094	-0.037	0.053	0.492	0.009	0.057	0.875	0.001	0.041	0.981	-0.053	0.037	0.161	-0.025	0.020	0.204	1.000	0.480
rs13021737	-0.104	0.119	0.384	-0.138	0.086	0.107	-0.027	0.075	0.719	-0.032	0.090	0.726	0.030	0.065	0.648	0.054	0.067	0.422	-0.016	0.032	0.613	1.000	0.520
rs10182181	0.080	0.056	0.154	0.032	0.043	0.463	-0.077	0.043	0.073	-0.009	0.046	0.849	-0.014	0.033	0.663	-0.032	0.031	0.304	-0.013	0.016	0.426	1.000	0.266
rs11126666	-0.118	0.061	0.052	0.003	0.049	0.958	-0.029	0.047	0.544	-0.071	0.048	0.144	0.026	0.034	0.440	0.049	0.034	0.154	-0.003	0.017	0.863	1.000	0.113
rs1016287	0.102	0.065	0.114	-0.027	0.050	0.586	-0.078	0.049	0.115	0.067	0.052	0.199	0.025	0.037	0.495	-0.022	0.037	0.560	0.003	0.019	0.866	1.000	0.173
rs11688816	-0.003	0.063	0.958	-0.122	0.045	0.007	0.029	0.045	0.517	0.007	0.050	0.891	-0.007	0.037	0.850	-0.047	0.035	0.176	-0.027	0.018	0.123	1.000	0.209
rs1460676	-0.017	0.061	0.781	-0.012	0.042	0.783	0.050	0.042	0.235	-0.019	0.046	0.686	-0.005	0.034	0.877	0.050	0.032	0.124	0.014	0.016	0.398	1.000	0.636
rs1528435	0.018	0.066	0.788	-0.101	0.045	0.025	0.004	0.044	0.921	0.006	0.049	0.901	0.024	0.034	0.477	-0.031	0.033	0.335	-0.014	0.017	0.401	1.000	0.323
rs17203016	0.044	0.089	0.625	-0.031	0.065	0.628	0.005	0.057	0.935	0.057	0.066	0.390	-0.023	0.045	0.612	0.052	0.048	0.276	0.013	0.023	0.575	1.000	0.799
rs7599312	0.257	0.194	0.186	-0.045	0.171	0.794	-0.091	0.136	0.503	-0.053	0.144	0.711	-0.058	0.100	0.562	0.108	0.094	0.253	0.011	0.052	0.832	1.000	0.549
rs492400	0.061	0.078	0.436	0.026	0.055	0.643	0.005	0.052	0.930	0.024	0.055	0.655	-0.038	0.039	0.328	0.026	0.038	0.505	0.008	0.020	0.681	1.000	0.812
rs2176040	-0.117	0.111	0.293	-0.140	0.089	0.117	-0.044	0.087	0.614	0.006	0.087	0.944	-0.013	0.063	0.841	0.044	0.061	0.469	-0.024	0.032	0.458	1.000	0.572
rs6804842	-0.034	0.062	0.586	0.046	0.047	0.335	0.070	0.046	0.129	-0.048	0.047	0.306	-0.044	0.034	0.197	0.009	0.034	0.788	-0.002	0.017	0.902	1.000	0.278
rs2365389	0.027	0.082	0.744	-0.037	0.063	0.560	-0.045	0.065	0.489	-0.011	0.071	0.879	0.032	0.048	0.510	-0.011	0.048	0.820	-0.005	0.024	0.825	1.000	0.926
rs3849570	-0.019	0.058	0.740	-0.003	0.043	0.936	0.006	0.039	0.880	-0.037	0.049	0.459	-0.010	0.032	0.760	-0.018	0.031	0.557	-0.012	0.016	0.452	1.000	0.990
rs16851483	-0.034	0.067	0.607	-0.035	0.052	0.504	0.052	0.045	0.248	-0.069	0.053	0.196	-0.054	0.038	0.157	-0.066	0.038	0.077	-0.037	0.019	0.052	1.000	0.407
rs1516725	-0.066	0.116	0.572	-0.051	0.087	0.554	0.121	0.070	0.085	-0.040	0.094	0.670	0.052	0.059	0.379	-0.074	0.056	0.188	5.94E-05	0.030	0.998	1.000	0.275
rs10938397	0.112	0.069	0.107	-0.021	0.051	0.677	-0.098	0.048	0.044	0.048	0.052	0.358	-0.051	0.036	0.161	0.089	0.036	0.014	0.007	0.019	0.710	1.000	**0.008**
rs17001654	-0.025	0.211	0.906	-0.008	0.196	0.969	0.391	0.149	0.009	0.138	0.171	0.418	-0.015	0.111	0.894	0.068	0.102	0.507	0.089	0.057	0.122	1.000	0.339
rs2112347	0.039	0.057	0.491	0.011	0.044	0.798	-0.018	0.044	0.687	-0.055	0.047	0.241	-0.071	0.032	0.027	-0.029	0.032	0.363	-0.030	0.016	0.063	1.000	0.500
rs7715256	0.208	0.149	0.163	0.070	0.144	0.624	-0.046	0.104	0.656	-0.116	0.127	0.361	0.021	0.087	0.808	-0.146	0.080	0.066	-0.035	0.043	0.412	1.000	0.307
rs205262	0.018	0.079	0.822	0.203	0.063	0.001	-0.002	0.063	0.971	-0.046	0.070	0.514	0.021	0.047	0.660	-0.010	0.044	0.817	0.026	0.023	0.261	1.000	0.082
rs2033529	-0.069	0.081	0.399	-0.091	0.062	0.140	0.054	0.053	0.310	0.006	0.063	0.921	-0.053	0.043	0.217	0.054	0.045	0.228	-0.007	0.022	0.736	1.000	0.236
rs2207139	-0.008	0.077	0.918	-0.046	0.063	0.464	-0.001	0.064	0.982	0.005	0.066	0.939	0.026	0.046	0.568	0.009	0.048	0.852	0.002	0.024	0.921	1.000	0.970
rs9400239	0.098	0.064	0.126	-0.025	0.048	0.600	0.014	0.045	0.756	-0.009	0.050	0.856	0.020	0.034	0.549	-0.007	0.034	0.837	0.009	0.017	0.625	1.000	0.712
rs9374842	-0.141	0.118	0.233	-0.017	0.079	0.829	-0.037	0.078	0.630	-0.137	0.083	0.098	-0.010	0.057	0.859	0.052	0.056	0.352	-0.022	0.029	0.458	1.000	0.443
rs13201877	0.053	0.211	0.802	-0.131	0.093	0.158	0.144	0.105	0.172	-0.105	0.127	0.409	0.080	0.083	0.334	0.115	0.076	0.130	0.039	0.040	0.331	1.000	0.229
rs1167827	0.053	0.143	0.713	0.015	0.094	0.869	0.170	0.092	0.064	-0.053	0.091	0.563	-0.035	0.064	0.589	0.084	0.064	0.190	0.034	0.034	0.310	1.000	0.426
rs9641123	-0.038	0.060	0.524	0.052	0.047	0.266	-0.004	0.045	0.934	-0.001	0.050	0.976	0.012	0.035	0.742	0.013	0.035	0.704	0.010	0.017	0.587	1.000	0.900
rs6465468	-0.397	0.563	0.481	0.055	0.167	0.743	-0.134	0.189	0.479	0.036	0.209	0.863	-0.052	0.131	0.691	0.121	0.144	0.404	0.003	0.071	0.969	1.000	0.851
rs17405819	-0.046	0.063	0.461	-0.041	0.044	0.351	0.007	0.042	0.872	0.030	0.047	0.529	-0.036	0.031	0.250	0.014	0.031	0.646	-0.010	0.016	0.543	1.000	0.700
rs2033732	-0.034	0.058	0.552	-0.005	0.045	0.919	-0.001	0.042	0.982	-0.114	0.045	0.011	-0.016	0.034	0.626	0.033	0.032	0.297	-0.014	0.016	0.394	1.000	0.188
rs4740619	0.016	0.064	0.796	0.039	0.052	0.454	0.032	0.048	0.502	0.062	0.055	0.264	0.018	0.038	0.637	0.122	0.038	0.001	0.054	0.019	0.004	1.000	0.437
rs10968576	-0.055	0.075	0.469	-0.006	0.058	0.912	-0.041	0.057	0.468	-0.118	0.058	0.043	0.052	0.043	0.228	0.022	0.043	0.612	-0.009	0.021	0.685	1.000	0.244
rs6477694	-0.067	0.058	0.247	-0.029	0.045	0.520	-0.049	0.043	0.254	-0.019	0.044	0.660	-0.042	0.033	0.202	-0.056	0.032	0.079	-0.044	0.016	0.007	1.000	0.980
rs1928295	-0.021	0.058	0.717	0.001	0.043	0.986	0.071	0.041	0.085	-0.035	0.047	0.464	-0.040	0.033	0.220	0.004	0.032	0.900	-0.003	0.016	0.833	1.000	0.401
rs10733682	-0.057	0.066	0.388	-0.026	0.053	0.631	-0.013	0.047	0.787	0.021	0.054	0.698	-0.024	0.038	0.536	0.009	0.039	0.822	-0.012	0.019	0.550	1.000	0.937
rs17094222	-0.019	0.062	0.766	-0.036	0.047	0.444	0.038	0.046	0.410	-0.001	0.054	0.988	-0.003	0.037	0.940	0.096	0.038	0.011	0.021	0.018	0.256	1.000	0.259
rs11191560	-0.015	0.063	0.811	-0.021	0.048	0.669	0.048	0.045	0.280	-0.070	0.052	0.183	-0.054	0.035	0.122	0.011	0.034	0.742	-0.015	0.018	0.391	1.000	0.423
rs7903146	-0.275	0.226	0.225	0.069	0.150	0.645	0.183	0.182	0.314	0.115	0.145	0.430	0.153	0.100	0.125	0.140	0.124	0.261	0.108	0.057	0.059	1.000	0.644
rs4256980	0.077	0.065	0.239	0.060	0.045	0.181	-0.069	0.041	0.094	-0.073	0.046	0.112	-0.007	0.032	0.822	0.030	0.033	0.366	-0.002	0.017	0.909	1.000	0.095
rs11030104	0.032	0.059	0.590	-0.006	0.042	0.887	0.087	0.041	0.036	0.005	0.047	0.907	0.055	0.032	0.085	0.013	0.032	0.689	0.033	0.016	0.043	1.000	0.583
rs2176598	-0.003	0.087	0.973	0.025	0.061	0.679	-0.052	0.060	0.381	-0.033	0.069	0.628	0.063	0.047	0.180	-0.034	0.045	0.449	-0.002	0.023	0.932	1.000	0.617
rs3817334	0.023	0.060	0.707	-0.032	0.050	0.523	-0.019	0.047	0.681	-0.044	0.051	0.386	-0.016	0.035	0.640	0.007	0.034	0.829	-0.012	0.018	0.487	1.000	0.940
rs12286929	0.063	0.067	0.347	0.017	0.050	0.726	0.053	0.047	0.262	-0.031	0.052	0.558	0.005	0.036	0.885	-0.023	0.036	0.522	0.007	0.018	0.715	1.000	0.714
rs7138803	0.035	0.066	0.595	0.009	0.050	0.850	-0.037	0.049	0.453	0.072	0.054	0.183	-0.003	0.036	0.937	-0.014	0.037	0.710	0.003	0.019	0.872	1.000	0.734
rs9581854	-0.058	0.084	0.490	-0.122	0.070	0.080	-0.068	0.057	0.236	-0.072	0.067	0.281	0.034	0.046	0.453	0.051	0.044	0.244	-0.015	0.023	0.530	1.000	0.177
rs12429545	-0.017	0.069	0.811	0.055	0.048	0.251	-0.008	0.048	0.863	0.023	0.054	0.673	0.040	0.037	0.273	0.042	0.037	0.249	0.030	0.019	0.110	1.000	0.905
rs1441264	-0.013	0.059	0.829	-0.067	0.047	0.153	0.067	0.041	0.102	-0.013	0.046	0.778	-0.016	0.033	0.623	-0.007	0.033	0.834	-0.006	0.017	0.724	1.000	0.416
rs10132280	-0.008	0.106	0.938	-0.090	0.076	0.236	-0.208	0.081	0.010	-0.045	0.094	0.635	0.053	0.061	0.389	0.018	0.056	0.752	-0.030	0.030	0.310	1.000	0.150
rs12885454	0.076	0.057	0.179	-1.18E-04	0.045	0.998	0.002	0.041	0.964	-0.009	0.048	0.846	0.044	0.033	0.174	0.039	0.032	0.220	0.027	0.016	0.100	1.000	0.776
rs7141420	0.061	0.058	0.299	-0.105	0.043	0.015	-0.002	0.043	0.955	0.028	0.043	0.516	0.047	0.033	0.154	-0.020	0.031	0.528	-3.90E-04	0.016	0.981	1.000	0.079

rs16951275	0.003	0.059	0.964	-0.033	0.043	0.444	0.024	0.044	0.591	-2.04E-04	0.047	0.997	0.016	0.033	0.632	-0.055	0.032	0.090	-0.012	0.017	0.479	1.000	0.626
rs7164727	-0.012	0.064	0.851	-0.009	0.052	0.861	-0.025	0.051	0.627	0.080	0.051	0.119	0.042	0.037	0.252	0.041	0.035	0.248	0.027	0.018	0.146	1.000	0.659
rs2650492	0.052	0.159	0.744	-0.040	0.101	0.695	0.015	0.097	0.880	0.029	0.105	0.784	0.097	0.129	0.452	0.323	0.124	0.009	0.064	0.047	0.171	1.000	0.318
rs3888190	-0.094	0.109	0.388	0.040	0.083	0.628	0.091	0.074	0.221	0.043	0.079	0.585	0.062	0.058	0.290	-0.022	0.058	0.700	0.028	0.029	0.334	1.000	0.682
rs4787491	-0.122	0.062	0.049	0.002	0.043	0.966	0.063	0.042	0.131	-0.096	0.047	0.040	-0.051	0.033	0.122	-0.027	0.032	0.405	-0.030	0.016	0.067	1.000	0.071
rs9925964	-0.075	0.088	0.390	-0.059	0.067	0.373	0.041	0.066	0.537	0.085	0.077	0.271	-0.036	0.051	0.476	0.055	0.051	0.278	0.005	0.026	0.841	1.000	0.444
rs2080454	0.088	0.060	0.142	-0.016	0.044	0.714	-0.020	0.042	0.634	0.041	0.045	0.367	0.030	0.032	0.347	0.059	0.031	0.060	0.030	0.016	0.066	1.000	0.498
rs1558902	0.005	0.082	0.948	0.078	0.062	0.207	0.082	0.067	0.222	-0.141	0.070	0.044	0.044	0.047	0.348	0.053	0.049	0.275	0.031	0.024	0.199	1.000	0.181
rs9914578	0.064	0.073	0.377	0.028	0.054	0.607	0.026	0.053	0.622	-0.074	0.060	0.216	0.072	0.039	0.064	0.026	0.037	0.494	0.030	0.020	0.130	1.000	0.489
rs1000940	-0.155	0.060	0.009	-0.109	0.046	0.019	0.005	0.041	0.901	-0.016	0.046	0.736	0.011	0.033	0.730	-0.009	0.033	0.777	-0.026	0.017	0.111	1.000	0.073
rs12940622	-0.066	0.060	0.273	-0.006	0.048	0.898	-0.003	0.046	0.944	0.035	0.049	0.480	-0.019	0.035	0.573	0.009	0.034	0.799	-0.005	0.017	0.767	1.000	0.847
rs7239883	-0.050	0.064	0.434	-0.067	0.045	0.142	0.007	0.042	0.864	-0.088	0.052	0.088	-0.011	0.034	0.754	0.033	0.035	0.347	-0.017	0.017	0.337	1.000	0.328
rs7243357	-0.139	0.071	0.050	-1.70E-04	0.052	0.997	-0.011	0.056	0.846	0.005	0.057	0.934	0.014	0.040	0.729	-0.023	0.039	0.559	-0.014	0.020	0.469	1.000	0.574
rs6567160	0.102	0.079	0.197	0.068	0.059	0.246	0.080	0.056	0.155	-0.015	0.061	0.809	-0.075	0.042	0.071	-0.006	0.045	0.888	0.005	0.022	0.810	1.000	0.139
rs29941	0.077	0.076	0.316	-0.014	0.053	0.790	-0.058	0.052	0.264	-0.048	0.055	0.387	0.041	0.039	0.295	-0.010	0.037	0.775	-0.004	0.019	0.851	1.000	0.518
rs2075650	0.098	0.110	0.376	-0.001	0.082	0.986	-0.048	0.075	0.518	-0.035	0.084	0.675	-0.185	0.153	0.226	0.048	0.130	0.714	-0.017	0.039	0.669	1.000	0.731
rs2287019	0.021	0.076	0.779	0.118	0.057	0.037	-0.059	0.053	0.266	-0.027	0.062	0.661	-0.063	0.087	0.471	-0.052	0.088	0.553	-0.002	0.027	0.942	1.000	0.242
rs3810291	-0.021	0.066	0.747	0.036	0.049	0.461	-0.004	0.044	0.929	0.008	0.050	0.878	-0.039	0.035	0.269	-0.056	0.036	0.121	-0.020	0.018	0.262	1.000	0.692
rs6091540	-0.005	0.065	0.936	0.070	0.049	0.157	0.028	0.047	0.549	0.044	0.049	0.375	-0.007	0.036	0.840	-0.021	0.035	0.552	0.011	0.018	0.525	1.000	0.677
rs2836754	-0.062	0.059	0.296	-0.024	0.047	0.602	-0.009	0.043	0.835	-0.065	0.048	0.180	-0.055	0.033	0.094	-0.007	0.033	0.836	-0.033	0.017	0.047	1.000	0.832

SCHS: Singapore Chinese Health Study; SP2: Singapore Prospective Study Program; T2D: Type II diabetes.
Age gender and total calories intake were included in the linear regression model as covariates.

S5 Table: Interaction between SNPs and %fat on BMI

	SCHS CAD cases N = 594			SCHS CAD controls N = 1070			SP2610 N = 1145			SP21m N = 949			SCHS T2D cases N = 2004			SCHS T2D controls N = 2055			SCHS + SP2610 + SP21m N = 7817				
	Beta	SE	P	Beta	SE	P	Beta	SE	P	Beta	SE	P	Beta	SE	P	Beta	SE	P	Beta	SE	P	P_{adjust}	$Q_{p\text{-}value}$
rs977747	-0.056	0.176	0.753	0.200	0.115	0.083	0.086	0.117	0.461	-0.097	0.103	0.349	-0.097	0.087	0.264	-0.079	0.078	0.311	-0.027	0.042	0.521	1.000	0.269
rs11583200	0.122	0.091	0.182	0.010	0.078	0.896	-0.018	0.080	0.826	0.082	0.089	0.357	0.045	0.062	0.467	0.044	0.058	0.448	0.043	0.030	0.146	1.000	0.890
rs3101336	-0.085	0.106	0.421	-0.175	0.086	0.042	-0.014	0.069	0.843	0.076	0.097	0.432	0.020	0.061	0.742	-0.057	0.056	0.307	-0.034	0.030	0.247	1.000	0.384
rs12566985	-0.006	0.073	0.931	0.104	0.056	0.065	-0.034	0.056	0.546	0.024	0.059	0.688	0.002	0.041	0.957	-0.011	0.043	0.793	0.010	0.021	0.629	1.000	0.580
rs11165643	0.115	0.076	0.130	-0.005	0.057	0.924	0.077	0.052	0.142	0.047	0.057	0.404	-0.024	0.041	0.560	-0.028	0.041	0.495	0.013	0.021	0.532	1.000	0.349
rs543874	0.094	0.088	0.285	0.068	0.060	0.254	-0.002	0.059	0.973	0.039	0.064	0.544	-0.001	0.045	0.979	-0.008	0.044	0.850	0.018	0.023	0.426	1.000	0.818
rs2820292	0.008	0.068	0.906	0.024	0.057	0.671	-0.006	0.051	0.904	-0.002	0.059	0.969	0.011	0.040	0.773	-0.021	0.038	0.586	-3.24E-04	0.020	0.987	1.000	0.988
rs13021737	-0.158	0.119	0.186	-0.042	0.089	0.633	-0.019	0.080	0.809	-0.097	0.098	0.324	0.054	0.063	0.393	0.091	0.064	0.158	0.006	0.032	0.844	1.000	0.336
rs10182181	0.056	0.060	0.352	0.064	0.044	0.146	-0.022	0.044	0.615	-0.071	0.046	0.124	0.004	0.033	0.912	-0.022	0.032	0.479	-0.004	0.016	0.804	1.000	0.300
rs11126666	-0.141	0.060	0.018	-0.029	0.048	0.549	-0.017	0.045	0.713	-0.074	0.049	0.129	0.038	0.035	0.288	0.009	0.035	0.798	-0.017	0.018	0.343	1.000	0.121
rs1016287	0.094	0.065	0.147	-0.032	0.051	0.539	-0.024	0.051	0.634	-0.024	0.051	0.641	0.017	0.036	0.646	-0.002	0.037	0.951	0.001	0.019	0.956	1.000	0.678
rs11688816	0.074	0.062	0.238	-0.116	0.047	0.013	-0.068	0.045	0.131	0.028	0.049	0.570	-0.041	0.036	0.258	-0.030	0.035	0.380	-0.035	0.018	0.047	1.000	0.143
rs1460676	-0.059	0.060	0.325	0.036	0.045	0.419	0.027	0.043	0.533	0.019	0.046	0.679	-0.051	0.034	0.127	0.032	0.033	0.338	0.002	0.017	0.885	1.000	0.368
rs1528435	-0.075	0.062	0.232	-0.089	0.045	0.045	-0.019	0.044	0.655	0.015	0.046	0.744	0.044	0.034	0.196	0.033	0.033	0.314	2.93E-04	0.017	0.986	1.000	0.132
rs17203016	-0.095	0.087	0.276	-0.070	0.064	0.271	0.008	0.059	0.890	0.037	0.069	0.593	-0.057	0.047	0.226	0.019	0.051	0.716	-0.022	0.024	0.354	1.000	0.631
rs7599312	0.098	0.195	0.616	-0.103	0.173	0.551	0.001	0.123	0.991	-0.029	0.160	0.856	-0.029	0.101	0.773	0.027	0.094	0.770	-0.004	0.052	0.932	1.000	0.977
rs492400	0.033	0.073	0.648	-0.013	0.054	0.807	0.007	0.052	0.899	0.062	0.055	0.263	0.009	0.039	0.822	-0.033	0.039	0.403	0.003	0.020	0.861	1.000	0.812
rs2176040	-0.167	0.112	0.138	-0.066	0.088	0.453	0.014	0.079	0.856	-0.185	0.089	0.038	0.014	0.064	0.831	0.042	0.065	0.516	-0.030	0.032	0.346	1.000	0.244
rs6804842	0.002	0.064	0.970	0.043	0.046	0.342	-0.014	0.044	0.751	-0.003	0.048	0.958	-0.013	0.033	0.708	-0.016	0.035	0.652	-0.003	0.017	0.848	1.000	0.933
rs2365389	-0.007	0.081	0.930	-0.057	0.061	0.343	0.060	0.065	0.352	-0.011	0.069	0.878	0.021	0.047	0.659	0.110	0.049	0.025	0.029	0.024	0.226	1.000	0.354
rs3849570	-0.011	0.059	0.852	-0.041	0.044	0.348	0.017	0.041	0.672	-0.089	0.047	0.058	-0.009	0.032	0.785	0.018	0.031	0.558	-0.012	0.016	0.474	1.000	0.465
rs16851483	-0.045	0.065	0.490	0.034	0.052	0.505	0.008	0.046	0.858	-0.102	0.052	0.048	-0.073	0.038	0.054	-0.060	0.038	0.112	-0.044	0.019	0.019	1.000	0.343
rs1516725	-0.117	0.106	0.272	-0.151	0.087	0.082	0.150	0.073	0.039	-0.003	0.090	0.976	0.059	0.057	0.295	0.023	0.055	0.676	0.020	0.029	0.489	1.000	0.098
rs10938397	0.044	0.065	0.497	0.012	0.050	0.814	0.036	0.047	0.452	0.014	0.052	0.788	-0.090	0.036	0.012	0.061	0.035	0.087	0.005	0.018	0.798	1.000	0.066
rs17001654	0.050	0.247	0.840	-0.137	0.225	0.543	0.126	0.132	0.341	0.105	0.195	0.591	0.099	0.119	0.407	0.206	0.108	0.056	0.119	0.060	0.048	1.000	0.841
rs2112347	-0.048	0.056	0.387	0.025	0.045	0.581	0.017	0.042	0.681	0.029	0.047	0.540	-0.046	0.032	0.158	-0.030	0.032	0.348	-0.014	0.016	0.380	1.000	0.592
rs7715256	0.065	0.150	0.667	0.153	0.131	0.240	-0.053	0.109	0.628	-0.049	0.112	0.659	0.036	0.078	0.644	-0.127	0.083	0.125	-0.016	0.042	0.702	1.000	0.491
rs205262	-0.026	0.080	0.749	0.139	0.061	0.023	0.169	0.059	0.004	0.143	0.067	0.033	0.097	0.047	0.038	-0.056	0.046	0.223	0.071	0.023	0.002	1.000	**0.012**
rs2033529	-0.166	0.081	0.042	-0.037	0.059	0.528	-0.080	0.053	0.133	-0.036	0.063	0.562	-0.020	0.043	0.647	0.035	0.044	0.423	-0.031	0.022	0.153	1.000	0.311
rs2207139	-0.028	0.079	0.721	-0.003	0.064	0.960	0.067	0.064	0.298	-0.054	0.068	0.431	-0.022	0.047	0.642	0.032	0.046	0.493	0.002	0.024	0.917	1.000	0.775
rs9400239	0.028	0.063	0.659	0.003	0.045	0.945	0.032	0.046	0.479	-0.095	0.051	0.061	-0.018	0.035	0.619	-0.043	0.034	0.204	-0.020	0.017	0.259	1.000	0.441
rs9374842	-0.097	0.119	0.413	-0.026	0.079	0.743	0.031	0.073	0.673	-0.045	0.075	0.552	0.006	0.058	0.915	-0.017	0.052	0.743	-0.014	0.028	0.621	1.000	0.946
rs13201877	0.333	0.191	0.082	-0.095	0.100	0.341	0.120	0.105	0.254	-0.084	0.110	0.441	0.058	0.081	0.478	0.126	0.084	0.132	0.051	0.041	0.217	1.000	0.214
rs1167827	0.124	0.137	0.367	0.109	0.098	0.269	0.018	0.097	0.850	-0.074	0.092	0.421	0.019	0.066	0.778	0.061	0.062	0.328	0.036	0.034	0.292	1.000	0.753

rs9641123	-0.016	0.061	0.797	0.035	0.046	0.457	-0.001	0.044	0.979	0.044	0.049	0.367	0.055	0.036	0.121	0.034	0.034	0.322	0.031	0.017	0.076	1.000	0.893
rs6465468	0.046	0.271	0.864	-0.008	0.173	0.962	-0.133	0.182	0.466	-0.199	0.284	0.483	0.058	0.136	0.669	0.140	0.144	0.331	0.020	0.072	0.785	1.000	0.834
rs17405819	-0.083	0.061	0.172	-0.040	0.044	0.362	-0.074	0.042	0.080	0.071	0.047	0.132	-0.033	0.032	0.298	0.012	0.031	0.700	-0.019	0.016	0.237	1.000	0.162
rs2033732	0.009	0.057	0.873	-0.079	0.046	0.091	-0.020	0.042	0.645	-0.015	0.047	0.745	-0.008	0.033	0.810	0.023	0.032	0.461	-0.010	0.016	0.557	1.000	0.629
rs4740619	0.076	0.064	0.237	0.057	0.052	0.267	0.010	0.049	0.834	0.022	0.055	0.696	0.028	0.037	0.453	0.046	0.037	0.212	0.038	0.019	0.047	1.000	0.962
rs10968576	-0.050	0.073	0.493	0.004	0.057	0.951	-0.064	0.054	0.236	-0.135	0.062	0.031	-0.011	0.045	0.805	-0.054	0.042	0.203	-0.047	0.021	0.030	1.000	0.615
rs6477694	-0.061	0.059	0.303	-0.026	0.045	0.557	-0.020	0.042	0.639	0.029	0.045	0.520	-0.041	0.032	0.208	-0.034	0.032	0.276	-0.027	0.016	0.102	1.000	0.829
rs1928295	-0.013	0.056	0.812	-0.008	0.044	0.855	0.003	0.042	0.943	-0.035	0.046	0.449	0.020	0.033	0.548	0.004	0.033	0.897	-2.89E-04	0.016	0.986	1.000	0.959
rs10733682	-0.010	0.070	0.883	-0.005	0.055	0.925	-0.023	0.047	0.626	0.025	0.055	0.654	-0.015	0.040	0.715	-0.030	0.039	0.449	-0.013	0.020	0.497	1.000	0.982
rs17094222	0.021	0.059	0.721	-0.016	0.047	0.739	0.034	0.045	0.449	0.013	0.051	0.798	-0.046	0.036	0.209	0.048	0.037	0.192	0.007	0.018	0.697	1.000	0.548
rs11191560	0.030	0.063	0.639	0.054	0.049	0.270	0.015	0.047	0.741	-0.067	0.054	0.219	-0.027	0.036	0.459	-0.059	0.036	0.098	-0.017	0.018	0.342	1.000	0.363
rs7903146	-0.301	0.254	0.236	-0.087	0.131	0.506	0.348	0.173	0.045	-0.186	0.177	0.295	0.168	0.099	0.088	0.069	0.117	0.553	0.058	0.056	0.303	1.000	0.102
rs4256980	0.051	0.063	0.423	0.017	0.045	0.711	0.057	0.043	0.181	-0.023	0.047	0.622	-0.013	0.032	0.689	0.042	0.034	0.217	0.018	0.017	0.275	1.000	0.653
rs11030104	-0.008	0.057	0.896	-0.010	0.042	0.816	0.025	0.040	0.526	-0.084	0.045	0.063	0.061	0.032	0.051	0.023	0.032	0.480	0.013	0.016	0.421	1.000	0.181
rs2176598	-0.082	0.077	0.291	0.080	0.064	0.208	0.048	0.064	0.454	0.037	0.067	0.583	0.041	0.046	0.381	-0.026	0.047	0.576	0.018	0.024	0.435	1.000	0.544
rs3817334	-0.002	0.062	0.974	-0.065	0.050	0.188	-0.051	0.047	0.286	-0.028	0.049	0.565	-0.028	0.036	0.443	0.004	0.035	0.911	-0.025	0.018	0.155	1.000	0.877
rs12286929	0.003	0.065	0.959	-0.006	0.048	0.907	-0.037	0.050	0.459	-0.014	0.050	0.777	-0.009	0.036	0.804	0.032	0.036	0.370	-0.001	0.018	0.941	1.000	0.912
rs7138803	-0.045	0.064	0.478	0.052	0.050	0.295	-0.061	0.045	0.177	-0.002	0.054	0.964	0.032	0.037	0.376	-0.052	0.036	0.151	-0.012	0.018	0.505	1.000	0.323
rs9581854	0.026	0.088	0.767	-0.037	0.066	0.570	-0.108	0.060	0.071	-0.095	0.065	0.142	0.049	0.046	0.288	0.030	0.044	0.495	-0.010	0.023	0.651	1.000	0.204
rs12429545	-0.049	0.067	0.464	0.099	0.048	0.037	-0.008	0.048	0.877	0.114	0.052	0.030	0.087	0.037	0.019	0.047	0.038	0.210	0.058	0.019	0.002	1.000	0.225
rs1441264	0.009	0.059	0.876	-0.032	0.046	0.485	0.084	0.042	0.046	0.007	0.047	0.878	-0.039	0.034	0.257	0.030	0.033	0.362	0.009	0.017	0.577	1.000	0.275
rs10132280	0.147	0.103	0.155	-0.095	0.081	0.241	-0.181	0.082	0.028	-0.087	0.085	0.306	0.027	0.057	0.639	0.005	0.055	0.929	-0.025	0.029	0.388	1.000	0.117
rs12885454	0.108	0.055	0.050	0.063	0.044	0.146	0.026	0.042	0.533	0.039	0.049	0.428	0.064	0.033	0.051	0.011	0.032	0.722	0.046	0.016	0.005	1.000	0.678
rs7141420	-0.011	0.060	0.856	-0.016	0.043	0.706	-0.076	0.041	0.067	-0.067	0.045	0.134	0.029	0.033	0.382	-0.006	0.032	0.855	-0.018	0.016	0.258	1.000	0.379
rs16951275	0.039	0.060	0.514	-0.014	0.043	0.750	-0.005	0.045	0.917	0.008	0.046	0.858	0.056	0.033	0.087	-0.026	0.033	0.431	0.009	0.017	0.578	1.000	0.577
rs7164727	-0.108	0.070	0.122	-0.025	0.049	0.606	0.035	0.050	0.487	0.021	0.052	0.688	0.003	0.037	0.930	0.016	0.035	0.650	0.001	0.018	0.937	1.000	0.621
rs2650492	0.185	0.228	0.418	-0.162	0.099	0.102	0.076	0.104	0.463	0.077	0.117	0.511	0.144	0.141	0.305	0.128	0.105	0.223	0.043	0.048	0.371	1.000	0.301
rs3888190	-0.058	0.109	0.594	0.048	0.084	0.566	0.120	0.072	0.099	-0.007	0.080	0.926	0.136	0.059	0.021	0.020	0.055	0.711	0.058	0.029	0.044	1.000	0.442
rs4787491	-0.121	0.060	0.044	-0.028	0.043	0.514	0.005	0.042	0.904	-0.119	0.046	0.010	-0.062	0.033	0.063	-0.024	0.032	0.449	-0.049	0.016	0.003	1.000	0.270
rs9925964	-0.037	0.090	0.682	-0.040	0.070	0.571	0.056	0.071	0.434	-0.034	0.079	0.666	-0.078	0.051	0.128	-0.016	0.048	0.731	-0.029	0.026	0.256	1.000	0.787
rs2080454	0.011	0.059	0.852	0.008	0.044	0.856	-0.011	0.042	0.786	0.011	0.046	0.805	-0.005	0.032	0.864	0.042	0.031	0.175	0.011	0.016	0.477	1.000	0.905
rs1558902	0.028	0.086	0.742	0.045	0.064	0.479	-3.10E-04	0.066	0.996	-0.039	0.073	0.599	0.068	0.047	0.150	0.022	0.050	0.654	0.029	0.025	0.244	1.000	0.875
rs9914578	0.063	0.075	0.399	0.035	0.053	0.505	0.016	0.052	0.753	-0.089	0.059	0.131	0.008	0.039	0.843	0.006	0.039	0.882	0.005	0.020	0.793	1.000	0.618
rs1000940	-0.106	0.060	0.079	0.011	0.046	0.810	0.030	0.042	0.465	-0.089	0.045	0.050	-0.026	0.033	0.436	-0.011	0.033	0.740	-0.023	0.017	0.169	1.000	0.273
rs12940622	-0.127	0.061	0.038	-0.068	0.049	0.159	-0.019	0.044	0.667	-0.026	0.050	0.602	0.004	0.035	0.902	-0.007	0.035	0.838	-0.026	0.018	0.134	1.000	0.471
rs7239883	0.019	0.061	0.756	-0.032	0.048	0.506	-0.013	0.045	0.777	-0.034	0.051	0.508	-0.016	0.034	0.644	0.048	0.034	0.157	-1.06E-04	0.017	0.995	1.000	0.658
rs7243357	-0.157	0.066	0.018	-0.026	0.054	0.628	0.045	0.058	0.441	-0.028	0.056	0.622	0.063	0.040	0.115	0.020	0.039	0.611	0.005	0.020	0.813	1.000	0.093
rs6567160	0.041	0.076	0.585	0.042	0.060	0.476	-0.085	0.053	0.106	0.039	0.064	0.536	-0.034	0.043	0.429	0.039	0.045	0.396	-0.001	0.022	0.974	1.000	0.398
rs29941	0.137	0.075	0.069	0.081	0.052	0.122	-0.009	0.051	0.856	-0.099	0.055	0.071	-0.023	0.038	0.552	0.021	0.036	0.566	0.006	0.019	0.736	1.000	0.088
rs2075650	0.003	0.103	0.979	-0.023	0.083	0.782	-0.097	0.073	0.185	-0.055	0.091	0.545	-0.163	0.136	0.232	0.151	0.137	0.271	-0.044	0.039	0.264	1.000	0.608
rs2287019	0.135	0.074	0.067	0.081	0.056	0.152	-0.038	0.052	0.468	-0.063	0.062	0.313	0.045	0.090	0.618	-0.015	0.082	0.856	0.017	0.027	0.527	1.000	0.232
rs3810291	0.005	0.066	0.936	0.069	0.048	0.151	-0.031	0.043	0.470	0.025	0.050	0.614	-0.016	0.035	0.646	-0.020	0.036	0.588	-0.001	0.018	0.944	1.000	0.646
rs6091540	-0.027	0.062	0.664	0.043	0.049	0.384	0.014	0.046	0.761	0.105	0.052	0.042	-0.003	0.037	0.928	-0.003	0.035	0.941	0.017	0.018	0.345	1.000	0.507
rs2836754	-0.008	0.061	0.898	0.002	0.045	0.966	-0.090	0.042	0.031	0.051	0.050	0.309	-0.029	0.032	0.371	-0.022	0.033	0.510	-0.022	0.017	0.182	1.000	0.396

SCHS: Singapore Chinese Health Study; SP2: Singapore Prospective Study Program; T2D: Type II diabetes.

Age gender and total calories intake were included in the linear regression model as covariates.

S6 Table: Interaction between SNPs and %SFA on BMI

	SCHS CAD cases N = 594			SCHS CAD controls N = 1070			SP2610 N = 1145			SP21m N = 949			SCHS T2D cases N = 2004			SCHS T2D controls N = 2055			SCHS + SP2610 + SP21m N = 7817				
	Beta	SE	P	Beta	SE	P	Beta	SE	P	Beta	SE	P	Beta	SE	P	Beta	SE	P	Beta	SE	P	P_{adjust}	$Q_{p\text{-}value}$
rs977747	-0.063	0.187	0.734	0.143	0.111	0.197	0.046	0.114	0.686	-0.041	0.104	0.698	-0.059	0.082	0.468	-0.112	0.076	0.141	-0.031	0.041	0.451	1.000	0.518
rs11583200	0.098	0.093	0.295	0.029	0.078	0.711	0.007	0.080	0.931	0.048	0.088	0.587	0.036	0.060	0.549	0.004	0.061	0.944	0.031	0.030	0.302	1.000	0.974
rs3101336	0.019	0.097	0.847	0.015	0.086	0.861	0.028	0.071	0.694	0.151	0.089	0.090	0.048	0.063	0.443	-0.009	0.057	0.880	0.034	0.030	0.256	1.000	0.789
rs12566985	0.001	0.074	0.991	0.086	0.054	0.112	-0.080	0.055	0.143	0.038	0.060	0.535	-0.032	0.042	0.440	0.004	0.042	0.920	-0.001	0.021	0.949	1.000	0.341
rs11165643	0.077	0.078	0.321	0.001	0.057	0.988	0.026	0.054	0.631	0.081	0.057	0.153	0.049	0.042	0.243	-0.033	0.042	0.433	0.025	0.021	0.237	1.000	0.571
rs543874	0.114	0.081	0.158	0.084	0.060	0.167	0.004	0.057	0.947	0.071	0.064	0.267	0.043	0.047	0.357	0.030	0.044	0.496	0.048	0.023	0.033	1.000	0.860
rs2820292	-0.031	0.069	0.653	0.007	0.056	0.895	-0.004	0.052	0.939	0.035	0.057	0.540	0.008	0.039	0.844	0.009	0.039	0.816	0.006	0.020	0.748	1.000	0.988
rs13021737	-0.021	0.108	0.846	0.022	0.082	0.792	-0.031	0.077	0.687	-0.090	0.100	0.367	0.031	0.066	0.637	0.089	0.061	0.147	0.018	0.032	0.571	1.000	0.687
rs10182181	0.040	0.060	0.501	0.065	0.045	0.146	0.003	0.044	0.943	-0.074	0.046	0.106	0.005	0.032	0.878	0.003	0.032	0.930	0.005	0.016	0.778	1.000	0.398
rs11126666	-0.136	0.060	0.023	-0.045	0.049	0.357	-0.097	0.045	0.032	-0.082	0.050	0.103	0.018	0.035	0.612	-2.20E-04	0.036	0.995	-0.039	0.018	0.030	1.000	0.116
rs1016287	0.108	0.065	0.096	-0.014	0.052	0.784	-0.078	0.051	0.130	-0.038	0.052	0.464	0.001	0.037	0.983	0.019	0.037	0.613	-0.003	0.019	0.867	1.000	0.314
rs11688816	0.074	0.060	0.221	-0.027	0.049	0.584	-0.101	0.046	0.027	0.043	0.049	0.380	-0.005	0.036	0.893	-0.036	0.035	0.301	-0.017	0.018	0.328	1.000	0.180
rs1460676	-0.098	0.060	0.102	-0.005	0.046	0.913	0.033	0.044	0.457	0.034	0.044	0.444	-0.067	0.033	0.042	0.038	0.032	0.247	-0.006	0.017	0.721	1.000	0.102
rs1528435	-0.056	0.061	0.358	-0.074	0.045	0.099	-0.004	0.043	0.932	-0.048	0.048	0.315	0.040	0.033	0.229	0.028	0.033	0.399	-0.004	0.017	0.823	1.000	0.244
rs17203016	-0.139	0.089	0.117	-0.077	0.066	0.242	-0.013	0.058	0.825	0.012	0.065	0.857	-0.015	0.046	0.748	0.012	0.050	0.808	-0.022	0.024	0.357	1.000	0.663
rs7599312	0.024	0.220	0.914	-0.361	0.175	0.040	-0.028	0.122	0.816	-0.081	0.182	0.654	0.093	0.100	0.354	-0.037	0.101	0.711	-0.029	0.054	0.592	1.000	0.391
rs492400	0.009	0.071	0.900	-0.031	0.051	0.547	0.011	0.052	0.828	0.003	0.054	0.961	0.034	0.040	0.396	-0.039	0.039	0.320	-0.003	0.020	0.861	1.000	0.834
rs2176040	-0.161	0.109	0.138	0.056	0.085	0.507	0.023	0.078	0.767	-0.062	0.092	0.503	-1.54E-04	0.067	0.998	0.071	0.060	0.240	0.010	0.032	0.749	1.000	0.485
rs6804842	0.008	0.062	0.893	0.051	0.046	0.264	-0.033	0.044	0.464	0.080	0.047	0.091	-0.036	0.033	0.277	-0.030	0.035	0.393	-0.003	0.017	0.851	1.000	0.256
rs2365389	0.060	0.083	0.468	-0.009	0.063	0.890	0.108	0.065	0.098	-0.021	0.067	0.751	0.029	0.046	0.527	0.106	0.047	0.023	0.050	0.024	0.034	1.000	0.486
rs3849570	-0.026	0.058	0.657	-0.030	0.045	0.510	0.013	0.041	0.762	-0.027	0.047	0.569	0.014	0.032	0.657	0.019	0.032	0.544	0.002	0.016	0.924	1.000	0.901
rs16851483	-0.013	0.066	0.839	0.023	0.052	0.655	-0.017	0.047	0.714	-0.050	0.053	0.348	-0.060	0.038	0.117	-0.047	0.038	0.218	-0.034	0.019	0.076	1.000	0.836
rs1516725	-0.180	0.102	0.078	-0.163	0.088	0.064	0.161	0.070	0.021	0.038	0.091	0.671	0.038	0.056	0.501	0.009	0.054	0.873	0.012	0.029	0.677	1.000	**0.030**
rs10938397	0.118	0.065	0.069	0.032	0.050	0.523	-0.032	0.048	0.503	-0.081	0.054	0.135	-0.057	0.036	0.115	0.036	0.036	0.314	-0.006	0.018	0.756	1.000	0.080
rs17001654	-0.077	0.217	0.724	-0.098	0.287	0.733	0.174	0.149	0.244	0.062	0.210	0.766	0.016	0.116	0.889	0.109	0.106	0.307	0.065	0.061	0.289	1.000	0.898
rs2112347	-0.047	0.057	0.404	-0.039	0.046	0.398	0.033	0.041	0.430	0.033	0.047	0.487	-0.004	0.031	0.902	-0.063	0.032	0.048	-0.017	0.016	0.287	1.000	0.376
rs7715256	0.027	0.149	0.856	0.180	0.133	0.178	-0.119	0.129	0.356	0.007	0.120	0.951	0.037	0.083	0.655	-0.058	0.086	0.496	0.004	0.044	0.926	1.000	0.645
rs205262	-0.035	0.081	0.666	0.068	0.060	0.257	0.110	0.063	0.080	0.139	0.067	0.038	0.102	0.046	0.027	-0.045	0.046	0.320	0.053	0.023	0.022	1.000	0.090
rs2033529	-0.190	0.082	0.021	-0.071	0.057	0.215	-0.024	0.053	0.657	-0.097	0.063	0.122	-0.006	0.042	0.887	0.026	0.044	0.560	-0.034	0.022	0.114	1.000	0.197
rs2207139	-0.001	0.076	0.994	0.040	0.065	0.538	0.007	0.063	0.909	-0.096	0.063	0.128	-0.058	0.048	0.220	0.021	0.045	0.645	-0.016	0.023	0.504	1.000	0.552
rs9400239	0.019	0.062	0.753	0.007	0.046	0.878	0.091	0.046	0.049	-0.053	0.052	0.315	-0.035	0.035	0.304	-0.046	0.034	0.170	-0.012	0.017	0.500	1.000	0.187
rs9374842	-0.188	0.126	0.136	-0.040	0.078	0.606	0.050	0.069	0.467	-0.097	0.070	0.169	-0.049	0.057	0.393	0.011	0.055	0.846	-0.030	0.028	0.276	1.000	0.479
rs13201877	0.368	0.178	0.040	-0.008	0.107	0.939	0.149	0.105	0.157	-0.121	0.120	0.314	0.089	0.081	0.274	0.130	0.084	0.123	0.084	0.042	0.046	1.000	0.230
rs1167827	0.007	0.145	0.960	0.117	0.098	0.232	0.111	0.094	0.237	-0.121	0.096	0.206	0.082	0.064	0.202	0.095	0.064	0.133	0.064	0.034	0.059	1.000	0.445
rs9641123	0.017	0.063	0.787	-0.011	0.048	0.814	-0.036	0.043	0.405	0.045	0.050	0.360	0.051	0.036	0.154	0.003	0.035	0.934	0.013	0.018	0.470	1.000	0.672
rs6465468	-0.039	0.263	0.883	-0.011	0.182	0.951	-0.002	0.163	0.991	-0.137	0.337	0.684	0.093	0.141	0.510	0.100	0.128	0.435	0.042	0.070	0.546	1.000	0.972
rs17405819	-0.118	0.060	0.049	-0.039	0.044	0.371	-0.019	0.043	0.654	0.050	0.048	0.291	-0.018	0.031	0.555	0.023	0.031	0.456	-0.010	0.016	0.550	1.000	0.254
rs2033732	0.031	0.058	0.599	-0.098	0.046	0.034	0.002	0.043	0.961	0.032	0.048	0.502	-0.021	0.034	0.545	0.074	0.032	0.023	0.009	0.017	0.607	1.000	0.062
rs4740619	0.097	0.064	0.131	0.087	0.052	0.098	0.028	0.049	0.561	0.034	0.057	0.548	0.003	0.036	0.937	-0.010	0.037	0.796	0.026	0.019	0.169	1.000	0.562
rs10968576	-0.070	0.070	0.320	-0.050	0.058	0.387	0.007	0.055	0.901	-0.085	0.061	0.163	0.002	0.044	0.957	-0.088	0.043	0.041	-0.045	0.022	0.038	1.000	0.611
rs6477694	0.033	0.061	0.594	-0.012	0.045	0.793	-0.020	0.042	0.633	0.035	0.047	0.452	-0.021	0.032	0.509	-0.035	0.031	0.265	-0.013	0.016	0.428	1.000	0.821
rs1928295	0.012	0.059	0.837	-0.027	0.045	0.542	-0.003	0.041	0.943	-0.017	0.046	0.712	0.013	0.033	0.688	0.027	0.033	0.423	0.004	0.016	0.787	1.000	0.936
rs10733682	0.048	0.070	0.490	-0.023	0.055	0.674	0.003	0.048	0.949	-0.040	0.057	0.483	-0.020	0.040	0.609	-0.015	0.039	0.698	-0.012	0.020	0.532	1.000	0.947
rs17094222	0.031	0.059	0.604	-0.008	0.047	0.859	0.022	0.045	0.629	0.004	0.052	0.939	-0.060	0.038	0.113	0.077	0.037	0.036	0.011	0.018	0.552	1.000	0.214
rs11191560	-0.003	0.062	0.963	0.062	0.048	0.194	0.031	0.048	0.522	-0.035	0.055	0.528	-0.071	0.036	0.051	-0.029	0.034	0.395	-0.016	0.018	0.365	1.000	0.286
rs7903146	-0.252	0.268	0.348	-0.207	0.140	0.140	0.267	0.154	0.084	-0.161	0.140	0.252	0.258	0.095	0.007	0.011	0.118	0.922	0.054	0.054	0.317	1.000	**0.017**
rs4256980	0.077	0.063	0.221	-2.63E-04	0.044	0.995	0.055	0.041	0.182	-0.047	0.048	0.320	-0.002	0.031	0.950	0.045	0.033	0.175	0.019	0.016	0.255	1.000	0.438
rs11030104	0.031	0.057	0.583	0.048	0.043	0.265	0.064	0.041	0.117	-0.037	0.044	0.399	0.012	0.032	0.720	0.029	0.032	0.363	0.024	0.016	0.131	1.000	0.639
rs2176598	-0.093	0.079	0.236	0.094	0.063	0.135	0.017	0.064	0.791	0.054	0.070	0.438	0.043	0.046	0.345	-0.013	0.047	0.777	0.021	0.024	0.364	1.000	0.484
rs3817334	0.002	0.061	0.978	-0.029	0.050	0.553	-0.013	0.049	0.784	-0.011	0.050	0.819	0.004	0.036	0.923	0.034	0.035	0.326	0.003	0.018	0.866	1.000	0.921
rs12286929	-0.016	0.066	0.810	-0.075	0.047	0.107	-0.058	0.050	0.243	-0.026	0.051	0.617	-0.013	0.036	0.731	0.024	0.036	0.513	-0.021	0.018	0.251	1.000	0.623
rs7138803	-0.066	0.063	0.294	0.087	0.051	0.085	-0.024	0.046	0.612	0.020	0.053	0.706	0.028	0.037	0.453	-0.031	0.036	0.400	0.004	0.018	0.842	1.000	0.337
rs9581854	-0.020	0.088	0.820	0.012	0.067	0.853	-0.064	0.063	0.307	-0.071	0.067	0.290	0.055	0.048	0.254	0.024	0.045	0.591	0.002	0.024	0.924	1.000	0.574
rs12429545	-0.118	0.067	0.078	0.095	0.047	0.042	-0.012	0.048	0.794	0.141	0.054	0.009	0.068	0.037	0.063	0.051	0.037	0.170	0.051	0.018	0.006	1.000	**0.034**
rs1441264	-0.008	0.060	0.894	-0.024	0.046	0.606	0.107	0.042	0.011	0.045	0.047	0.341	-0.046	0.034	0.177	0.024	0.033	0.455	0.014	0.017	0.399	1.000	0.096
rs10132280	0.213	0.106	0.046	-0.042	0.082	0.606	-0.178	0.084	0.035	-0.075	0.084	0.373	0.049	0.060	0.412	-0.048	0.060	0.421	-0.021	0.030	0.485	1.000	0.065
rs12885454	0.107	0.059	0.071	0.044	0.043	0.308	0.029	0.041	0.479	0.057	0.049	0.248	0.082	0.033	0.012	0.022	0.032	0.501	0.052	0.016	0.002	1.000	0.707
rs7141420	-0.015	0.060	0.799	-0.015	0.044	0.731	-0.084	0.042	0.047	-0.062	0.043	0.149	0.030	0.033	0.371	-0.018	0.033	0.585	-0.022	0.016	0.178	1.000	0.358

rs16951275	0.061	0.059	0.304	-0.014	0.043	0.749	-0.002	0.043	0.957	0.041	0.047	0.376	0.013	0.034	0.697	-0.048	0.033	0.146	-0.001	0.017	0.933	1.000	0.520
rs7164727	-0.191	0.073	0.009	-0.005	0.051	0.923	0.011	0.050	0.825	-0.057	0.051	0.269	-0.009	0.037	0.809	-0.012	0.036	0.743	-0.025	0.019	0.185	1.000	0.251
rs2650492	0.205	0.246	0.405	-0.216	0.110	0.050	0.161	0.111	0.145	0.055	0.112	0.622	0.104	0.135	0.440	0.120	0.129	0.353	0.043	0.052	0.404	1.000	0.176
rs3888190	-0.026	0.112	0.815	0.011	0.086	0.894	0.063	0.071	0.374	0.023	0.083	0.778	0.134	0.058	0.021	0.035	0.056	0.531	0.056	0.029	0.053	1.000	0.712
rs4787491	-0.086	0.060	0.148	-0.028	0.043	0.506	-0.010	0.042	0.810	-0.096	0.046	0.037	-0.056	0.033	0.090	-0.029	0.032	0.374	-0.046	0.016	0.005	1.000	0.712
rs9925964	0.029	0.089	0.744	-0.031	0.069	0.658	0.063	0.074	0.395	0.009	0.076	0.908	-0.100	0.049	0.043	-0.002	0.049	0.963	-0.021	0.026	0.417	1.000	0.483
rs2080454	-0.008	0.058	0.895	0.027	0.044	0.543	-0.043	0.042	0.307	0.038	0.045	0.399	-3.28E-04	0.032	0.992	0.015	0.031	0.638	0.005	0.016	0.736	1.000	0.814
rs1558902	-0.052	0.082	0.523	0.068	0.063	0.284	0.048	0.067	0.472	-0.026	0.076	0.738	0.083	0.046	0.074	0.022	0.050	0.666	0.038	0.025	0.124	1.000	0.670
rs9914578	0.014	0.073	0.847	-0.016	0.054	0.772	-0.009	0.051	0.867	-0.035	0.060	0.564	-0.026	0.040	0.506	-0.034	0.039	0.387	-0.022	0.020	0.274	1.000	0.993
rs1000940	-0.115	0.062	0.062	0.037	0.046	0.416	0.037	0.041	0.368	-0.075	0.046	0.105	-0.044	0.033	0.184	-0.032	0.033	0.322	-0.026	0.017	0.112	1.000	0.166
rs12940622	-0.139	0.064	0.029	-0.091	0.048	0.058	0.001	0.045	0.985	-0.007	0.049	0.888	-0.009	0.035	0.789	0.003	0.035	0.923	-0.025	0.018	0.155	1.000	0.265
rs7239883	0.057	0.063	0.362	-0.029	0.049	0.552	-0.017	0.045	0.713	-0.014	0.051	0.780	-0.024	0.034	0.478	0.046	0.034	0.178	0.002	0.017	0.899	1.000	0.593
rs7243357	-0.136	0.065	0.037	-0.021	0.055	0.706	0.038	0.057	0.504	-0.054	0.057	0.339	0.088	0.041	0.033	0.025	0.039	0.528	0.010	0.020	0.632	1.000	0.059
rs6567160	0.028	0.078	0.717	0.003	0.062	0.962	-0.066	0.055	0.233	0.058	0.062	0.352	0.016	0.042	0.702	0.049	0.046	0.292	0.015	0.022	0.500	1.000	0.666
rs29941	0.107	0.076	0.162	0.089	0.052	0.089	0.023	0.052	0.661	-0.088	0.055	0.113	-0.028	0.038	0.455	0.027	0.036	0.463	0.012	0.019	0.549	1.000	0.140
rs2075650	0.006	0.106	0.955	-0.066	0.081	0.414	-0.105	0.074	0.154	-0.003	0.096	0.975	-0.112	0.135	0.409	0.193	0.134	0.149	-0.038	0.039	0.334	1.000	0.475
rs2287019	0.089	0.073	0.221	0.059	0.056	0.287	-0.015	0.051	0.768	-0.041	0.061	0.501	0.056	0.088	0.523	-0.015	0.080	0.848	0.017	0.026	0.529	1.000	0.665
rs3810291	-0.063	0.064	0.322	0.049	0.046	0.288	-0.051	0.044	0.245	0.038	0.052	0.469	-0.032	0.035	0.363	0.012	0.036	0.744	-0.007	0.018	0.696	1.000	0.444
rs6091540	-0.013	0.061	0.831	0.043	0.048	0.373	-0.016	0.046	0.723	0.036	0.053	0.489	0.005	0.036	0.894	0.014	0.035	0.677	0.012	0.018	0.518	1.000	0.943
rs2836754	-0.014	0.061	0.825	-0.029	0.045	0.527	-0.098	0.042	0.019	0.026	0.050	0.599	-0.026	0.032	0.422	-0.026	0.032	0.426	-0.031	0.017	0.062	1.000	0.547

SCHS: Singapore Chinese Health Study; SP2: Singapore Prospective Study Program; T2D: Type II diabetes.

SFA: Saturated Fatty Acids

Age gender and total calories intake were included in the linear regression model as covariates.

S7 Table: Interaction between SNPs and %MUFA on BMI

	SCHS CAD cases N = 594			SCHS CAD controls N = 1070			SP2610 N = 1145			SP21m N = 949			SCHS T2D cases N = 2004			SCHS T2D controls N = 2055			SCHS + SP2610 + SP21m N = 7817				
	Beta	SE	P	Beta	SE	P	Beta	SE	P	Beta	SE	P	Beta	SE	P	Beta	SE	P	Beta	SE	P	P_{adjust}	$Q_{p\text{-}value}$
rs977747	0.045	0.176	0.798	0.268	0.119	0.025	0.040	0.118	0.735	-0.014	0.095	0.884	-0.083	0.088	0.344	-0.055	0.075	0.466	0.002	0.041	0.959	1.000	0.244
rs11583200	0.081	0.087	0.357	-0.019	0.079	0.809	-0.005	0.080	0.953	0.009	0.094	0.921	0.063	0.062	0.316	0.024	0.057	0.680	0.028	0.030	0.354	1.000	0.941
rs3101336	-0.049	0.101	0.630	-0.185	0.085	0.030	-0.072	0.069	0.300	0.018	0.092	0.841	-0.016	0.062	0.790	-0.054	0.053	0.313	-0.056	0.029	0.054	1.000	0.636
rs12566985	0.007	0.072	0.919	0.112	0.057	0.051	-0.054	0.057	0.339	0.025	0.058	0.669	0.017	0.042	0.681	-0.020	0.043	0.647	0.011	0.021	0.596	1.000	0.414
rs11165643	0.105	0.075	0.161	-0.005	0.058	0.932	0.095	0.051	0.061	-0.012	0.057	0.837	-0.026	0.041	0.526	-0.051	0.041	0.218	0.002	0.021	0.922	1.000	0.190
rs543874	0.068	0.084	0.419	0.012	0.062	0.842	0.053	0.059	0.365	0.139	0.062	0.024	-0.021	0.044	0.638	0.006	0.043	0.891	0.029	0.022	0.198	1.000	0.393
rs2820292	0.009	0.067	0.895	0.012	0.057	0.832	0.040	0.051	0.437	-0.024	0.057	0.678	-0.003	0.039	0.935	-0.041	0.038	0.285	-0.006	0.020	0.745	1.000	0.865
rs13021737	-0.139	0.123	0.257	-0.046	0.087	0.599	0.004	0.074	0.962	-0.037	0.100	0.714	0.034	0.062	0.584	0.085	0.063	0.181	0.012	0.032	0.712	1.000	0.601
rs10182181	0.039	0.060	0.518	0.042	0.043	0.321	-0.019	0.043	0.654	-0.087	0.047	0.062	0.013	0.032	0.680	-0.014	0.031	0.661	-0.005	0.016	0.776	1.000	0.373
rs11126666	-0.119	0.060	0.047	-0.029	0.049	0.554	-0.016	0.046	0.724	-0.085	0.050	0.087	0.046	0.035	0.186	0.038	0.034	0.260	-0.005	0.017	0.777	1.000	0.067
rs1016287	0.091	0.063	0.151	-0.043	0.051	0.409	0.026	0.052	0.624	0.016	0.051	0.749	-0.002	0.036	0.953	0.008	0.038	0.828	0.009	0.019	0.620	1.000	0.715
rs11688816	0.118	0.063	0.061	-0.113	0.046	0.015	-0.059	0.046	0.203	-0.025	0.050	0.621	-0.053	0.036	0.142	-0.016	0.035	0.642	-0.036	0.018	0.043	1.000	0.087
rs1460676	-0.036	0.059	0.541	0.065	0.045	0.147	0.061	0.042	0.142	-0.002	0.046	0.957	-0.070	0.033	0.037	0.025	0.033	0.443	0.005	0.017	0.785	1.000	0.090
rs1528435	-0.098	0.063	0.123	-0.093	0.045	0.038	-0.031	0.045	0.487	0.013	0.046	0.778	0.032	0.034	0.350	0.030	0.034	0.373	-0.008	0.017	0.657	1.000	0.119
rs17203016	-0.062	0.088	0.481	-0.089	0.062	0.150	0.015	0.060	0.808	0.008	0.069	0.911	-0.039	0.047	0.402	-0.026	0.051	0.612	-0.031	0.024	0.197	1.000	0.858
rs7599312	0.026	0.207	0.898	0.024	0.179	0.893	0.108	0.113	0.340	0.030	0.167	0.859	-0.145	0.107	0.176	0.004	0.096	0.968	-0.004	0.052	0.943	1.000	0.730
rs492400	0.049	0.073	0.502	-0.005	0.054	0.925	-0.026	0.050	0.606	0.092	0.055	0.095	0.022	0.040	0.584	-0.030	0.039	0.436	0.008	0.020	0.677	1.000	0.515
rs2176040	-0.152	0.114	0.183	-0.107	0.083	0.198	0.076	0.082	0.358	-0.165	0.090	0.069	0.004	0.063	0.949	0.048	0.066	0.466	-0.025	0.032	0.438	1.000	0.183
rs6804842	-0.024	0.062	0.704	0.048	0.045	0.288	-0.011	0.045	0.803	0.025	0.048	0.603	-0.011	0.034	0.734	-0.012	0.035	0.723	0.001	0.017	0.969	1.000	0.870
rs2365389	-0.035	0.081	0.665	-0.060	0.060	0.315	0.085	0.065	0.190	0.024	0.069	0.728	0.027	0.047	0.570	0.098	0.049	0.046	0.032	0.024	0.181	1.000	0.354
rs3849570	0.017	0.059	0.772	-0.039	0.044	0.376	0.059	0.041	0.149	-0.048	0.047	0.305	-0.016	0.032	0.621	0.004	0.032	0.910	-0.004	0.016	0.822	1.000	0.519
rs16851483	-0.035	0.064	0.589	-0.003	0.052	0.950	0.015	0.046	0.748	-0.039	0.053	0.467	-0.088	0.037	0.019	-0.066	0.038	0.083	-0.044	0.019	0.019	1.000	0.557
rs1516725	-0.048	0.106	0.650	-0.100	0.086	0.246	0.113	0.071	0.111	-0.036	0.089	0.685	0.044	0.056	0.431	0.063	0.054	0.246	0.030	0.029	0.305	1.000	0.395
rs10938397	0.042	0.065	0.514	-0.003	0.051	0.954	0.059	0.048	0.222	0.007	0.051	0.895	-0.079	0.036	0.029	0.081	0.036	0.023	0.014	0.018	0.460	1.000	**0.045**
rs17001654	0.151	0.237	0.523	-0.175	0.221	0.427	0.288	0.143	0.044	-0.049	0.193	0.800	0.019	0.120	0.877	0.210	0.111	0.058	0.114	0.061	0.063	1.000	0.378
rs2112347	-0.029	0.055	0.598	0.044	0.044	0.326	0.030	0.042	0.464	-0.002	0.049	0.964	-0.051	0.032	0.108	-0.033	0.032	0.291	-0.014	0.016	0.378	1.000	0.453
rs7715256	0.007	0.147	0.963	0.050	0.129	0.701	-0.103	0.106	0.334	-0.085	0.117	0.467	0.005	0.078	0.953	-0.155	0.080	0.053	-0.060	0.041	0.145	1.000	0.663
rs205262	-0.028	0.078	0.723	0.160	0.060	0.008	0.125	0.062	0.044	0.121	0.065	0.062	0.078	0.046	0.094	-0.058	0.047	0.222	0.060	0.023	0.010	1.000	**0.030**
rs2033529	-0.147	0.081	0.069	-0.007	0.061	0.912	-0.087	0.053	0.099	-2.07E-05	0.062	1.000	-0.033	0.043	0.437	0.040	0.044	0.357	-0.025	0.022	0.249	1.000	0.289
rs2207139	0.012	0.079	0.883	0.002	0.064	0.974	0.078	0.062	0.214	0.008	0.068	0.908	-0.011	0.047	0.819	0.069	0.046	0.132	0.029	0.024	0.221	1.000	0.790
rs9400239	0.043	0.064	0.501	0.009	0.046	0.843	-0.005	0.046	0.915	-0.089	0.050	0.078	-0.019	0.035	0.596	-0.060	0.034	0.074	-0.028	0.017	0.112	1.000	0.470
rs9374842	-0.111	0.119	0.352	0.045	0.077	0.562	0.026	0.080	0.745	0.028	0.079	0.724	4.05E-04	0.059	0.995	0.020	0.054	0.706	0.013	0.029	0.655	1.000	0.927

rs13201877	0.282	0.196	0.151	-0.095	0.101	0.347	0.096	0.104	0.354	-0.008	0.105	0.940	0.058	0.078	0.462	0.116	0.083	0.161	0.053	0.040	0.191	1.000	0.465
rs1167827	0.166	0.134	0.217	0.072	0.098	0.460	0.050	0.091	0.581	-0.119	0.087	0.173	0.032	0.065	0.626	0.066	0.064	0.302	0.035	0.034	0.303	1.000	0.483
rs9641123	-0.019	0.062	0.764	0.022	0.047	0.630	0.012	0.045	0.789	-0.032	0.050	0.525	0.024	0.036	0.497	0.029	0.034	0.396	0.013	0.017	0.457	1.000	0.921
rs6465468	0.031	0.264	0.908	0.038	0.172	0.823	-0.046	0.174	0.791	0.034	0.244	0.888	0.019	0.137	0.887	0.256	0.155	0.099	0.064	0.072	0.370	1.000	0.836
rs17405819	-0.083	0.061	0.171	-0.036	0.045	0.422	-0.024	0.042	0.577	0.023	0.047	0.625	-0.038	0.032	0.236	-4.40E-04	0.031	0.989	-0.021	0.016	0.192	1.000	0.735
rs2033732	1.48E-04	0.058	0.998	-0.083	0.046	0.070	0.015	0.042	0.713	0.019	0.047	0.690	-0.022	0.033	0.506	0.014	0.031	0.660	-0.008	0.016	0.643	1.000	0.547
rs4740619	0.058	0.063	0.360	0.076	0.052	0.143	0.007	0.049	0.886	0.044	0.055	0.427	0.020	0.036	0.581	0.033	0.038	0.386	0.035	0.019	0.065	1.000	0.937
rs10968576	-0.065	0.074	0.383	0.009	0.057	0.868	-0.088	0.055	0.112	-0.074	0.060	0.214	-0.004	0.044	0.932	-0.044	0.041	0.287	-0.039	0.021	0.067	1.000	0.755
rs6477694	-0.083	0.061	0.178	-0.005	0.045	0.918	-0.056	0.041	0.174	0.002	0.045	0.970	-0.043	0.032	0.180	-0.023	0.031	0.470	-0.032	0.016	0.050	1.000	0.827
rs1928295	-0.037	0.058	0.525	-0.014	0.045	0.761	0.003	0.041	0.940	-0.047	0.047	0.317	0.024	0.033	0.473	0.001	0.033	0.973	-0.004	0.017	0.814	1.000	0.854
rs10733682	-0.006	0.070	0.935	-0.062	0.054	0.249	-0.025	0.047	0.600	0.061	0.054	0.264	-0.006	0.040	0.874	-0.032	0.039	0.412	-0.015	0.020	0.456	1.000	0.699
rs17094222	0.028	0.059	0.639	-0.013	0.048	0.779	0.021	0.046	0.639	0.020	0.051	0.691	-0.054	0.036	0.136	0.014	0.037	0.700	-0.003	0.018	0.846	1.000	0.697
rs11191560	0.052	0.064	0.415	0.061	0.050	0.222	0.015	0.049	0.761	-0.124	0.053	0.020	-0.037	0.036	0.304	-0.050	0.036	0.160	-0.023	0.018	0.212	1.000	0.104
rs7903146	-0.154	0.242	0.525	-0.082	0.126	0.517	0.455	0.198	0.022	0.017	0.166	0.917	0.137	0.096	0.153	0.088	0.116	0.447	0.080	0.055	0.151	1.000	0.247
rs4256980	0.024	0.063	0.705	0.036	0.044	0.411	0.103	0.043	0.018	0.001	0.047	0.984	-0.018	0.032	0.576	0.036	0.033	0.275	0.026	0.017	0.118	1.000	0.358
rs11030104	0.012	0.057	0.830	-0.028	0.042	0.511	0.002	0.040	0.951	-0.042	0.047	0.368	0.030	0.032	0.336	0.041	0.032	0.202	0.010	0.016	0.519	1.000	0.634
rs2176598	-0.118	0.078	0.127	0.133	0.062	0.032	-0.024	0.064	0.710	0.035	0.066	0.591	0.047	0.048	0.326	-0.016	0.046	0.737	0.017	0.024	0.473	1.000	0.161
rs3817334	0.002	0.062	0.979	-0.059	0.050	0.233	0.012	0.048	0.801	-0.032	0.051	0.528	-0.024	0.036	0.492	-0.001	0.035	0.971	-0.016	0.018	0.360	1.000	0.911
rs12286929	0.034	0.067	0.612	0.014	0.048	0.769	-0.038	0.050	0.445	-0.117	0.050	0.020	0.012	0.036	0.737	0.016	0.036	0.656	-0.009	0.018	0.634	1.000	0.265
rs7138803	-0.065	0.065	0.321	0.043	0.051	0.400	-0.097	0.046	0.036	0.006	0.054	0.918	-0.001	0.037	0.975	-0.024	0.035	0.498	-0.021	0.018	0.251	1.000	0.384
rs9581854	0.054	0.088	0.540	-0.031	0.067	0.646	-0.078	0.058	0.180	-0.102	0.067	0.127	0.063	0.047	0.178	0.017	0.043	0.694	-0.004	0.023	0.848	1.000	0.248
rs12429545	-0.018	0.068	0.793	0.100	0.049	0.042	-0.056	0.048	0.250	0.089	0.053	0.093	0.090	0.037	0.014	0.026	0.038	0.493	0.046	0.019	0.014	1.000	0.113
rs1441264	0.016	0.062	0.797	-0.018	0.046	0.701	0.053	0.042	0.203	0.014	0.046	0.768	-0.063	0.035	0.066	0.053	0.033	0.106	0.008	0.017	0.631	1.000	0.174
rs10132280	0.089	0.102	0.385	-0.125	0.079	0.114	-0.125	0.082	0.129	-0.046	0.092	0.619	0.044	0.056	0.432	0.015	0.056	0.782	-0.014	0.029	0.633	1.000	0.281
rs12885454	0.100	0.053	0.061	0.069	0.044	0.119	0.031	0.042	0.458	-0.015	0.051	0.773	0.051	0.033	0.120	-0.004	0.033	0.900	0.034	0.016	0.037	1.000	0.452
rs7141420	-0.033	0.060	0.581	-0.004	0.044	0.919	-0.016	0.042	0.704	-0.044	0.045	0.336	0.042	0.033	0.206	-0.010	0.032	0.762	-0.004	0.016	0.830	1.000	0.695
rs16951275	0.067	0.059	0.262	0.002	0.043	0.959	0.032	0.043	0.460	0.038	0.047	0.419	0.032	0.033	0.324	-0.005	0.033	0.871	0.022	0.016	0.188	1.000	0.884
rs7164727	-0.138	0.071	0.052	0.001	0.049	0.990	0.051	0.048	0.289	-0.081	0.052	0.121	0.006	0.037	0.879	0.018	0.035	0.617	-0.006	0.018	0.759	1.000	0.186
rs2650492	0.031	0.226	0.892	-0.219	0.102	0.033	0.076	0.099	0.443	0.028	0.111	0.797	0.086	0.127	0.502	0.123	0.105	0.242	0.014	0.047	0.768	1.000	0.223
rs3888190	-0.090	0.109	0.412	0.048	0.084	0.571	0.115	0.073	0.117	0.010	0.082	0.905	0.132	0.059	0.025	0.029	0.055	0.603	0.059	0.029	0.042	1.000	0.460
rs4787491	-0.066	0.059	0.266	-3.62E-04	0.043	0.993	-0.015	0.041	0.724	-0.071	0.046	0.124	-0.037	0.033	0.270	-0.017	0.032	0.593	-0.029	0.016	0.071	1.000	0.852
rs9925964	0.027	0.094	0.776	-0.038	0.069	0.583	0.057	0.072	0.428	0.018	0.082	0.829	-0.101	0.053	0.057	-0.019	0.048	0.690	-0.024	0.026	0.354	1.000	0.552
rs2080454	0.015	0.059	0.793	0.010	0.044	0.818	-0.018	0.042	0.676	-0.003	0.045	0.946	0.009	0.032	0.778	0.039	0.032	0.222	0.012	0.016	0.459	1.000	0.932
rs1558902	0.031	0.088	0.720	0.055	0.064	0.385	-0.043	0.066	0.513	-0.072	0.069	0.295	0.028	0.046	0.535	0.020	0.049	0.689	0.008	0.024	0.727	1.000	0.726
rs9914578	0.006	0.074	0.935	0.055	0.054	0.307	0.009	0.052	0.861	-0.054	0.061	0.370	-0.007	0.039	0.855	0.007	0.039	0.860	0.003	0.020	0.863	1.000	0.860
rs1000940	-0.093	0.060	0.121	0.011	0.046	0.807	-0.024	0.041	0.553	-0.028	0.046	0.537	-0.014	0.033	0.669	0.004	0.032	0.893	-0.015	0.016	0.346	1.000	0.773
rs12940622	-0.107	0.060	0.077	-0.053	0.049	0.279	0.003	0.045	0.954	-0.052	0.050	0.297	0.032	0.034	0.352	0.004	0.035	0.905	-0.013	0.018	0.475	1.000	0.329
rs7239883	0.021	0.063	0.740	-0.018	0.048	0.703	0.006	0.045	0.897	-0.066	0.050	0.186	-0.013	0.034	0.709	0.044	0.034	0.191	4.19E-04	0.017	0.981	1.000	0.568
rs7243357	-0.143	0.068	0.037	-0.019	0.053	0.725	0.078	0.058	0.177	-0.085	0.057	0.134	0.062	0.040	0.121	0.028	0.039	0.472	0.007	0.020	0.735	1.000	**0.044**
rs6567160	0.071	0.078	0.367	0.023	0.060	0.700	-0.074	0.055	0.182	0.023	0.062	0.709	-0.042	0.042	0.318	0.019	0.046	0.675	-0.007	0.022	0.744	1.000	0.556
rs29941	0.142	0.075	0.059	0.082	0.053	0.122	-0.028	0.051	0.578	-0.073	0.056	0.191	-0.012	0.038	0.755	-0.003	0.037	0.943	0.004	0.019	0.845	1.000	0.153
rs2075650	0.064	0.101	0.526	-0.005	0.083	0.956	-0.040	0.075	0.596	-0.042	0.087	0.631	-0.194	0.149	0.195	0.073	0.139	0.599	-0.018	0.039	0.640	1.000	0.752
rs2287019	0.169	0.074	0.022	0.070	0.055	0.200	-0.051	0.051	0.316	-0.047	0.063	0.452	0.063	0.091	0.488	0.022	0.087	0.799	0.024	0.027	0.372	1.000	0.145
rs3810291	-0.020	0.065	0.756	0.066	0.048	0.171	-0.038	0.043	0.377	0.023	0.051	0.650	-0.006	0.036	0.868	-0.012	0.036	0.744	-0.001	0.018	0.972	1.000	0.686
rs6091540	-0.035	0.061	0.562	0.041	0.050	0.407	0.024	0.045	0.593	0.100	0.053	0.057	-0.018	0.036	0.616	-0.007	0.035	0.834	0.011	0.018	0.526	1.000	0.438
rs2836754	0.035	0.060	0.562	-0.026	0.045	0.569	-0.120	0.042	0.004	0.019	0.049	0.700	-0.024	0.032	0.463	-0.031	0.033	0.339	-0.031	0.017	0.058	1.000	0.239

SCHS: Singapore Chinese Health Study; SP2: Singapore Prospective Study Program; T2D: Type II diabetes.
MUFA: Monounsaturated Fatty Acids
Age gender and total calories intake were included in the linear regression model as covariates.

S8 Table: Interaction between SNPs and %PUFA on BMI

	SCHS CAD cases N = 594			SCHS CAD controls N = 1070			SP2610 N = 1145			SP21m N = 949			SCHS T2D cases N = 2004			SCHS T2D controls N = 2055			SCHS + SP2610 + SP21m N = 7817				
	Beta	SE	P	Beta	SE	P	Beta	SE	P	Beta	SE	P	Beta	SE	P	Beta	SE	P	Beta	SE	P	P_{adjust}	$Q_{p\text{-value}}$
rs977747	-0.086	0.160	0.591	0.081	0.126	0.522	0.060	0.116	0.604	-0.152	0.106	0.153	-0.042	0.092	0.647	0.065	0.089	0.464	-0.008	0.044	0.860	1.000	0.590
rs11583200	0.120	0.106	0.260	0.010	0.076	0.894	-0.037	0.078	0.637	0.044	0.091	0.630	0.013	0.059	0.831	0.042	0.056	0.456	0.025	0.030	0.401	1.000	0.896
rs3101336	-0.143	0.114	0.209	-0.245	0.082	0.003	-0.044	0.077	0.567	-0.108	0.089	0.228	-0.042	0.063	0.507	-0.062	0.054	0.252	-0.090	0.030	0.003	1.000	0.418
rs12566985	-0.022	0.076	0.776	0.085	0.057	0.133	0.038	0.056	0.500	-0.036	0.064	0.574	0.017	0.043	0.700	-2.66E-04	0.041	0.995	0.016	0.021	0.455	1.000	0.746
rs11165643	0.047	0.081	0.559	0.001	0.056	0.991	0.015	0.053	0.776	-0.028	0.057	0.620	-0.099	0.041	0.015	0.006	0.039	0.880	-0.022	0.020	0.292	1.000	0.360
rs543874	-0.026	0.083	0.757	0.080	0.063	0.209	-0.006	0.059	0.922	-0.034	0.066	0.606	-0.020	0.043	0.646	-0.051	0.042	0.229	-0.016	0.022	0.469	1.000	0.687
rs2820292	0.043	0.066	0.513	0.062	0.059	0.293	-0.044	0.052	0.391	-0.028	0.058	0.620	0.045	0.041	0.279	-0.021	0.038	0.577	0.006	0.020	0.771	1.000	0.562
rs13021737	-0.233	0.119	0.051	-0.067	0.088	0.444	-0.002	0.080	0.979	-0.051	0.104	0.622	0.044	0.062	0.479	0.016	0.062	0.797	-0.015	0.032	0.635	1.000	0.415
rs10182181	0.030	0.060	0.620	0.029	0.044	0.507	-0.051	0.044	0.248	0.026	0.045	0.557	0.002	0.033	0.961	-0.024	0.031	0.438	-0.003	0.016	0.833	1.000	0.711
rs11126666	-0.063	0.061	0.301	-0.014	0.046	0.767	0.069	0.047	0.144	-0.051	0.050	0.301	0.045	0.034	0.184	-0.001	0.035	0.981	0.008	0.017	0.658	1.000	0.312
rs1016287	-0.001	0.064	0.989	-0.033	0.047	0.485	0.043	0.048	0.378	-0.023	0.052	0.658	0.024	0.037	0.518	-0.033	0.038	0.391	-0.003	0.019	0.853	1.000	0.764
rs11688816	0.010	0.066	0.877	-0.117	0.049	0.018	-0.010	0.045	0.817	0.001	0.048	0.978	-0.040	0.037	0.280	-0.004	0.035	0.908	-0.026	0.018	0.138	1.000	0.450
rs1460676	0.026	0.059	0.659	0.052	0.047	0.263	-0.014	0.041	0.729	0.021	0.047	0.659	0.003	0.034	0.927	0.006	0.033	0.853	0.011	0.017	0.495	1.000	0.931
rs1528435	-0.028	0.066	0.667	-0.059	0.045	0.191	-0.017	0.044	0.708	0.082	0.049	0.095	0.035	0.035	0.309	0.020	0.033	0.535	0.011	0.017	0.513	1.000	0.324
rs17203016	-0.012	0.084	0.888	0.003	0.062	0.967	-0.021	0.062	0.734	0.052	0.065	0.424	-0.068	0.045	0.129	0.031	0.051	0.544	-0.009	0.024	0.699	1.000	0.653
rs7599312	0.155	0.196	0.429	0.267	0.181	0.140	-0.124	0.140	0.377	0.100	0.144	0.487	-0.074	0.104	0.477	0.035	0.088	0.694	0.022	0.052	0.668	1.000	0.473
rs492400	0.047	0.079	0.547	0.004	0.054	0.936	0.013	0.051	0.800	0.010	0.057	0.864	-0.021	0.038	0.589	0.011	0.039	0.771	0.004	0.020	0.836	1.000	0.977
rs2176040	-0.017	0.113	0.880	-0.196	0.091	0.032	0.004	0.083	0.963	-0.165	0.083	0.046	0.019	0.064	0.763	-0.007	0.062	0.912	-0.046	0.032	0.149	1.000	0.248
rs6804842	0.043	0.061	0.480	0.024	0.045	0.592	0.012	0.044	0.794	-0.080	0.048	0.092	3.08E-04	0.034	0.993	-0.001	0.033	0.987	-0.002	0.017	0.915	1.000	0.596
rs2365389	-0.108	0.080	0.178	-0.048	0.060	0.429	-0.006	0.065	0.932	0.031	0.070	0.659	-0.028	0.047	0.557	0.043	0.049	0.375	-0.011	0.024	0.642	1.000	0.614
rs3849570	-0.005	0.061	0.936	-0.053	0.043	0.219	0.014	0.041	0.730	-0.102	0.050	0.042	-0.018	0.032	0.577	0.021	0.031	0.505	-0.015	0.016	0.345	1.000	0.340
rs16851483	-0.019	0.064	0.772	0.024	0.050	0.624	0.051	0.047	0.269	-0.067	0.051	0.188	-0.032	0.038	0.406	-0.071	0.037	0.055	-0.024	0.019	0.192	1.000	0.310
rs1516725	-0.020	0.119	0.864	-0.070	0.088	0.424	-0.014	0.074	0.848	-0.098	0.090	0.278	0.063	0.061	0.296	-0.036	0.055	0.510	-0.018	0.030	0.551	1.000	0.691
rs10938397	-0.055	0.068	0.422	0.006	0.048	0.893	0.042	0.047	0.377	0.118	0.052	0.023	-0.050	0.036	0.172	0.037	0.036	0.294	0.015	0.018	0.409	1.000	0.113
rs17001654	0.089	0.301	0.767	-0.119	0.181	0.510	-0.139	0.140	0.321	-0.052	0.198	0.793	0.171	0.122	0.163	0.231	0.106	0.029	0.076	0.060	0.205	1.000	0.244
rs2112347	-0.076	0.058	0.186	0.073	0.045	0.100	-0.026	0.042	0.541	-0.033	0.046	0.474	-0.046	0.033	0.169	0.025	0.033	0.441	-0.009	0.016	0.575	1.000	0.184
rs7715256	0.114	0.178	0.519	0.058	0.114	0.613	0.011	0.113	0.920	-0.146	0.135	0.278	0.088	0.084	0.299	-0.127	0.085	0.135	-0.008	0.044	0.847	1.000	0.399
rs205262	-0.004	0.080	0.958	0.152	0.061	0.013	0.084	0.063	0.183	0.019	0.066	0.777	0.061	0.048	0.198	-0.018	0.046	0.695	0.046	0.023	0.048	1.000	0.305
rs2033529	-0.073	0.085	0.395	0.032	0.061	0.602	-0.071	0.052	0.173	0.052	0.059	0.382	-0.024	0.044	0.588	0.019	0.044	0.667	-0.007	0.022	0.740	1.000	0.552
rs2207139	-0.075	0.075	0.317	-0.026	0.061	0.675	0.087	0.063	0.164	0.085	0.072	0.242	0.045	0.046	0.321	-0.032	0.047	0.502	0.014	0.023	0.548	1.000	0.351
rs9400239	0.008	0.064	0.895	-0.021	0.046	0.647	-0.013	0.045	0.772	-0.037	0.050	0.461	0.033	0.035	0.349	-0.003	0.034	0.923	-0.002	0.017	0.930	1.000	0.884
rs9374842	0.029	0.113	0.798	0.004	0.074	0.961	-0.045	0.077	0.560	0.058	0.088	0.512	0.083	0.058	0.156	-0.061	0.054	0.262	0.005	0.029	0.850	1.000	0.539
rs13201877	0.095	0.198	0.632	-0.114	0.109	0.297	-0.078	0.107	0.466	-0.108	0.110	0.327	0.054	0.089	0.545	0.046	0.090	0.609	-0.020	0.043	0.652	1.000	0.664
rs1167827	0.134	0.123	0.275	0.035	0.100	0.727	-0.212	0.093	0.022	0.054	0.091	0.553	-0.043	0.064	0.501	-0.036	0.062	0.565	-0.028	0.033	0.396	1.000	0.222
rs9641123	-0.058	0.061	0.346	0.106	0.046	0.021	0.019	0.045	0.674	-0.010	0.048	0.843	0.027	0.035	0.435	0.075	0.034	0.026	0.038	0.017	0.027	1.000	0.211
rs6465468	0.154	0.263	0.559	-0.003	0.176	0.986	-0.154	0.185	0.405	-0.283	0.251	0.259	-0.036	0.135	0.788	0.039	0.144	0.788	-0.036	0.072	0.615	1.000	0.821
rs17405819	0.005	0.059	0.933	0.008	0.044	0.862	-0.058	0.042	0.165	-0.009	0.046	0.839	-0.001	0.032	0.972	-0.009	0.031	0.763	-0.011	0.016	0.490	1.000	0.898
rs2033732	-0.031	0.056	0.583	0.019	0.045	0.679	-0.025	0.042	0.545	-0.068	0.045	0.134	-0.001	0.033	0.974	-0.039	0.031	0.210	-0.023	0.016	0.149	1.000	0.765
rs4740619	0.051	0.066	0.437	0.011	0.050	0.829	-0.007	0.048	0.884	0.014	0.056	0.801	0.027	0.038	0.486	0.112	0.037	0.002	0.042	0.019	0.025	1.000	0.356
rs10968576	0.043	0.079	0.590	0.049	0.058	0.402	-0.087	0.054	0.105	-0.035	0.060	0.558	-0.017	0.044	0.702	0.016	0.043	0.718	-0.009	0.022	0.683	1.000	0.539
rs6477694	-0.088	0.057	0.119	-0.046	0.045	0.305	0.012	0.043	0.787	0.016	0.046	0.720	-0.043	0.033	0.187	-0.038	0.032	0.240	-0.030	0.016	0.063	1.000	0.644
rs1928295	0.001	0.056	0.989	0.059	0.045	0.189	0.045	0.041	0.276	2.25E-04	0.046	0.996	0.020	0.033	0.546	-0.019	0.033	0.567	0.015	0.016	0.352	1.000	0.739
rs10733682	-0.094	0.065	0.146	0.031	0.051	0.547	-0.049	0.051	0.337	0.084	0.056	0.135	0.028	0.040	0.488	-0.005	0.039	0.907	0.004	0.020	0.821	1.000	0.297
rs17094222	-0.016	0.064	0.798	-0.022	0.046	0.628	0.031	0.047	0.518	0.016	0.050	0.749	-0.015	0.037	0.679	-0.020	0.037	0.595	-0.007	0.018	0.717	1.000	0.950
rs11191560	-0.008	0.070	0.910	0.031	0.048	0.518	0.010	0.047	0.822	-0.065	0.053	0.221	0.024	0.036	0.495	-0.078	0.036	0.030	-0.016	0.018	0.387	1.000	0.268
rs7903146	-0.188	0.204	0.359	0.041	0.150	0.787	0.013	0.147	0.927	-0.082	0.183	0.656	-0.052	0.105	0.619	0.065	0.107	0.545	-0.011	0.056	0.843	1.000	0.889
rs4256980	0.030	0.063	0.632	0.014	0.044	0.744	-0.006	0.043	0.895	0.021	0.045	0.642	-0.015	0.031	0.625	-0.013	0.034	0.702	-0.001	0.016	0.940	1.000	0.966
rs11030104	-0.080	0.057	0.163	-0.075	0.042	0.074	-0.058	0.041	0.161	-0.069	0.045	0.124	0.110	0.031	0.000	0.007	0.032	0.839	-0.004	0.016	0.795	1.000	**0.001**
rs2176598	-0.040	0.083	0.630	-0.042	0.059	0.474	0.046	0.058	0.433	-0.022	0.066	0.738	-0.015	0.048	0.763	-0.002	0.045	0.957	-0.009	0.023	0.693	1.000	0.923
rs3817334	-0.012	0.062	0.850	-0.059	0.047	0.212	-0.043	0.048	0.373	-0.052	0.049	0.289	-0.028	0.035	0.427	-0.036	0.034	0.288	-0.038	0.018	0.030	1.000	0.990
rs12286929	0.012	0.066	0.850	0.043	0.051	0.394	0.005	0.048	0.915	0.058	0.050	0.244	-0.036	0.036	0.309	0.037	0.035	0.289	0.015	0.018	0.396	1.000	0.613
rs7138803	-0.038	0.065	0.565	-0.040	0.050	0.419	-0.064	0.047	0.174	-0.026	0.053	0.621	0.053	0.036	0.138	-0.071	0.036	0.052	-0.025	0.018	0.166	1.000	0.209
rs9581854	0.106	0.096	0.272	-0.045	0.067	0.500	-0.068	0.055	0.215	0.003	0.066	0.966	0.016	0.047	0.734	0.011	0.043	0.804	-0.004	0.023	0.861	1.000	0.646
rs12429545	0.021	0.067	0.753	0.041	0.050	0.413	0.014	0.047	0.765	0.026	0.055	0.630	0.047	0.038	0.207	0.039	0.037	0.292	0.035	0.019	0.065	1.000	0.995
rs1441264	-0.009	0.058	0.881	-0.017	0.045	0.702	0.010	0.042	0.813	0.008	0.045	0.853	-0.012	0.033	0.709	0.010	0.033	0.774	-0.001	0.017	0.947	1.000	0.993
rs10132280	0.105	0.106	0.320	-0.023	0.081	0.778	-0.025	0.080	0.749	-0.146	0.086	0.089	0.010	0.056	0.863	0.031	0.052	0.558	-0.003	0.029	0.916	1.000	0.488
rs12885454	0.061	0.056	0.276	0.028	0.044	0.528	0.015	0.041	0.708	2.05E-04	0.048	0.997	0.027	0.033	0.419	-0.010	0.032	0.748	0.015	0.016	0.348	1.000	0.901
rs7141420	0.001	0.059	0.989	-0.015	0.044	0.731	-0.046	0.042	0.269	-0.051	0.048	0.284	-0.034	0.033	0.305	0.015	0.031	0.638	-0.019	0.016	0.239	1.000	0.794

rs16951275	-4.74E-04	0.064	0.994	0.012	0.043	0.780	-0.006	0.044	0.887	-0.059	0.047	0.208	0.081	0.032	0.012	0.025	0.033	0.443	0.021	0.017	0.206	1.000	0.226
rs7164727	0.079	0.065	0.225	-0.026	0.048	0.588	0.053	0.050	0.293	0.098	0.052	0.057	0.001	0.036	0.983	0.040	0.034	0.238	0.033	0.018	0.069	1.000	0.467
rs2650492	-0.065	0.212	0.758	-0.009	0.092	0.925	0.004	0.099	0.967	0.017	0.117	0.886	0.147	0.138	0.284	0.199	0.113	0.077	0.051	0.047	0.283	1.000	0.660
rs3888190	-0.062	0.104	0.554	0.063	0.080	0.433	0.070	0.075	0.353	0.077	0.075	0.305	-1.73E-04	0.059	0.998	0.014	0.056	0.802	0.029	0.029	0.319	1.000	0.855
rs4787491	-0.142	0.059	0.017	-0.001	0.043	0.982	0.038	0.044	0.380	-0.066	0.046	0.150	-0.038	0.033	0.246	-0.007	0.033	0.829	-0.025	0.016	0.121	1.000	0.183
rs9925964	-0.056	0.089	0.532	-0.011	0.068	0.872	-0.023	0.065	0.720	-0.054	0.076	0.482	0.032	0.054	0.553	-0.028	0.049	0.566	-0.016	0.026	0.522	1.000	0.934
rs2080454	0.048	0.059	0.417	-0.016	0.045	0.730	0.092	0.041	0.025	-0.025	0.046	0.590	-0.013	0.031	0.673	0.044	0.032	0.178	0.020	0.016	0.213	1.000	0.255
rs1558902	0.171	0.083	0.039	0.037	0.065	0.576	-0.049	0.066	0.452	-0.009	0.077	0.903	0.018	0.050	0.725	0.032	0.051	0.533	0.025	0.025	0.316	1.000	0.458
rs9914578	0.124	0.073	0.090	0.049	0.053	0.355	0.025	0.053	0.637	-0.079	0.058	0.173	0.038	0.039	0.330	0.063	0.040	0.111	0.037	0.020	0.067	1.000	0.311
rs1000940	-0.033	0.059	0.583	-0.022	0.044	0.622	0.043	0.042	0.311	-0.058	0.046	0.212	-0.006	0.032	0.860	0.031	0.034	0.358	-3.62E-04	0.016	0.982	1.000	0.549
rs12940622	-0.098	0.061	0.111	0.010	0.047	0.831	-0.034	0.045	0.453	0.007	0.048	0.882	0.025	0.035	0.467	-0.020	0.035	0.568	-0.009	0.018	0.592	1.000	0.587
rs7239883	-0.003	0.059	0.965	0.022	0.047	0.640	0.015	0.043	0.736	-0.054	0.049	0.277	0.029	0.033	0.390	0.037	0.034	0.270	0.015	0.017	0.364	1.000	0.753
rs7243357	-0.107	0.068	0.115	-0.030	0.055	0.584	-0.047	0.056	0.403	-0.030	0.055	0.583	0.038	0.041	0.352	-0.007	0.039	0.859	-0.016	0.020	0.421	1.000	0.543
rs6567160	0.043	0.077	0.577	0.050	0.055	0.366	-0.036	0.057	0.529	0.047	0.063	0.450	-0.063	0.042	0.132	-0.018	0.044	0.679	-0.010	0.022	0.656	1.000	0.501
rs29941	0.070	0.071	0.325	0.042	0.053	0.426	-0.045	0.056	0.421	-4.35E-04	0.057	0.994	-0.006	0.038	0.881	-0.013	0.037	0.728	4.15E-04	0.020	0.983	1.000	0.792
rs2075650	-0.006	0.100	0.949	0.015	0.092	0.868	-0.019	0.079	0.813	0.002	0.081	0.984	-0.203	0.158	0.199	0.115	0.141	0.414	-0.006	0.040	0.875	1.000	0.795
rs2287019	0.067	0.073	0.359	0.068	0.057	0.231	-0.036	0.051	0.484	-0.091	0.063	0.147	-0.026	0.089	0.774	-0.017	0.080	0.835	-0.006	0.027	0.814	1.000	0.422
rs3810291	0.141	0.066	0.032	0.050	0.048	0.301	-0.010	0.044	0.821	0.032	0.050	0.529	0.025	0.036	0.481	-0.041	0.037	0.266	0.016	0.018	0.361	1.000	0.218
rs6091540	-0.060	0.067	0.373	0.021	0.048	0.671	0.030	0.044	0.494	0.100	0.049	0.042	0.022	0.036	0.542	-0.044	0.036	0.223	0.011	0.018	0.521	1.000	0.216
rs2836754	-0.023	0.058	0.692	0.021	0.044	0.626	-0.011	0.042	0.791	0.017	0.048	0.725	-0.021	0.032	0.517	0.009	0.033	0.795	-0.002	0.016	0.910	1.000	0.957

SCHS: Singapore Chinese Health Study; SP2: Singapore Prospective Study Program; T2D: Type II diabetes.

PUFA: Polyunsaturated Fatty Acids

Age gender and total calories intake were included in the linear regression model as covariates,

S9 Table: Interaction between SNPs and %carbohydrate on BMI

	SCHS CAD cases N = 594			SCHS CAD controls N = 1070			SP2610 N = 1145			SP21m N = 949			SCHS T2D cases N = 2004			SCHS T2D controls N = 2055			SCHS + SP2610 + SP21m N = 7817				
	Beta	SE	P	Beta	SE	P	Beta	SE	P	Beta	SE	P	Beta	SE	P	Beta	SE	P	Beta	SE	P	P_{adjust}	$Q_{p\text{-}value}$
rs977747	0.099	0.185	0.592	-0.259	0.116	0.025	-0.029	0.123	0.810	0.064	0.104	0.539	0.070	0.091	0.444	0.107	0.077	0.167	0.025	0.043	0.558	1.000	0.163
rs11583200	-0.105	0.088	0.233	0.022	0.080	0.780	-0.025	0.078	0.751	-0.056	0.087	0.521	-0.073	0.061	0.232	-0.065	0.059	0.270	-0.053	0.030	0.077	1.000	0.910
rs3101336	0.100	0.108	0.355	0.133	0.085	0.115	0.046	0.070	0.507	-0.047	0.092	0.610	0.040	0.060	0.511	0.058	0.055	0.291	0.053	0.029	0.071	1.000	0.801
rs12566985	0.003	0.075	0.963	-0.114	0.056	0.042	0.054	0.055	0.331	-0.002	0.060	0.979	-0.028	0.041	0.488	0.006	0.044	0.897	-0.015	0.021	0.487	1.000	0.401
rs11165643	-0.136	0.077	0.076	0.031	0.057	0.584	-0.077	0.052	0.143	-0.037	0.056	0.505	-0.010	0.043	0.808	0.073	0.041	0.075	-0.007	0.021	0.745	1.000	0.102
rs543874	-0.056	0.089	0.526	-0.093	0.061	0.127	0.013	0.059	0.831	-0.051	0.064	0.426	-0.026	0.046	0.568	-0.018	0.041	0.669	-0.032	0.022	0.151	1.000	0.867
rs2820292	-0.031	0.066	0.637	0.006	0.056	0.908	0.016	0.052	0.764	0.011	0.061	0.852	-0.005	0.040	0.899	0.057	0.039	0.145	0.015	0.020	0.447	1.000	0.859
rs13021737	0.129	0.126	0.305	0.095	0.087	0.280	0.006	0.079	0.936	0.105	0.097	0.281	-0.029	0.061	0.629	-0.085	0.064	0.182	0.004	0.032	0.906	1.000	0.369
rs10182181	-0.046	0.059	0.439	-0.092	0.044	0.036	0.063	0.044	0.151	0.057	0.046	0.221	0.009	0.033	0.786	0.022	0.032	0.487	0.008	0.016	0.639	1.000	0.113
rs11126666	0.118	0.060	0.049	0.003	0.047	0.956	-0.003	0.046	0.954	0.088	0.048	0.068	-0.034	0.035	0.324	-0.016	0.035	0.645	0.009	0.017	0.606	1.000	0.147
rs1016287	-0.090	0.065	0.171	0.027	0.051	0.593	0.058	0.052	0.260	0.014	0.051	0.779	-0.014	0.036	0.707	0.012	0.038	0.753	0.005	0.019	0.782	1.000	0.596
rs11688816	-0.043	0.062	0.485	0.057	0.046	0.212	0.034	0.045	0.455	-0.011	0.049	0.829	0.028	0.036	0.442	0.046	0.035	0.187	0.027	0.018	0.124	1.000	0.758
rs1460676	0.092	0.062	0.138	0.024	0.045	0.598	-0.051	0.042	0.223	-0.012	0.046	0.787	0.033	0.033	0.323	-0.033	0.033	0.307	-1.21E-04	0.017	0.994	1.000	0.300
rs1528435	0.026	0.063	0.679	0.102	0.046	0.028	0.006	0.044	0.894	0.010	0.047	0.828	-0.025	0.034	0.457	-0.007	0.033	0.833	0.010	0.017	0.567	1.000	0.375
rs17203016	0.011	0.087	0.902	0.050	0.065	0.439	-0.023	0.059	0.696	-0.025	0.066	0.708	0.071	0.047	0.125	-0.019	0.050	0.705	0.015	0.024	0.526	1.000	0.700
rs7599312	-0.005	0.189	0.978	-0.056	0.163	0.731	0.022	0.120	0.856	0.070	0.164	0.669	0.051	0.101	0.615	-0.009	0.091	0.918	0.015	0.051	0.774	1.000	0.992
rs492400	-0.048	0.071	0.504	-0.007	0.052	0.892	-0.013	0.051	0.799	-0.070	0.055	0.206	0.004	0.039	0.927	0.017	0.039	0.659	-0.010	0.020	0.604	1.000	0.840
rs2176040	0.168	0.111	0.130	0.072	0.093	0.441	-0.031	0.084	0.713	0.130	0.087	0.134	-0.006	0.062	0.923	-0.089	0.066	0.175	0.013	0.032	0.684	1.000	0.223
rs6804842	0.034	0.063	0.584	-0.054	0.047	0.246	-0.003	0.044	0.938	0.027	0.048	0.578	0.033	0.034	0.323	0.023	0.034	0.499	0.013	0.017	0.460	1.000	0.723
rs2365389	0.021	0.083	0.798	-0.001	0.063	0.981	-0.056	0.065	0.387	-0.014	0.070	0.843	-0.034	0.047	0.471	-0.046	0.049	0.352	-0.028	0.024	0.248	1.000	0.970
rs3849570	-0.021	0.058	0.724	0.057	0.043	0.190	-0.028	0.040	0.488	0.083	0.047	0.078	0.008	0.032	0.809	0.015	0.031	0.640	0.017	0.016	0.283	1.000	0.471
rs16851483	0.042	0.065	0.521	0.019	0.052	0.715	-0.027	0.045	0.551	0.108	0.052	0.036	0.069	0.037	0.066	0.055	0.037	0.143	0.046	0.019	0.014	1.000	0.448
rs1516725	0.110	0.122	0.367	0.162	0.087	0.063	-0.182	0.072	0.012	0.023	0.091	0.806	-0.076	0.058	0.188	0.010	0.055	0.850	-0.020	0.030	0.501	1.000	**0.035**
rs10938397	-0.053	0.066	0.422	0.060	0.052	0.250	-0.016	0.048	0.745	-0.028	0.052	0.585	0.071	0.036	0.050	-0.064	0.036	0.074	-0.001	0.018	0.959	1.000	0.094
rs17001654	-0.113	0.222	0.611	0.014	0.230	0.950	-0.193	0.134	0.151	-0.122	0.184	0.509	-0.035	0.111	0.755	-0.200	0.106	0.059	-0.125	0.058	0.031	1.000	0.877
rs2112347	0.004	0.057	0.941	0.015	0.045	0.738	-0.002	0.043	0.959	-0.006	0.048	0.906	0.060	0.032	0.059	0.016	0.032	0.619	0.021	0.016	0.194	1.000	0.812
rs7715256	-0.138	0.163	0.395	-0.231	0.134	0.085	0.076	0.106	0.476	0.081	0.111	0.464	0.001	0.083	0.987	0.174	0.082	0.033	0.039	0.042	0.362	1.000	0.133
rs205262	0.072	0.078	0.358	-0.163	0.062	0.008	-0.151	0.060	0.012	-0.119	0.068	0.079	-0.024	0.047	0.603	0.052	0.046	0.259	-0.046	0.023	0.046	1.000	**0.011**
rs2033529	0.138	0.078	0.079	0.074	0.060	0.216	0.043	0.055	0.440	0.031	0.062	0.618	0.039	0.043	0.367	-0.055	0.044	0.205	0.027	0.022	0.213	1.000	0.274
rs2207139	0.023	0.081	0.776	0.013	0.060	0.829	-0.075	0.064	0.240	0.054	0.069	0.428	0.001	0.046	0.984	-0.044	0.047	0.354	-0.011	0.024	0.655	1.000	0.731
rs9400239	-0.070	0.063	0.271	-0.003	0.046	0.947	-0.019	0.046	0.676	0.072	0.050	0.151	-0.003	0.035	0.937	0.044	0.034	0.189	0.011	0.017	0.515	1.000	0.445
rs9374842	0.044	0.115	0.704	0.046	0.081	0.575	-0.029	0.071	0.680	0.064	0.077	0.406	0.014	0.056	0.807	-0.006	0.054	0.906	0.014	0.028	0.624	1.000	0.949

rs13201877	-0.181	0.182	0.321	0.096	0.097	0.321	-0.155	0.103	0.131	0.081	0.111	0.465	-0.011	0.081	0.895	-0.075	0.080	0.349	-0.027	0.040	0.503	1.000	0.388
rs1167827	-0.196	0.133	0.141	-0.095	0.100	0.340	-0.060	0.095	0.532	0.062	0.089	0.486	0.010	0.066	0.882	-0.080	0.065	0.223	-0.042	0.034	0.223	1.000	0.552
rs9641123	-0.012	0.060	0.840	-0.058	0.046	0.212	0.018	0.044	0.686	-0.036	0.049	0.458	-0.040	0.035	0.257	-0.042	0.035	0.221	-0.031	0.017	0.071	1.000	0.871
rs6465468	0.011	0.312	0.971	-0.057	0.180	0.751	0.142	0.176	0.418	0.049	0.303	0.872	0.017	0.147	0.908	-0.115	0.140	0.411	-0.009	0.074	0.906	1.000	0.918
rs17405819	0.108	0.062	0.084	0.031	0.045	0.487	0.056	0.042	0.187	-0.053	0.048	0.269	0.048	0.032	0.134	-0.009	0.031	0.760	0.023	0.016	0.150	1.000	0.242
rs2033732	0.028	0.057	0.627	0.041	0.046	0.379	0.009	0.042	0.838	0.024	0.046	0.606	0.015	0.033	0.657	-0.056	0.032	0.082	0.001	0.016	0.965	1.000	0.474
rs4740619	-0.070	0.064	0.278	-0.065	0.052	0.210	-0.009	0.049	0.863	-0.041	0.054	0.449	-0.040	0.037	0.282	-0.061	0.037	0.103	-0.047	0.019	0.013	1.000	0.959
rs10968576	0.082	0.077	0.285	0.021	0.057	0.714	0.062	0.054	0.254	0.152	0.061	0.013	0.007	0.044	0.866	0.026	0.043	0.540	0.047	0.022	0.031	1.000	0.481
rs6477694	0.050	0.058	0.383	-0.009	0.044	0.838	0.051	0.042	0.217	-0.033	0.046	0.468	0.031	0.032	0.341	0.048	0.032	0.125	0.027	0.016	0.098	1.000	0.641
rs1928295	0.046	0.057	0.422	-0.002	0.044	0.958	-0.030	0.042	0.470	0.023	0.046	0.620	-0.011	0.033	0.736	-0.015	0.033	0.649	-0.005	0.016	0.770	1.000	0.894
rs10733682	0.047	0.070	0.503	0.053	0.055	0.330	0.040	0.047	0.397	-0.026	0.055	0.639	0.036	0.039	0.353	0.015	0.039	0.702	0.027	0.019	0.171	1.000	0.916
rs17094222	1.09E-04	0.060	0.999	-0.007	0.047	0.879	-0.032	0.045	0.475	-0.004	0.051	0.936	0.011	0.037	0.755	-0.051	0.037	0.167	-0.016	0.018	0.371	1.000	0.882
rs11191560	-0.061	0.064	0.342	-0.036	0.049	0.464	-0.060	0.046	0.195	0.089	0.053	0.095	0.043	0.036	0.229	0.037	0.036	0.302	0.012	0.018	0.509	1.000	0.157
rs7903146	0.335	0.212	0.114	0.022	0.134	0.867	-0.339	0.175	0.053	0.106	0.168	0.530	-0.121	0.100	0.224	-0.100	0.118	0.398	-0.057	0.056	0.313	1.000	0.164
rs4256980	-0.065	0.065	0.318	-0.037	0.044	0.404	-0.027	0.043	0.531	0.051	0.047	0.278	0.024	0.032	0.457	-0.037	0.033	0.273	-0.010	0.017	0.555	1.000	0.457
rs11030104	0.001	0.058	0.990	0.032	0.042	0.440	-0.047	0.041	0.251	0.056	0.046	0.217	-0.069	0.032	0.030	-0.016	0.032	0.621	-0.017	0.016	0.290	1.000	0.200
rs2176598	0.045	0.079	0.569	-0.094	0.064	0.138	0.007	0.063	0.918	0.002	0.068	0.971	-0.054	0.047	0.248	0.014	0.046	0.757	-0.018	0.024	0.454	1.000	0.638
rs3817334	-0.029	0.062	0.644	0.062	0.050	0.212	0.078	0.048	0.108	0.073	0.052	0.158	0.003	0.036	0.927	-0.015	0.035	0.671	0.022	0.018	0.216	1.000	0.414
rs12286929	-0.004	0.066	0.946	1.98E-04	0.049	0.997	0.017	0.050	0.727	0.017	0.051	0.732	0.007	0.035	0.851	-0.025	0.036	0.482	-4.96E-04	0.018	0.978	1.000	0.979
rs7138803	0.023	0.067	0.737	-0.054	0.051	0.293	0.080	0.046	0.084	-0.011	0.054	0.838	-0.011	0.036	0.763	0.051	0.036	0.155	0.017	0.018	0.363	1.000	0.354
rs9581854	-0.072	0.086	0.405	0.033	0.070	0.636	0.099	0.059	0.094	0.087	0.065	0.181	-0.048	0.045	0.291	-0.057	0.043	0.184	-0.004	0.023	0.849	1.000	0.137
rs12429545	0.054	0.068	0.424	-0.089	0.047	0.058	-0.010	0.050	0.841	-0.106	0.052	0.040	-0.080	0.037	0.030	-0.062	0.038	0.102	-0.061	0.019	0.001	0.858	0.376
rs1441264	0.016	0.058	0.785	0.050	0.047	0.290	-0.105	0.042	0.013	0.017	0.047	0.718	-0.001	0.035	0.985	-0.007	0.033	0.829	-0.009	0.017	0.596	1.000	0.198
rs10132280	-0.141	0.108	0.194	-0.019	0.079	0.811	0.175	0.081	0.031	0.092	0.087	0.292	-0.049	0.057	0.387	-0.004	0.055	0.937	0.006	0.029	0.837	1.000	0.144
rs12885454	-0.067	0.056	0.230	-0.003	0.044	0.947	-0.019	0.041	0.641	-0.033	0.049	0.508	-0.077	0.034	0.022	-0.028	0.032	0.377	-0.039	0.016	0.018	1.000	0.771
rs7141420	-0.035	0.060	0.558	-0.005	0.044	0.911	0.039	0.043	0.358	0.039	0.044	0.377	-0.013	0.033	0.686	-0.010	0.032	0.747	0.002	0.016	0.910	1.000	0.815
rs16951275	-0.016	0.058	0.787	0.044	0.043	0.308	-0.008	0.044	0.850	-0.035	0.046	0.448	-0.042	0.033	0.200	0.036	0.033	0.277	-0.002	0.017	0.892	1.000	0.475
rs7164727	0.047	0.070	0.497	0.015	0.050	0.768	-0.018	0.050	0.713	-0.031	0.052	0.545	-0.020	0.037	0.580	-0.015	0.035	0.675	-0.010	0.019	0.575	1.000	0.943
rs2650492	-0.179	0.228	0.432	0.140	0.097	0.149	-0.078	0.104	0.452	-0.049	0.116	0.676	-0.123	0.125	0.325	-0.144	0.113	0.205	-0.042	0.048	0.376	1.000	0.387
rs3888190	0.082	0.111	0.460	0.005	0.084	0.949	-0.137	0.073	0.061	0.005	0.078	0.954	-0.138	0.057	0.015	-0.006	0.056	0.921	-0.052	0.029	0.072	1.000	0.235
rs4787491	0.133	0.060	0.027	0.021	0.043	0.624	-0.032	0.042	0.447	0.101	0.046	0.029	0.072	0.033	0.028	0.029	0.032	0.367	0.046	0.016	0.005	1.000	0.146
rs9925964	0.020	0.090	0.822	-0.017	0.071	0.808	-0.055	0.068	0.426	-0.029	0.078	0.712	0.059	0.051	0.243	-0.029	0.049	0.553	-0.004	0.026	0.870	1.000	0.766
rs2080454	-0.027	0.060	0.651	-0.017	0.045	0.711	0.019	0.042	0.652	-0.009	0.046	0.841	-0.003	0.031	0.925	-0.032	0.032	0.310	-0.012	0.016	0.471	1.000	0.954
rs1558902	-0.022	0.085	0.794	-0.071	0.066	0.280	-0.033	0.066	0.615	0.057	0.073	0.434	-0.036	0.047	0.440	-0.007	0.050	0.883	-0.022	0.025	0.377	1.000	0.857
rs9914578	-0.045	0.074	0.543	-0.059	0.053	0.270	-0.030	0.052	0.563	0.075	0.059	0.203	-0.053	0.039	0.168	-0.015	0.038	0.693	-0.025	0.020	0.201	1.000	0.555
rs1000940	0.119	0.060	0.048	0.034	0.046	0.462	-0.017	0.042	0.686	0.092	0.046	0.045	0.012	0.033	0.721	-0.002	0.033	0.954	0.025	0.017	0.126	1.000	0.265
rs12940622	0.134	0.059	0.023	0.062	0.047	0.192	0.029	0.044	0.511	0.023	0.051	0.650	-0.010	0.034	0.779	0.002	0.035	0.951	0.025	0.017	0.146	1.000	0.361
rs7239883	-0.022	0.061	0.725	0.003	0.047	0.948	0.006	0.044	0.888	0.052	0.051	0.306	0.005	0.034	0.879	-0.060	0.034	0.074	-0.009	0.017	0.612	1.000	0.527
rs7243357	0.210	0.069	0.003	0.008	0.053	0.878	-0.049	0.059	0.406	0.046	0.057	0.422	-0.035	0.040	0.376	-0.005	0.039	0.901	0.009	0.020	0.672	1.000	0.049
rs6567160	-0.063	0.076	0.407	-0.009	0.058	0.875	0.052	0.053	0.327	-0.034	0.062	0.591	0.064	0.042	0.124	-0.018	0.045	0.690	0.011	0.022	0.603	1.000	0.517
rs29941	-0.140	0.075	0.062	-0.034	0.052	0.513	0.026	0.052	0.618	0.113	0.055	0.042	0.007	0.038	0.851	-0.015	0.036	0.673	0.001	0.019	0.965	1.000	0.129
rs2075650	-0.027	0.106	0.798	-0.021	0.081	0.796	0.103	0.071	0.151	0.047	0.091	0.607	0.154	0.161	0.339	-0.107	0.136	0.432	0.031	0.039	0.426	1.000	0.645
rs2287019	-0.104	0.075	0.169	-0.130	0.055	0.019	0.020	0.053	0.711	0.023	0.061	0.706	-0.051	0.091	0.578	0.009	0.089	0.923	-0.038	0.027	0.156	1.000	0.310
rs3810291	-3.62E-04	0.065	0.996	-0.040	0.048	0.407	0.035	0.043	0.422	-0.030	0.052	0.568	0.021	0.035	0.551	0.030	0.036	0.406	0.010	0.018	0.590	1.000	0.789
rs6091540	0.058	0.063	0.357	-0.036	0.049	0.467	-0.017	0.045	0.701	-0.101	0.053	0.057	0.002	0.036	0.959	0.009	0.036	0.805	-0.012	0.018	0.512	1.000	0.442
rs2836754	-0.028	0.059	0.638	0.019	0.045	0.671	0.077	0.042	0.069	-0.018	0.050	0.715	0.039	0.033	0.233	0.013	0.032	0.679	0.024	0.017	0.152	1.000	0.640

SCHS: Singapore Chinese Health Study; SP2: Singapore Prospective Study Program; T2D: Type II diabetes.

Age gender and total calories intake were included in the linear regression model as covariates.

S10 Table: Interaction between SNPs and %starch on BMI

	SCHS CAD cases N = 594			SCHS CAD controls N = 1070			SP2610 N = 1145			SP21m N = 949			SCHS T2D cases N = 2004			SCHS T2D controls N = 2055			SCHS + SP2610 + SP21m N = 7817				
	Beta	SE	P	Beta	SE	P	Beta	SE	P	Beta	SE	P	Beta	SE	P	Beta	SE	P	Beta	SE	P	P_{adjust}	$Q_{p\text{-value}}$
rs977747	0.212	0.157	0.177	-0.158	0.132	0.231	0.037	0.13	0.778	-0.025	0.100	0.800	0.051	0.086	0.551	0.043	0.077	0.579	0.024	0.042	0.575	1.000	0.585
rs11583200	-0.088	0.089	0.319	0.033	0.076	0.665	-0.055	0.074	0.457	-0.031	0.091	0.729	-0.047	0.063	0.452	-0.057	0.057	0.317	-0.042	0.029	0.152	1.000	0.928
rs3101336	0.042	0.108	0.696	0.088	0.087	0.309	0.101	0.079	0.200	-0.054	0.088	0.542	-0.056	0.062	0.373	0.044	0.059	0.456	0.022	0.031	0.478	1.000	0.542
rs12566985	0.068	0.076	0.367	-0.051	0.056	0.364	0.005	0.056	0.922	0.014	0.063	0.826	0.022	0.042	0.595	0.057	0.044	0.196	0.020	0.022	0.353	1.000	0.731
rs11165643	-0.147	0.076	0.052	0.028	0.058	0.635	0.007	0.051	0.894	-0.011	0.056	0.842	0.022	0.042	0.600	-0.049	0.040	0.223	-0.015	0.021	0.457	1.000	0.383
rs543874	-0.031	0.090	0.734	-0.062	0.062	0.320	-0.065	0.056	0.247	0.018	0.064	0.775	-0.041	0.043	0.338	-0.010	0.045	0.817	-0.032	0.022	0.155	1.000	0.916
rs2820292	-0.067	0.069	0.331	0.006	0.060	0.916	-4.60E-04	0.052	0.993	0.024	0.057	0.676	0.046	0.040	0.248	-0.006	0.038	0.877	0.008	0.020	0.693	1.000	0.801
rs13021737	0.157	0.107	0.143	0.017	0.088	0.844	0.010	0.078	0.897	-0.004	0.091	0.964	0.022	0.064	0.737	-0.079	0.064	0.217	0.003	0.032	0.921	1.000	0.573
rs10182181	-0.048	0.059	0.417	-0.085	0.045	0.056	0.011	0.044	0.802	0.016	0.044	0.720	0.040	0.033	0.224	0.034	0.032	0.289	0.007	0.016	0.656	1.000	0.227
rs11126666	0.073	0.061	0.232	0.024	0.047	0.607	0.046	0.047	0.320	0.144	0.049	0.004	-0.007	0.035	0.833	-0.011	0.034	0.745	0.029	0.018	0.093	1.000	0.127
rs1016287	-0.093	0.066	0.159	0.004	0.050	0.930	0.127	0.048	0.009	-0.019	0.052	0.718	-0.016	0.037	0.661	-0.012	0.038	0.750	0.003	0.019	0.892	1.000	0.100
rs11688816	-0.002	0.060	0.970	0.073	0.048	0.123	-0.010	0.047	0.833	0.014	0.050	0.786	-0.018	0.036	0.605	0.043	0.035	0.230	0.016	0.018	0.354	1.000	0.646
rs1460676	-0.004	0.061	0.951	0.006	0.047	0.905	-0.021	0.041	0.616	-0.020	0.046	0.672	0.023	0.035	0.507	-0.005	0.033	0.878	-0.001	0.017	0.931	1.000	0.970
rs1528435	0.083	0.06	0.167	-0.020	0.046	0.667	-0.054	0.044	0.224	0.029	0.047	0.536	-0.044	0.033	0.186	-0.017	0.033	0.612	-0.016	0.017	0.349	1.000	0.406
rs17203016	0.166	0.089	0.062	0.022	0.059	0.714	0.007	0.059	0.911	0.109	0.064	0.091	0.077	0.045	0.084	0.008	0.050	0.878	0.053	0.023	0.024	1.000	0.495
rs7599312	-0.207	0.198	0.295	-0.001	0.145	0.992	-0.087	0.119	0.469	0.054	0.169	0.751	-0.085	0.103	0.408	-0.051	0.089	0.571	-0.060	0.050	0.230	1.000	0.936
rs492400	0.011	0.077	0.889	0.003	0.053	0.957	-0.035	0.050	0.483	-0.052	0.052	0.320	0.021	0.04	0.608	0.031	0.040	0.434	0.001	0.020	0.966	1.000	0.795
rs2176040	0.173	0.114	0.130	0.023	0.090	0.799	0.114	0.080	0.156	0.151	0.090	0.093	-1.40E-04	0.063	0.998	0.022	0.063	0.732	0.059	0.032	0.064	1.000	0.564
rs6804842	-0.028	0.062	0.654	-0.005	0.048	0.923	0.060	0.047	0.202	-0.022	0.047	0.643	0.018	0.034	0.597	0.008	0.035	0.819	0.009	0.017	0.601	1.000	0.834
rs2365389	-0.083	0.089	0.355	0.055	0.061	0.367	-0.020	0.063	0.749	0.010	0.072	0.893	-0.043	0.046	0.353	-0.077	0.048	0.110	-0.030	0.024	0.212	1.000	0.600
rs3849570	0.021	0.056	0.703	0.060	0.043	0.164	0.003	0.041	0.944	0.088	0.048	0.065	0.002	0.031	0.956	-0.029	0.032	0.369	0.014	0.016	0.394	1.000	0.349
rs16851483	0.028	0.068	0.679	0.016	0.053	0.767	-0.017	0.048	0.719	0.055	0.052	0.292	0.057	0.038	0.133	0.017	0.039	0.663	0.027	0.019	0.152	1.000	0.864
rs1516725	0.086	0.110	0.434	0.105	0.079	0.184	-0.121	0.073	0.097	-0.074	0.091	0.417	-0.047	0.056	0.405	0.017	0.058	0.775	-0.015	0.029	0.604	1.000	0.278
rs10938397	-0.026	0.063	0.679	0.056	0.051	0.275	0.009	0.047	0.854	0.022	0.053	0.670	0.092	0.035	0.009	-0.045	0.036	0.216	0.022	0.018	0.221	1.000	0.131
rs17001654	0.091	0.196	0.642	-0.006	0.236	0.980	-0.178	0.144	0.218	-0.057	0.172	0.743	-0.079	0.105	0.452	-0.152	0.104	0.141	-0.095	0.057	0.093	1.000	0.882
rs2112347	0.001	0.059	0.982	0.001	0.046	0.978	-0.002	0.042	0.955	0.043	0.045	0.333	0.072	0.032	0.024	0.029	0.032	0.356	0.032	0.016	0.050	1.000	0.695
rs7715256	-0.169	0.149	0.256	-0.154	0.143	0.284	0.052	0.109	0.632	0.029	0.118	0.803	0.005	0.084	0.949	0.183	0.081	0.025	0.037	0.043	0.394	1.000	0.215
rs205262	0.022	0.079	0.778	-0.078	0.061	0.200	-0.062	0.063	0.327	-0.093	0.071	0.190	-0.009	0.044	0.831	0.030	0.045	0.510	-0.022	0.023	0.334	1.000	0.557
rs2033529	0.149	0.081	0.066	0.019	0.060	0.755	-0.030	0.054	0.583	0.022	0.062	0.721	-0.001	0.043	0.985	-0.013	0.045	0.768	0.008	0.022	0.716	1.000	0.567
rs2207139	0.058	0.087	0.505	0.061	0.063	0.334	-0.002	0.066	0.978	0.011	0.069	0.873	-0.029	0.045	0.527	0.011	0.046	0.813	0.009	0.024	0.703	1.000	0.887
rs9400239	0.034	0.063	0.588	0.021	0.047	0.647	-0.087	0.045	0.055	-0.079	0.049	0.103	0.046	0.036	0.199	0.022	0.033	0.515	-0.001	0.017	0.971	1.000	0.112
rs9374842	0.054	0.123	0.663	0.057	0.077	0.456	-0.100	0.068	0.139	0.009	0.080	0.912	0.015	0.058	0.791	0.031	0.055	0.569	0.006	0.028	0.825	1.000	0.655
rs13201877	-0.058	0.153	0.706	0.103	0.103	0.317	-0.124	0.102	0.226	0.081	0.112	0.471	-0.085	0.087	0.328	-0.095	0.078	0.222	-0.041	0.040	0.309	1.000	0.473
rs1167827	-0.069	0.123	0.574	-0.155	0.097	0.110	-0.004	0.092	0.964	0.001	0.088	0.992	-0.028	0.069	0.690	-0.042	0.065	0.520	-0.043	0.034	0.207	1.000	0.868
rs9641123	0.054	0.060	0.367	-0.016	0.045	0.717	0.024	0.043	0.576	-0.034	0.048	0.477	-0.026	0.036	0.467	-0.042	0.034	0.220	-0.015	0.017	0.383	1.000	0.694
rs6465468	0.068	0.290	0.816	0.024	0.148	0.872	0.191	0.152	0.209	-0.077	0.258	0.765	-0.074	0.135	0.580	-0.124	0.134	0.354	-0.009	0.066	0.894	1.000	0.716
rs17405819	0.070	0.060	0.243	0.027	0.046	0.549	0.023	0.043	0.582	-0.045	0.047	0.346	0.013	0.033	0.687	0.002	0.031	0.948	0.011	0.016	0.513	1.000	0.753
rs2033732	-0.001	0.059	0.990	0.113	0.046	0.013	0.006	0.043	0.898	0.064	0.045	0.153	0.030	0.034	0.368	-0.005	0.032	0.871	0.030	0.017	0.068	1.000	0.337
rs4740619	-0.076	0.066	0.252	-0.019	0.053	0.718	-0.001	0.049	0.985	-0.065	0.052	0.215	-0.033	0.037	0.378	-0.096	0.037	0.010	-0.050	0.019	0.008	1.000	0.650
rs10968576	0.048	0.078	0.535	0.062	0.057	0.276	-0.038	0.057	0.510	0.098	0.056	0.077	0.020	0.045	0.655	0.015	0.044	0.732	0.031	0.022	0.156	1.000	0.631
rs6477694	0.051	0.060	0.399	0.003	0.047	0.942	0.027	0.042	0.516	-0.026	0.044	0.552	0.026	0.032	0.424	0.053	0.031	0.094	0.025	0.016	0.120	1.000	0.774
rs1928295	-0.014	0.058	0.805	-0.027	0.045	0.544	0.028	0.042	0.514	0.045	0.048	0.340	0.022	0.033	0.506	0.006	0.033	0.855	0.012	0.017	0.470	1.000	0.886
rs10733682	0.132	0.070	0.062	0.041	0.053	0.436	0.003	0.046	0.954	-0.001	0.055	0.990	0.024	0.040	0.547	0.056	0.039	0.153	0.036	0.019	0.068	1.000	0.674
rs17094222	0.030	0.058	0.603	0.012	0.047	0.793	0.022	0.046	0.629	0.065	0.051	0.205	0.039	0.037	0.289	-0.020	0.037	0.578	0.021	0.018	0.254	1.000	0.804
rs11191560	-0.040	0.067	0.558	-0.063	0.051	0.213	-0.070	0.047	0.138	-0.023	0.053	0.658	0.023	0.037	0.535	0.042	0.034	0.219	-0.007	0.018	0.718	1.000	0.300
rs7903146	0.249	0.218	0.254	-0.032	0.121	0.790	-0.159	0.139	0.253	0.126	0.147	0.392	-0.201	0.104	0.053	0.044	0.115	0.703	-0.041	0.053	0.436	1.000	0.243
rs4256980	-0.035	0.063	0.576	-0.056	0.044	0.206	-0.049	0.044	0.263	0.044	0.046	0.337	-0.003	0.032	0.936	-0.025	0.034	0.464	-0.018	0.017	0.270	1.000	0.636
rs11030104	0.007	0.056	0.901	-0.009	0.043	0.833	-0.005	0.041	0.910	0.103	0.044	0.020	-0.075	0.031	0.018	-0.011	0.032	0.726	-0.010	0.016	0.529	1.000	0.055
rs2176598	0.073	0.078	0.349	0.002	0.060	0.968	-0.022	0.058	0.704	0.004	0.071	0.950	-0.074	0.045	0.102	0.014	0.046	0.760	-0.012	0.023	0.599	1.000	0.619
rs3817334	-0.020	0.061	0.743	-0.029	0.049	0.554	0.098	0.047	0.038	0.006	0.051	0.910	0.004	0.036	0.903	0.016	0.035	0.641	0.015	0.018	0.416	1.000	0.498
rs12286929	0.027	0.069	0.696	-0.048	0.048	0.312	0.038	0.049	0.441	0.021	0.051	0.684	0.028	0.035	0.425	0.017	0.036	0.636	0.014	0.018	0.427	1.000	0.826
rs7138803	0.001	0.067	0.990	-0.059	0.051	0.246	0.066	0.046	0.153	-0.041	0.054	0.451	-0.018	0.036	0.617	0.007	0.036	0.853	-0.005	0.018	0.796	1.000	0.524
rs9581854	-0.016	0.083	0.847	-0.019	0.066	0.769	-0.034	0.058	0.561	0.117	0.067	0.079	-0.002	0.046	0.967	-0.013	0.043	0.763	0.001	0.023	0.971	1.000	0.603
rs12429545	0.045	0.071	0.525	-0.076	0.049	0.117	-0.064	0.049	0.187	-0.086	0.053	0.105	-0.055	0.037	0.139	-0.026	0.037	0.488	-0.049	0.019	0.009	1.000	0.689
rs1441264	-0.021	0.058	0.716	0.023	0.047	0.630	-0.091	0.041	0.029	0.027	0.047	0.570	-0.039	0.034	0.256	0.024	0.032	0.451	-0.013	0.017	0.438	1.000	0.243
rs10132280	-0.104	0.106	0.324	-0.072	0.080	0.370	0.024	0.083	0.768	0.001	0.084	0.995	0.005	0.056	0.931	0.019	0.057	0.733	-0.008	0.029	0.785	1.000	0.861
rs12885454	-0.083	0.057	0.145	-0.017	0.044	0.697	-0.052	0.041	0.200	-0.062	0.049	0.208	-0.039	0.033	0.236	-0.020	0.032	0.524	-0.039	0.016	0.016	1.000	0.911
rs7141420	0.021	0.057	0.714	-0.032	0.043	0.461	0.024	0.043	0.578	0.028	0.046	0.542	-0.010	0.033	0.765	0.027	0.032	0.401	0.009	0.016	0.592	1.000	0.867

rs16951275	0.052	0.058	0.372	0.026	0.042	0.541	0.016	0.042	0.707	-0.023	0.047	0.634	-0.020	0.033	0.546	0.069	0.033	0.038	0.020	0.016	0.229	1.000	0.444
rs7164727	0.116	0.071	0.102	0.017	0.049	0.729	-0.022	0.048	0.652	-0.057	0.052	0.279	0.052	0.038	0.169	-0.010	0.035	0.781	0.010	0.018	0.604	1.000	0.319
rs2650492	-0.408	0.201	0.042	0.097	0.104	0.355	-0.102	0.103	0.323	0.044	0.104	0.673	-0.120	0.126	0.344	-0.060	0.114	0.600	-0.043	0.048	0.369	1.000	0.259
rs3888190	0.052	0.103	0.614	-0.073	0.077	0.344	0.001	0.072	0.987	0.043	0.075	0.567	-0.129	0.058	0.026	-0.074	0.054	0.175	-0.050	0.028	0.077	1.000	0.395
rs4787491	0.111	0.061	0.069	0.037	0.042	0.383	0.001	0.042	0.977	0.090	0.046	0.050	0.076	0.034	0.025	0.035	0.032	0.280	0.052	0.016	0.001	0.858	0.551
rs9925964	0.035	0.090	0.698	0.020	0.068	0.771	-0.002	0.070	0.974	-0.025	0.077	0.745	0.094	0.051	0.063	0.001	0.049	0.992	0.027	0.026	0.296	1.000	0.746
rs2080454	-0.011	0.061	0.859	0.020	0.045	0.648	0.028	0.042	0.497	-0.010	0.045	0.827	0.032	0.031	0.302	-0.019	0.031	0.544	0.008	0.016	0.605	1.000	0.861
rs1558902	0.026	0.080	0.746	-0.040	0.064	0.527	0.058	0.069	0.402	0.091	0.072	0.207	-0.070	0.049	0.148	-0.043	0.050	0.394	-0.014	0.025	0.566	1.000	0.381
rs9914578	-0.072	0.078	0.354	-0.030	0.055	0.589	-0.012	0.055	0.824	0.035	0.060	0.554	-0.011	0.039	0.783	-0.007	0.040	0.865	-0.011	0.020	0.578	1.000	0.930
rs1000940	0.078	0.061	0.201	0.013	0.045	0.776	-0.071	0.043	0.098	0.101	0.047	0.033	0.011	0.032	0.726	0.008	0.033	0.802	0.014	0.017	0.388	1.000	0.132
rs12940622	0.049	0.060	0.416	0.075	0.047	0.109	-0.006	0.044	0.890	0.044	0.048	0.360	0.008	0.035	0.825	0.016	0.034	0.645	0.025	0.017	0.150	1.000	0.809
rs7239883	-0.037	0.064	0.563	-0.011	0.046	0.815	-0.031	0.043	0.471	0.019	0.049	0.702	-0.002	0.034	0.949	-0.038	0.034	0.268	-0.017	0.017	0.322	1.000	0.934
rs7243357	0.126	0.064	0.051	1.28E-04	0.053	0.998	0.008	0.056	0.885	0.089	0.058	0.126	-0.039	0.041	0.334	0.007	0.039	0.861	0.016	0.020	0.421	1.000	0.259
rs6567160	0.007	0.081	0.934	-0.017	0.060	0.772	0.053	0.054	0.324	-0.020	0.065	0.759	0.013	0.043	0.760	-0.014	0.046	0.759	0.005	0.022	0.821	1.000	0.936
rs29941	-0.243	0.077	0.002	-0.028	0.048	0.569	0.002	0.051	0.965	0.085	0.057	0.134	-0.022	0.037	0.549	-0.068	0.036	0.058	-0.034	0.019	0.074	1.000	0.021
rs2075650	0.023	0.111	0.838	-0.027	0.085	0.749	0.080	0.070	0.256	-0.033	0.086	0.698	0.311	0.127	0.015	-0.005	0.147	0.975	0.043	0.039	0.267	1.000	0.279
rs2287019	-0.070	0.070	0.315	-0.079	0.054	0.145	0.022	0.051	0.668	-0.047	0.057	0.412	-0.012	0.095	0.898	0.021	0.082	0.794	-0.031	0.026	0.237	1.000	0.748
rs3810291	-0.015	0.065	0.818	0.023	0.047	0.621	-2.50E-04	0.045	0.996	0.038	0.050	0.449	0.023	0.035	0.508	0.054	0.036	0.135	0.026	0.018	0.143	1.000	0.924
rs6091540	0.019	0.062	0.754	0.004	0.049	0.939	0.072	0.047	0.130	-0.117	0.049	0.018	0.012	0.037	0.741	0.024	0.035	0.499	0.006	0.018	0.734	1.000	0.131
rs2836754	0.011	0.059	0.854	0.004	0.045	0.923	0.006	0.043	0.892	-0.002	0.050	0.976	0.022	0.033	0.507	0.045	0.033	0.167	0.020	0.017	0.241	1.000	0.959

SCHS: Singapore Chinese Health Study; SP2: Singapore Prospective Study Program; T2D: Type II diabetes.
Age gender and total calories intake were included in the linear regression model as covariates.

S11 Table: Interaction between SNPs and fiber on BMI

	SCHS CAD cases N = 594			SCHS CAD controls N = 1070			SP2610 N = 1145			SP21m N = 949			SCHS T2D cases N = 2004			SCHS T2D controls N = 2055			SCHS + SP2610 + SP21m N = 7817				
	Beta	SE	P	Beta	SE	P	Beta	SE	P	Beta	SE	P	Beta	SE	P	Beta	SE	P	Beta	SE	P	P_{adjust}	$Q_{p\text{-}value}$
rs977747	-0.110	0.145	0.448	0.140	0.111	0.209	-0.073	0.117	0.535	-0.014	0.104	0.896	0.001	0.086	0.987	0.012	0.087	0.890	0.002	0.042	0.957	1.000	0.765
rs11583200	0.009	0.099	0.928	0.005	0.079	0.950	-0.022	0.080	0.779	-0.073	0.091	0.421	-0.021	0.061	0.730	0.054	0.059	0.359	-0.001	0.030	0.982	1.000	0.889
rs3101336	0.018	0.099	0.855	-0.094	0.089	0.294	-0.002	0.077	0.978	-0.029	0.088	0.742	0.146	0.063	0.020	-0.017	0.055	0.764	0.016	0.030	0.596	1.000	0.262
rs12566985	-0.013	0.075	0.859	0.028	0.059	0.635	0.057	0.057	0.319	-0.008	0.063	0.898	-0.066	0.043	0.129	-0.056	0.043	0.193	-0.021	0.022	0.334	1.000	0.502
rs11165643	0.064	0.076	0.395	0.080	0.057	0.159	-0.002	0.056	0.969	-0.076	0.056	0.176	-0.019	0.041	0.641	0.083	0.040	0.036	0.022	0.021	0.276	1.000	0.159
rs543874	0.023	0.081	0.780	-0.012	0.065	0.859	0.033	0.059	0.576	-0.082	0.065	0.212	0.016	0.044	0.725	-0.013	0.043	0.764	-0.004	0.023	0.857	1.000	0.826
rs2820292	0.059	0.068	0.383	-0.017	0.058	0.764	-0.023	0.051	0.648	0.074	0.057	0.190	-0.003	0.041	0.943	0.070	0.039	0.070	0.027	0.020	0.176	1.000	0.514
rs13021737	-0.136	0.120	0.260	-0.026	0.090	0.772	-0.017	0.074	0.819	-0.047	0.085	0.581	0.069	0.062	0.265	0.024	0.064	0.711	0.002	0.031	0.958	1.000	0.686
rs10182181	0.084	0.059	0.151	-0.091	0.044	0.040	0.041	0.042	0.331	0.036	0.046	0.427	-0.020	0.033	0.552	-0.056	0.032	0.079	-0.015	0.016	0.374	1.000	0.062
rs11126666	-0.085	0.061	0.165	-0.009	0.049	0.852	0.052	0.046	0.267	0.044	0.048	0.352	-3.60E-04	0.034	0.992	0.006	0.033	0.859	0.007	0.017	0.703	1.000	0.556
rs1016287	0.045	0.066	0.497	-0.026	0.049	0.589	0.079	0.050	0.117	0.078	0.054	0.147	0.024	0.037	0.521	0.024	0.038	0.523	0.032	0.019	0.084	1.000	0.675
rs11688816	-0.067	0.060	0.263	-0.119	0.048	0.012	0.051	0.045	0.252	-0.050	0.049	0.302	0.007	0.036	0.847	0.024	0.035	0.493	-0.013	0.018	0.457	1.000	0.079
rs1460676	0.119	0.060	0.049	0.098	0.046	0.034	-0.079	0.043	0.065	0.006	0.045	0.902	0.012	0.033	0.715	-0.015	0.033	0.647	0.010	0.017	0.553	1.000	**0.038**
rs1528435	-0.051	0.061	0.399	0.090	0.046	0.050	0.031	0.047	0.512	0.061	0.048	0.206	0.053	0.035	0.128	-0.020	0.033	0.549	0.028	0.017	0.104	1.000	0.248
rs17203016	-0.039	0.082	0.634	0.006	0.061	0.918	-0.003	0.056	0.957	-0.026	0.064	0.684	0.034	0.043	0.423	-0.002	0.050	0.975	0.003	0.023	0.880	1.000	0.960
rs7599312	0.442	0.185	0.018	-0.064	0.163	0.693	0.124	0.123	0.312	-0.107	0.178	0.548	0.073	0.110	0.506	0.173	0.100	0.083	0.111	0.054	0.038	1.000	0.273
rs492400	-0.082	0.081	0.307	-0.029	0.053	0.587	0.046	0.049	0.344	0.127	0.053	0.016	-0.004	0.039	0.921	0.023	0.040	0.568	0.021	0.020	0.286	1.000	0.202
rs2176040	-0.089	0.118	0.452	0.036	0.089	0.685	-0.066	0.080	0.408	-0.194	0.090	0.031	0.034	0.060	0.566	-0.055	0.058	0.348	-0.041	0.031	0.186	1.000	0.350
rs6804842	-0.029	0.061	0.634	-0.036	0.048	0.456	-0.024	0.044	0.592	0.038	0.047	0.420	0.052	0.034	0.124	0.033	0.034	0.336	0.016	0.017	0.345	1.000	0.531
rs2365389	0.020	0.088	0.818	-0.050	0.059	0.400	-0.143	0.065	0.028	-0.009	0.074	0.904	-0.015	0.045	0.742	0.040	0.048	0.411	-0.021	0.024	0.378	1.000	0.346
rs3849570	-0.130	0.056	0.021	-0.001	0.044	0.983	0.007	0.040	0.857	0.107	0.046	0.021	-0.014	0.032	0.662	0.063	0.033	0.055	0.015	0.016	0.358	1.000	**0.017**
rs16851483	-0.022	0.064	0.730	0.014	0.050	0.782	0.006	0.048	0.904	0.045	0.054	0.411	-0.011	0.040	0.774	-0.025	0.037	0.491	-0.003	0.019	0.877	1.000	0.922
rs1516725	-0.003	0.109	0.977	-0.016	0.085	0.849	-0.137	0.071	0.053	-0.043	0.087	0.624	-0.016	0.057	0.775	-0.018	0.057	0.758	-0.039	0.029	0.178	1.000	0.791
rs10938397	-0.147	0.068	0.030	-0.021	0.050	0.678	0.086	0.048	0.076	0.037	0.050	0.465	-0.082	0.036	0.022	-0.001	0.037	0.984	-0.018	0.018	0.325	1.000	**0.024**
rs17001654	-0.313	0.275	0.255	0.164	0.264	0.534	0.191	0.134	0.155	-0.220	0.156	0.158	-0.057	0.123	0.645	-0.056	0.122	0.648	-0.030	0.062	0.629	1.000	0.317
rs2112347	-0.040	0.060	0.505	0.094	0.045	0.035	0.040	0.041	0.335	0.001	0.048	0.982	0.010	0.032	0.760	-0.018	0.033	0.594	0.014	0.016	0.381	1.000	0.362
rs7715256	-0.060	0.170	0.723	-0.067	0.128	0.602	-0.001	0.128	0.992	0.039	0.135	0.776	0.023	0.092	0.806	-0.032	0.082	0.696	-0.013	0.046	0.776	1.000	0.988
rs205262	0.053	0.080	0.503	-0.019	0.064	0.771	0.014	0.063	0.828	-0.086	0.068	0.212	-0.010	0.046	0.828	0.017	0.046	0.719	-0.004	0.024	0.852	1.000	0.809
rs2033529	-0.102	0.086	0.233	0.062	0.060	0.304	0.029	0.052	0.586	0.092	0.059	0.115	0.048	0.042	0.254	-0.040	0.045	0.369	0.022	0.022	0.304	1.000	0.277
rs2207139	-0.017	0.082	0.840	-0.060	0.062	0.335	-0.038	0.067	0.568	-0.005	0.066	0.937	-0.047	0.046	0.308	-0.035	0.045	0.432	-0.037	0.023	0.115	1.000	0.993
rs9400239	-0.108	0.068	0.114	-0.018	0.049	0.708	0.040	0.044	0.362	0.040	0.051	0.440	-0.041	0.035	0.240	0.019	0.034	0.572	-0.004	0.018	0.829	1.000	0.333
rs9374842	0.026	0.129	0.838	-0.071	0.074	0.338	-0.127	0.075	0.091	0.034	0.082	0.677	0.081	0.056	0.151	-0.071	0.057	0.214	-0.021	0.029	0.468	1.000	0.215
rs13201877	0.214	0.178	0.230	-0.192	0.115	0.095	-0.298	0.123	0.015	-0.070	0.116	0.547	0.035	0.096	0.717	0.146	0.079	0.063	-0.010	0.044	0.813	1.000	**0.016**
rs1167827	-0.009	0.128	0.945	-0.041	0.095	0.666	-0.027	0.087	0.754	0.022	0.095	0.817	-0.027	0.063	0.663	0.009	0.065	0.887	-0.012	0.033	0.716	1.000	0.995

rs9641123	-0.122	0.063	0.052	-0.013	0.048	0.791	-0.023	0.043	0.597	-0.026	0.050	0.605	0.062	0.036	0.080	-0.038	0.034	0.259	-0.013	0.017	0.450	1.000	0.146
rs6465468	-0.135	0.457	0.768	0.079	0.203	0.699	0.147	0.160	0.357	-0.320	0.192	0.095	0.061	0.127	0.629	-0.047	0.127	0.712	-0.003	0.068	0.959	1.000	0.515
rs17405819	0.028	0.061	0.649	-0.013	0.044	0.769	-0.030	0.044	0.486	-0.069	0.047	0.143	0.001	0.031	0.979	-0.011	0.032	0.739	-0.015	0.016	0.363	1.000	0.819
rs2033732	-0.037	0.060	0.534	-0.083	0.047	0.078	-0.017	0.042	0.682	0.022	0.046	0.627	-0.030	0.034	0.376	-0.088	0.032	0.006	-0.044	0.017	0.009	1.000	0.387
rs4740619	-0.022	0.069	0.756	-0.045	0.052	0.391	0.039	0.049	0.431	-0.003	0.057	0.951	0.011	0.038	0.772	0.057	0.038	0.135	0.015	0.019	0.429	1.000	0.677
rs10968576	0.028	0.077	0.719	0.018	0.062	0.767	0.005	0.054	0.919	0.053	0.058	0.361	0.050	0.044	0.260	-0.014	0.043	0.738	0.021	0.022	0.332	1.000	0.912
rs6477694	-0.027	0.059	0.648	-0.075	0.046	0.106	0.043	0.042	0.298	-0.075	0.047	0.114	2.54E-04	0.033	0.994	-0.020	0.031	0.519	-0.019	0.016	0.243	1.000	0.361
rs1928295	0.085	0.059	0.146	0.044	0.044	0.320	-0.018	0.042	0.675	0.016	0.047	0.741	0.004	0.033	0.908	-0.039	0.032	0.226	0.003	0.016	0.863	1.000	0.435
rs10733682	-0.137	0.069	0.046	0.024	0.053	0.650	-0.047	0.049	0.340	-0.024	0.059	0.688	0.026	0.040	0.503	-0.052	0.038	0.176	-0.025	0.020	0.200	1.000	0.316
rs17094222	0.011	0.062	0.857	-0.035	0.049	0.468	0.013	0.045	0.778	-0.002	0.052	0.976	0.026	0.038	0.488	-0.010	0.036	0.785	0.001	0.018	0.934	1.000	0.946
rs11191560	-0.032	0.068	0.636	0.035	0.051	0.497	0.007	0.046	0.877	0.082	0.052	0.115	0.032	0.037	0.382	-0.016	0.035	0.658	0.017	0.018	0.346	1.000	0.658
rs7903146	-0.056	0.187	0.765	0.060	0.113	0.593	-0.002	0.157	0.989	0.156	0.155	0.313	0.169	0.106	0.113	-0.099	0.112	0.378	0.046	0.053	0.380	1.000	0.561
rs4256980	0.038	0.060	0.531	0.028	0.043	0.513	0.002	0.041	0.958	0.054	0.048	0.260	0.041	0.031	0.190	0.012	0.032	0.716	0.027	0.016	0.093	1.000	0.952
rs11030104	-0.031	0.056	0.585	0.043	0.042	0.310	-0.030	0.043	0.477	-0.018	0.045	0.685	0.040	0.032	0.206	-0.017	0.032	0.587	0.003	0.016	0.856	1.000	0.570
rs2176598	-0.228	0.089	0.011	-0.108	0.061	0.078	0.008	0.061	0.899	0.008	0.071	0.913	0.024	0.047	0.607	-0.043	0.047	0.358	-0.035	0.024	0.136	1.000	0.126
rs3817334	-0.023	0.061	0.705	0.059	0.047	0.215	-0.027	0.045	0.546	-0.060	0.050	0.224	0.005	0.036	0.888	-0.046	0.034	0.186	-0.016	0.018	0.356	1.000	0.487
rs12286929	-0.139	0.069	0.044	0.032	0.047	0.497	-0.050	0.048	0.302	0.034	0.049	0.490	-0.046	0.036	0.201	0.013	0.037	0.720	-0.016	0.018	0.384	1.000	0.215
rs7138803	0.012	0.072	0.868	0.011	0.050	0.820	0.031	0.046	0.501	-0.079	0.052	0.130	0.005	0.037	0.889	0.046	0.036	0.200	0.011	0.018	0.553	1.000	0.529
rs9581854	-0.043	0.081	0.593	-0.059	0.066	0.375	-0.008	0.058	0.887	-0.029	0.067	0.667	-0.067	0.047	0.152	0.005	0.043	0.917	-0.030	0.023	0.189	1.000	0.899
rs12429545	0.032	0.069	0.642	0.020	0.048	0.670	-0.052	0.048	0.279	-0.034	0.057	0.549	0.008	0.038	0.832	-0.036	0.038	0.349	-0.013	0.019	0.491	1.000	0.795
rs1441264	0.007	0.061	0.911	-0.001	0.046	0.988	0.036	0.041	0.380	0.059	0.045	0.193	-0.005	0.034	0.884	-4.63E-06	0.033	1.000	0.013	0.017	0.432	1.000	0.865
rs10132280	0.105	0.122	0.389	-0.100	0.079	0.205	0.085	0.078	0.278	0.028	0.079	0.729	-0.048	0.060	0.421	-0.030	0.057	0.602	-0.012	0.030	0.692	1.000	0.492
rs12885454	0.047	0.057	0.406	0.033	0.043	0.443	-0.043	0.041	0.304	0.061	0.049	0.214	-0.005	0.032	0.875	-0.026	0.032	0.414	0.001	0.016	0.968	1.000	0.469
rs7141420	-0.027	0.060	0.658	-0.042	0.044	0.344	-0.030	0.041	0.464	-0.070	0.046	0.127	-0.006	0.033	0.846	-0.034	0.032	0.281	-0.032	0.016	0.051	1.000	0.929
rs16951275	-0.043	0.059	0.470	0.038	0.041	0.350	0.038	0.041	0.355	-0.079	0.049	0.108	0.046	0.032	0.158	-0.029	0.032	0.360	0.004	0.016	0.800	1.000	0.179
rs7164727	-0.034	0.071	0.625	-0.045	0.050	0.359	-0.030	0.049	0.540	0.082	0.051	0.110	-0.103	0.037	0.006	-0.009	0.036	0.806	-0.030	0.019	0.103	1.000	0.107
rs2650492	0.225	0.194	0.247	-0.063	0.099	0.524	0.035	0.102	0.731	-0.178	0.115	0.121	0.058	0.126	0.647	0.062	0.121	0.611	-0.007	0.048	0.891	1.000	0.449
rs3888190	0.054	0.097	0.575	0.081	0.077	0.295	-0.036	0.075	0.636	-0.005	0.087	0.958	4.25E-04	0.058	0.994	0.008	0.056	0.880	0.013	0.029	0.659	1.000	0.917
rs4787491	-0.075	0.061	0.220	-0.031	0.043	0.475	0.005	0.042	0.910	-0.002	0.047	0.968	-0.028	0.032	0.388	-0.022	0.032	0.486	-0.022	0.016	0.173	1.000	0.923
rs9925964	-0.202	0.095	0.034	-0.012	0.068	0.860	-0.022	0.069	0.751	-0.058	0.074	0.433	-0.058	0.052	0.263	-0.025	0.048	0.606	-0.048	0.026	0.063	1.000	0.648
rs2080454	0.044	0.060	0.469	-0.010	0.043	0.814	-0.006	0.041	0.877	-0.010	0.045	0.824	-0.055	0.032	0.087	0.038	0.032	0.227	-0.004	0.016	0.782	1.000	0.420
rs1558902	0.033	0.085	0.698	-0.053	0.068	0.436	-0.137	0.068	0.045	-0.102	0.080	0.198	0.046	0.047	0.331	0.062	0.050	0.218	-0.005	0.025	0.854	1.000	0.114
rs9914578	0.041	0.070	0.555	0.056	0.055	0.313	0.041	0.052	0.436	0.038	0.059	0.525	-0.039	0.040	0.325	0.026	0.040	0.518	0.018	0.020	0.375	1.000	0.703
rs1000940	-0.058	0.063	0.357	-0.004	0.044	0.921	-0.067	0.041	0.106	0.057	0.048	0.231	-0.005	0.033	0.872	0.035	0.033	0.289	-0.001	0.017	0.954	1.000	0.300
rs12940622	0.101	0.064	0.114	-0.017	0.045	0.710	0.058	0.044	0.190	0.008	0.049	0.876	0.018	0.034	0.607	-0.014	0.035	0.689	0.016	0.017	0.360	1.000	0.553
rs7239883	-0.024	0.060	0.683	-0.015	0.047	0.751	0.015	0.044	0.738	0.074	0.050	0.139	0.024	0.034	0.489	-0.065	0.035	0.064	-0.003	0.017	0.874	1.000	0.265
rs7243357	0.022	0.068	0.742	-0.039	0.053	0.463	0.014	0.058	0.811	-0.082	0.056	0.142	0.042	0.040	0.300	0.016	0.040	0.693	0.002	0.020	0.932	1.000	0.537
rs6567160	-0.056	0.084	0.504	0.058	0.058	0.315	0.045	0.058	0.433	0.181	0.064	0.005	0.001	0.043	0.985	-0.021	0.044	0.634	0.028	0.022	0.207	1.000	0.118
rs29941	0.086	0.071	0.231	0.045	0.052	0.386	0.038	0.051	0.463	0.001	0.058	0.980	0.029	0.039	0.447	0.045	0.037	0.225	0.038	0.019	0.050	1.000	0.967
rs2075650	-0.153	0.116	0.186	0.021	0.084	0.806	-0.003	0.079	0.969	-0.273	0.092	0.003	-0.290	0.124	0.019	-0.166	0.148	0.261	-0.113	0.041	0.006	1.000	0.081
rs2287019	0.116	0.075	0.123	-0.014	0.054	0.789	-0.071	0.054	0.187	-0.040	0.061	0.518	-0.035	0.088	0.691	-0.003	0.083	0.972	-0.017	0.027	0.513	1.000	0.500
rs3810291	0.083	0.062	0.185	-0.053	0.047	0.266	0.014	0.046	0.759	0.018	0.050	0.720	-0.028	0.036	0.435	-0.080	0.037	0.030	-0.022	0.018	0.214	1.000	0.220
rs6091540	0.086	0.063	0.170	-0.075	0.048	0.120	0.081	0.048	0.089	0.066	0.050	0.182	-0.032	0.036	0.376	-0.065	0.035	0.063	-0.008	0.018	0.646	1.000	**0.023**
rs2836754	0.049	0.059	0.401	0.043	0.047	0.351	0.057	0.044	0.189	-0.014	0.047	0.771	0.016	0.033	0.619	-0.037	0.033	0.250	0.011	0.017	0.524	1.000	0.473

SCHS: Singapore Chinese Health Study; SP2: Singapore Prospective Study Program; T2D: Type II diabetes.

Age gender and total calories intake were included in the linear regression model as covariates.

S12 Table: Interaction between SNPs and cholesterol on BMI

	SCHS CAD cases N = 594			SCHS CAD controls N = 1070			SP2610 N = 1145			SP21m N = 949			SCHS T2D cases N = 2004			SCHS T2D controls N = 2055			SCHS + SP2610 + SP21m N = 7817				
	Beta	SE	P	Beta	SE	P	Beta	SE	P	Beta	SE	P	Beta	SE	P	Beta	SE	P	Beta	SE	P	P_{adjust}	$Q_{p\text{-}value}$
rs977747	0.020	0.176	0.912	0.190	0.109	0.081	-0.084	0.109	0.441	0.036	0.100	0.721	0.030	0.081	0.711	-0.031	0.084	0.709	0.022	0.041	0.585	1.000	0.584
rs11583200	0.164	0.093	0.077	0.003	0.083	0.974	0.027	0.081	0.743	0.132	0.092	0.153	0.046	0.059	0.430	0.113	0.059	0.056	0.077	0.030	0.011	1.000	0.689
rs3101336	-0.088	0.113	0.440	-0.164	0.079	0.037	-0.126	0.071	0.077	0.054	0.091	0.549	-0.011	0.060	0.851	-0.033	0.056	0.554	-0.056	0.029	0.055	1.000	0.399
rs12566985	0.014	0.073	0.845	0.078	0.057	0.173	0.042	0.055	0.446	0.004	0.059	0.950	-0.006	0.041	0.888	0.004	0.043	0.924	0.018	0.021	0.397	1.000	0.877
rs11165643	0.055	0.077	0.477	-0.052	0.057	0.367	0.050	0.050	0.315	0.002	0.054	0.970	-0.033	0.042	0.424	-0.026	0.043	0.54	-0.008	0.021	0.691	1.000	0.671
rs543874	0.134	0.093	0.151	-0.010	0.064	0.874	-0.059	0.057	0.301	0.044	0.064	0.496	-0.009	0.046	0.847	0.006	0.042	0.88	0.002	0.023	0.915	1.000	0.597
rs2820292	0.094	0.066	0.154	-0.023	0.06	0.694	0.026	0.054	0.633	-0.080	0.056	0.156	0.021	0.041	0.607	-0.083	0.039	0.031	-0.018	0.020	0.365	1.000	0.129
rs13021737	-0.067	0.116	0.563	-0.098	0.088	0.269	0.104	0.075	0.168	0.107	0.088	0.224	-0.001	0.066	0.990	-0.085	0.068	0.209	-0.004	0.032	0.898	1.000	0.261
rs10182181	0.035	0.057	0.536	0.050	0.045	0.269	-0.055	0.042	0.187	-0.052	0.046	0.257	0.019	0.033	0.578	0.026	0.032	0.407	0.006	0.016	0.717	1.000	0.358
rs11126666	-0.105	0.058	0.071	-0.010	0.046	0.835	-0.002	0.045	0.960	-0.025	0.051	0.619	-0.003	0.034	0.941	0.049	0.035	0.165	-0.003	0.017	0.871	1.000	0.362
rs1016287	0.071	0.064	0.266	-0.041	0.050	0.413	-0.040	0.052	0.438	0.055	0.052	0.288	0.017	0.036	0.634	-0.019	0.037	0.603	0.002	0.019	0.919	1.000	0.531
rs11688816	0.161	0.062	0.009	-0.003	0.047	0.943	0.005	0.045	0.913	0.013	0.051	0.801	-0.069	0.036	0.059	0.011	0.034	0.746	0.002	0.018	0.916	1.000	0.060
rs1460676	0.006	0.061	0.922	-0.010	0.045	0.827	0.066	0.042	0.112	0.003	0.045	0.951	-0.057	0.034	0.089	0.069	0.033	0.034	0.014	0.017	0.398	1.000	0.098
rs1528435	4.74E-04	0.063	0.994	-0.035	0.045	0.437	-0.040	0.045	0.377	0.014	0.048	0.767	-0.001	0.034	0.967	-2.2E-05	0.034	0.999	-0.009	0.017	0.584	1.000	0.947
rs17203016	-0.04	0.092	0.667	-0.090	0.062	0.146	-0.106	0.062	0.085	0.071	0.070	0.308	-0.035	0.045	0.439	0.001	0.049	0.989	-0.033	0.024	0.162	1.000	0.420
rs7599312	0.105	0.249	0.672	-0.166	0.176	0.345	-0.162	0.122	0.184	-0.119	0.161	0.458	-0.062	0.116	0.595	0.151	0.100	0.129	-0.026	0.055	0.638	1.000	0.330
rs492400	0.018	0.074	0.807	0.118	0.056	0.035	0.007	0.051	0.892	-0.048	0.052	0.356	-0.020	0.038	0.608	-0.029	0.039	0.460	-0.002	0.020	0.905	1.000	0.287
rs2176040	0.029	0.121	0.814	-0.125	0.088	0.155	-0.024	0.079	0.764	0.003	0.087	0.973	0.032	0.065	0.618	-0.067	0.061	0.275	-0.028	0.032	0.379	1.000	0.725
rs6804842	0.043	0.062	0.485	-0.037	0.047	0.433	0.039	0.045	0.388	0.015	0.048	0.759	-0.047	0.034	0.166	0.011	0.034	0.734	-0.003	0.017	0.850	1.000	0.558
rs2365389	0.115	0.080	0.152	-0.039	0.061	0.519	0.053	0.064	0.407	-0.069	0.070	0.328	0.007	0.049	0.879	-0.020	0.048	0.677	3.28E-04	0.024	0.989	1.000	0.506
rs3849570	0.043	0.059	0.464	0.014	0.045	0.756	-0.008	0.040	0.851	-0.049	0.046	0.290	-0.006	0.032	0.854	-0.017	0.032	0.606	-0.008	0.016	0.624	1.000	0.870
rs16851483	0.067	0.066	0.308	0.037	0.051	0.470	-0.028	0.047	0.551	-0.041	0.057	0.474	-0.094	0.038	0.015	-0.009	0.038	0.809	-0.024	0.019	0.209	1.000	0.231
rs1516725	-0.078	0.109	0.472	-0.069	0.081	0.395	0.247	0.082	0.003	0.091	0.086	0.292	0.008	0.055	0.886	-0.057	0.053	0.285	0.012	0.029	0.674	1.000	0.029
rs10938397	0.142	0.065	0.031	0.055	0.049	0.267	-0.041	0.047	0.381	-0.070	0.053	0.187	-0.048	0.037	0.204	0.092	0.036	0.010	0.017	0.018	0.351	1.000	0.007
rs17001654	-0.031	0.224	0.892	0.204	0.276	0.461	0.177	0.130	0.174	0.120	0.164	0.462	-0.072	0.104	0.490	0.123	0.099	0.213	0.071	0.056	0.202	1.000	0.646
rs2112347	0.066	0.057	0.244	-0.007	0.046	0.878	-0.111	0.043	0.010	-0.034	0.047	0.472	-0.059	0.032	0.062	0.004	0.031	0.891	-0.030	0.016	0.066	1.000	0.119
rs7715256	0.245	0.173	0.158	0.059	0.138	0.667	-0.049	0.113	0.662	-0.215	0.124	0.084	0.043	0.086	0.623	-0.126	0.078	0.103	-0.041	0.043	0.342	1.000	0.195
rs205262	-0.085	0.079	0.282	0.206	0.065	0.002	0.065	0.06	0.274	0.082	0.068	0.230	0.102	0.047	0.030	-0.022	0.046	0.640	0.059	0.023	0.012	1.000	0.029
rs2033529	-0.088	0.087	0.312	-0.081	0.061	0.184	-0.053	0.056	0.348	-0.152	0.062	0.014	-0.105	0.042	0.012	0.025	0.044	0.567	-0.066	0.022	0.003	1.000	0.200
rs2207139	0.028	0.084	0.734	-0.059	0.067	0.385	0.086	0.063	0.171	-0.062	0.062	0.323	0.050	0.047	0.290	-0.004	0.046	0.927	0.010	0.024	0.675	1.000	0.456
rs9400239	0.146	0.065	0.025	0.024	0.047	0.602	-0.066	0.046	0.155	-0.081	0.052	0.123	-0.025	0.035	0.481	-0.003	0.033	0.928	-0.011	0.017	0.513	1.000	0.082
rs9374842	-0.295	0.124	0.018	0.027	0.078	0.728	-0.032	0.072	0.659	-0.070	0.082	0.389	0.001	0.060	0.984	0.089	0.056	0.113	-0.002	0.029	0.935	1.000	0.103
rs13201877	-0.225	0.176	0.201	0.104	0.103	0.314	0.171	0.109	0.118	-0.104	0.122	0.398	0.055	0.090	0.542	0.006	0.085	0.940	0.033	0.043	0.449	1.000	0.345
rs1167827	0.218	0.138	0.116	0.007	0.089	0.935	-0.028	0.086	0.749	-0.038	0.092	0.678	0.030	0.062	0.630	0.102	0.066	0.119	0.039	0.033	0.242	1.000	0.542
rs9641123	-0.061	0.065	0.349	0.030	0.048	0.529	-0.016	0.044	0.721	0.038	0.049	0.441	-0.001	0.035	0.976	0.072	0.034	0.037	0.020	0.018	0.246	1.000	0.414
rs6465468	0.595	0.334	0.076	0.020	0.214	0.924	-0.042	0.207	0.838	0.376	0.234	0.108	-0.032	0.155	0.834	0.213	0.136	0.118	0.130	0.077	0.093	1.000	0.361
rs17405819	-0.046	0.058	0.427	-0.049	0.046	0.292	0.054	0.042	0.196	0.040	0.046	0.384	-0.035	0.031	0.263	0.017	0.031	0.587	-0.001	0.016	0.942	1.000	0.334
rs2033732	0.026	0.058	0.657	0.004	0.046	0.925	0.049	0.044	0.269	-0.032	0.046	0.488	0.027	0.033	0.416	0.036	0.031	0.249	0.022	0.017	0.184	1.000	0.831
rs4740619	0.087	0.066	0.186	0.109	0.053	0.039	0.077	0.049	0.119	0.103	0.056	0.064	0.033	0.037	0.381	0.092	0.038	0.015	0.077	0.019	**5.01E-05**	**0.043**	0.828
rs10968576	-0.046	0.074	0.539	-0.001	0.058	0.984	-0.096	0.058	0.097	-0.082	0.064	0.207	-0.012	0.044	0.780	-0.034	0.043	0.429	-0.039	0.022	0.073	1.000	0.817
rs6477694	-0.043	0.061	0.482	0.058	0.045	0.199	-0.049	0.041	0.228	0.073	0.047	0.122	-0.050	0.033	0.123	-0.028	0.032	0.384	-0.015	0.016	0.373	1.000	0.138
rs1928295	-0.062	0.061	0.315	0.061	0.045	0.169	0.030	0.042	0.473	0.033	0.046	0.467	-0.019	0.033	0.568	0.027	0.033	0.404	0.015	0.017	0.357	1.000	0.531
rs10733682	-0.009	0.072	0.905	-0.006	0.054	0.907	-0.073	0.048	0.129	-0.015	0.056	0.792	0.005	0.039	0.891	0.014	0.039	0.710	-0.010	0.020	0.600	1.000	0.809
rs17094222	0.026	0.061	0.666	0.055	0.048	0.253	-0.060	0.044	0.176	0.011	0.053	0.828	-0.014	0.038	0.707	0.012	0.036	0.733	0.001	0.018	0.944	1.000	0.604
rs11191560	-0.040	0.067	0.550	0.047	0.053	0.371	-0.020	0.044	0.646	-0.027	0.054	0.623	-0.073	0.037	0.050	-0.020	0.035	0.557	-0.027	0.018	0.138	1.000	0.608
rs7903146	-0.206	0.219	0.346	-0.030	0.119	0.801	-0.225	0.195	0.249	0.042	0.136	0.755	-0.071	0.095	0.456	0.186	0.113	0.100	-0.009	0.053	0.864	1.000	0.345
rs4256980	0.012	0.064	0.854	-0.017	0.044	0.702	0.043	0.043	0.311	-0.078	0.045	0.079	-0.007	0.032	0.825	-0.009	0.033	0.777	-0.010	0.016	0.543	1.000	0.541
rs11030104	-0.031	0.057	0.584	-0.027	0.041	0.509	-0.039	0.041	0.342	0.025	0.045	0.587	0.024	0.031	0.451	0.043	0.031	0.169	0.008	0.016	0.628	1.000	0.526
rs2176598	-0.088	0.082	0.284	0.033	0.060	0.582	0.015	0.061	0.806	0.013	0.071	0.850	0.037	0.047	0.433	0.034	0.046	0.454	0.020	0.023	0.403	1.000	0.846
rs3817334	0.048	0.062	0.432	-0.012	0.047	0.792	-0.008	0.048	0.873	-0.108	0.051	0.034	-0.025	0.035	0.486	-0.037	0.034	0.278	-0.028	0.018	0.115	1.000	0.496
rs12286929	0.087	0.067	0.193	-0.013	0.050	0.799	0.026	0.049	0.594	-0.108	0.052	0.038	-0.040	0.036	0.267	0.030	0.036	0.406	-0.008	0.018	0.677	1.000	0.150
rs7138803	0.072	0.072	0.313	-0.006	0.050	0.900	0.052	0.046	0.261	0.068	0.055	0.219	0.008	0.037	0.832	-0.035	0.035	0.322	0.012	0.018	0.505	1.000	0.494
rs9581854	0.001	0.084	0.986	-0.130	0.068	0.056	-0.034	0.059	0.566	-0.109	0.065	0.093	0.048	0.046	0.293	0.014	0.044	0.749	-0.018	0.023	0.439	1.000	0.192
rs12429545	0.008	0.068	0.908	0.019	0.049	0.699	-0.048	0.049	0.326	0.076	0.055	0.165	0.089	0.037	0.017	0.060	0.037	0.102	0.043	0.019	0.021	1.000	0.299
rs1441264	0.064	0.058	0.276	-0.030	0.044	0.486	-0.018	0.044	0.679	0.009	0.047	0.854	-0.010	0.035	0.769	0.020	0.033	0.543	0.002	0.017	0.899	1.000	0.803
rs10132280	0.081	0.100	0.420	-0.039	0.075	0.605	-0.273	0.076	<0.001	-0.123	0.089	0.168	0.049	0.059	0.403	-0.006	0.058	0.919	-0.042	0.029	0.147	1.000	0.013
rs12885454	0.019	0.057	0.745	0.037	0.044	0.404	-0.002	0.040	0.966	-0.059	0.049	0.231	0.036	0.032	0.251	0.054	0.032	0.095	0.023	0.016	0.159	1.000	0.499
rs7141420	0.174	0.059	0.004	-0.002	0.045	0.959	-0.032	0.042	0.455	-0.018	0.044	0.678	0.058	0.033	0.083	-0.010	0.032	0.753	0.017	0.016	0.298	1.000	0.046

rs16951275	0.071	0.057	0.209	-0.067	0.042	0.110	-0.046	0.043	0.279	0.035	0.048	0.462	-0.029	0.033	0.374	-0.058	0.032	0.069	-0.029	0.016	0.074	1.000	0.240
rs7164727	-0.082	0.070	0.243	0.036	0.050	0.476	-0.034	0.049	0.491	-0.024	0.049	0.632	0.061	0.036	0.094	0.045	0.035	0.198	0.019	0.018	0.303	1.000	0.308
rs2650492	0.057	0.136	0.678	0.076	0.100	0.449	0.107	0.096	0.264	-0.024	0.099	0.806	-0.017	0.116	0.885	-0.061	0.131	0.642	0.030	0.045	0.501	1.000	0.871
rs3888190	-0.108	0.110	0.326	-0.056	0.093	0.548	0.100	0.077	0.193	0.039	0.078	0.621	0.094	0.058	0.105	-0.042	0.056	0.452	0.019	0.029	0.514	1.000	0.302
rs4787491	-0.017	0.058	0.770	0.034	0.043	0.420	0.024	0.041	0.554	-0.043	0.046	0.349	-0.049	0.033	0.138	0.006	0.033	0.853	-0.008	0.016	0.604	1.000	0.559
rs9925964	0.025	0.098	0.800	-0.026	0.067	0.699	0.038	0.065	0.562	0.087	0.072	0.226	-0.051	0.050	0.313	0.002	0.048	0.966	0.002	0.025	0.933	1.000	0.695
rs2080454	-1.30E-04	0.059	0.998	-0.012	0.043	0.788	-0.010	0.04	0.797	0.044	0.046	0.340	0.015	0.032	0.631	0.023	0.032	0.461	0.012	0.016	0.457	1.000	0.938
rs1558902	-0.011	0.083	0.891	0.002	0.063	0.976	0.136	0.067	0.042	0.120	0.080	0.136	0.040	0.046	0.384	0.034	0.050	0.497	0.049	0.025	0.048	1.000	0.593
rs9914578	0.032	0.069	0.638	0.067	0.054	0.219	0.065	0.053	0.214	-0.126	0.059	0.032	0.079	0.039	0.039	-0.006	0.040	0.882	0.026	0.020	0.186	1.000	0.064
rs1000940	-0.129	0.064	0.043	-0.111	0.046	0.015	0.006	0.042	0.878	-0.036	0.048	0.452	-0.008	0.033	0.821	-0.023	0.033	0.482	-0.035	0.017	0.036	1.000	0.240
rs12940622	-0.159	0.062	0.011	0.002	0.048	0.97	-0.081	0.046	0.076	0.021	0.050	0.680	0.022	0.035	0.531	-0.035	0.035	0.312	-0.025	0.018	0.150	1.000	0.101
rs7239883	-0.032	0.065	0.621	-0.026	0.048	0.593	-0.018	0.043	0.683	-0.065	0.050	0.193	-0.031	0.034	0.367	0.036	0.034	0.290	-0.015	0.017	0.384	1.000	0.613
rs7243357	-0.156	0.073	0.033	0.025	0.050	0.611	0.086	0.057	0.134	-0.045	0.056	0.426	0.081	0.040	0.046	-4.80E-04	0.039	0.990	0.017	0.020	0.402	1.000	0.051
rs6567160	-0.010	0.076	0.891	-0.018	0.061	0.762	-0.051	0.055	0.353	-0.124	0.062	0.046	-0.079	0.041	0.056	-0.002	0.045	0.973	-0.049	0.022	0.025	1.000	0.609
rs29941	0.072	0.074	0.329	0.073	0.052	0.158	-0.040	0.051	0.426	0.042	0.056	0.459	0.051	0.039	0.189	0.012	0.037	0.737	0.031	0.019	0.115	1.000	0.624
rs2075650	0.201	0.108	0.065	0.013	0.088	0.884	-0.070	0.079	0.374	0.120	0.089	0.179	-0.208	0.142	0.143	0.056	0.128	0.660	0.026	0.041	0.525	1.000	0.157
rs2287019	0.165	0.077	0.032	0.092	0.052	0.078	-0.039	0.055	0.478	-0.024	0.059	0.692	-0.017	0.089	0.847	-0.019	0.086	0.828	0.027	0.027	0.315	1.000	0.187
rs3810291	0.017	0.060	0.775	0.014	0.048	0.775	7.49E-05	0.044	0.999	0.010	0.051	0.851	0.012	0.036	0.733	0.001	0.036	0.969	0.008	0.018	0.657	1.000	1.000
rs6091540	0.016	0.061	0.791	0.088	0.048	0.068	0.063	0.046	0.174	-0.045	0.050	0.369	0.030	0.036	0.401	0.025	0.036	0.478	0.031	0.018	0.082	1.000	0.512
rs2836754	-0.098	0.058	0.092	-0.012	0.047	0.798	-0.086	0.042	0.043	-0.019	0.049	0.695	-0.042	0.032	0.189	0.019	0.033	0.560	-0.031	0.017	0.059	1.000	0.337

SCHS: Singapore Chinese Health Study; SP2: Singapore Prospective Study Program; T2D: Type II diabetes.
Age gender and total calories intake were included in the linear regression model as covariates.

S13 Table: Interaction between GRS and dietary factors on BMI in individual datasets used in the study.

	SCHS CAD cases N = 594			SCHS CAD controls N = 1070			SP2610 N = 1145			SP21m N = 949			SCHS T2D cases N = 2004			SCHS T2D controls N = 2055			Meta-analysis of all datasets N = 7817			
	Beta	SE	P	Beta	SE	P	Beta	SE	P	Beta	SE	P	Beta	SE	P	Beta	SE	P	Beta	SE	P	$Q_{p\text{-value}}$
unprocessed (servings/day)	-0.254	0.308	0.411	-0.100	0.251	0.704	0.108	0.101	0.283	0.089	0.079	0.262	0.105	0.174	0.545	-0.437	0.196	0.026	0.037	0.054	0.488	0.152
processed (servings/day)	1.545	0.792	0.051	0.444	0.584	0.447	0.225	0.207	0.276	0.252	0.242	0.298	0.560	0.295	0.058	0.313	0.327	0.339	0.345	0.123	0.005	0.646
Total (servings/day)	0.006	0.257	0.982	-0.007	0.211	0.973	0.102	0.082	0.211	0.075	0.069	0.277	0.174	0.134	0.194	-0.178	0.150	0.242	0.066	0.045	0.140	0.603
rs4740619 x cholesterol adjusted for processed red meat intake	0.086	0.066	0.196	0.111	0.053	0.036	0.078	0.049	0.112	0.103	0.056	0.064	0.033	0.037	0.381	0.092	0.038	0.015	0.078	0.019	4.74E-05	0.822
rs4746019 x processed red meat adjusted for cholesterol intake	1.603	0.792	0.044	0.458	0.586	0.435	0.193	0.207	0.351	0.244	0.242	0.314	0.556	0.296	0.060	0.318	0.327	0.332	0.333	0.123	0.007	0.581
egg yolk (grams/day)	0.030	0.014	0.035	0.007	0.010	0.496	0.007	0.005	0.170	0.002	0.006	0.706	0.006	0.008	0.396	-0.002	0.009	0.986	0.006	0.003	0.047	0.584
rs4740619 x cholesterol adjusted for egg yolk intake	0.082	0.066	0.216	0.099	0.053	0.061	0.079	0.049	0.110	0.102	0.056	0.068	0.031	0.037	0.407	0.090	0.038	0.017	0.074	0.019	9.07E-05	0.849
rs4746019 x egg yolk adjusted for cholesterol intake	0.030	0.014	0.039	0.007	0.011	0.540	0.008	0.005	0.110	0.002	0.006	0.699	0.006	0.008	0.411	-1.50E-04	0.010	0.987	0.006	0.003	0.037	0.595

Results are from meta-analysis of SCHS MI cases (N = 594) and control datasets (N = 1070), SP2 610 (N = 1145) and 1M datasets (N = 949), SCHS Type 2 diabetes cases (N = 2004) and control datasets (N = 2055). $Q_{p\text{-value}}$: Cochran's Q heterogeneity measure.
SCHS: Singapore Chinese Health Study; SP2: Singapore Prospective Study Program; T2D: Type II diabetes.
Age, gender and total calories intake were included in the linear regression model as covariates.
